# mTOR intersects antibody-inducing signals from TACI in marginal zone B cells

**DOI:** 10.1038/s41467-017-01602-4

**Published:** 2017-11-13

**Authors:** Jordi Sintes, Maurizio Gentile, Shuling Zhang, Yolanda Garcia-Carmona, Giuliana Magri, Linda Cassis, Daniel Segura-Garzón, Alessandra Ciociola, Emilie K. Grasset, Sabrina Bascones, Laura Comerma, Marc Pybus, David Lligé, Irene Puga, Cindy Gutzeit, Bing He, Wendy DuBois, Marta Crespo, Julio Pascual, Anna Mensa, Juan Ignacio Aróstegui, Manel Juan, Jordi Yagüe, Sergi Serrano, Josep Lloreta, Eric Meffre, Michael Hahne, Charlotte Cunningham-Rundles, Beverly A. Mock, Andrea Cerutti

**Affiliations:** 1 Program for Inflammatory and Cardiovascular Disorders, Institut Hospital del Mar d’Investigacions Mèdiques (IMIM), Barcelona, 08003 Spain; 2 Laboratory of Cancer Biology and Genetics, National Cancer Institute, National Institutes of Health, Bethesda, MD 20892 USA; 3 Department of Medicine and Pediatrics, Immunology Institute, Icahn School of Medicine at Mount Sinai, New York, NY 10029 USA; 4 Department of Medicine, Immunology Institute, Icahn School of Medicine at Mount Sinai, New York, NY 10029 USA; 5 Department of Medicine, Center for Molecular Medicine at Karolinska University Hospital, Karolinska Institutet, Stockholm, 171 76 Sweden; 6 Department of Nephrology, Institut Hospital del Mar d’Investigacions Mèdiques (IMIM), Barcelona, 08003 Spain; 7 Immunology Service, Hospital Clínic of Barcelona, Barcelona, 08036 Spain; 8 Department of Pathology, Hospital del Mar, Barcelona, 08003 Spain; 9 Universitat Pompeu Fabra, Barcelona, 08003 Spain; 10 Department of Immunobiology, Yale University, New Haven, CT 06511 USA; 11 Molecular Genetics Institute of Montpellier, Montpellier, 34293 France; 12 Catalan Institute for Research and Advanced Studies (ICREA), Barcelona, 08003 Spain

## Abstract

Mechanistic target of rapamycin (mTOR) enhances immunity in addition to orchestrating metabolism. Here we show that mTOR coordinates immunometabolic reconfiguration of marginal zone (MZ) B cells, a pre-activated lymphocyte subset that mounts antibody responses to T-cell-independent antigens through a Toll-like receptor (TLR)-amplified pathway involving transmembrane activator and CAML interactor (TACI). This receptor interacts with mTOR via the TLR adapter MyD88. The resulting mTOR activation instigates MZ B-cell proliferation, immunoglobulin G (IgG) class switching, and plasmablast differentiation through a rapamycin-sensitive pathway that integrates metabolic and antibody-inducing transcription programs, including NF-κB. Disruption of TACI–mTOR interaction by rapamycin, truncation of the MyD88-binding domain of TACI, or B-cell-conditional mTOR deficiency interrupts TACI signaling via NF-κB and cooperation with TLRs, thereby hampering IgG production to T-cell-independent antigens but not B-cell survival. Thus, mTOR drives innate-like antibody responses by linking proximal TACI signaling events with distal immunometabolic transcription programs.

## Abstract (web summary)

Marginal zone B cells differentiate into plasma cells rapidly in response to T-cell-independent antigens, but how they do so is not clear. Here the authors show TACI cooperates with TLR signalling to drive mTOR activity and subsequent class switching and plasmablast differentiation.

## Introduction

Marginal zone (MZ) B cells inhabit a splenic area intercalated between the circulation and the immune system and mount rapid immunoglobulin M (IgM) and IgG responses to blood-borne antigens^1^. Unlike follicular B cells, which follow a T-cell-dependent pathway requiring CD40 ligand (CD40L), MZ B cells follow a T-cell-independent pathway involving B-cell-activating factor of the tumor necrosis family (BAFF) and a proliferation-inducing ligand (APRIL)^1,2^. These CD40L-related cytokines derive from innate immune cells and activate MZ B cells via transmembrane activator and CAML interactor (TACI)^3–6^, a receptor that induces antibody production in concert with B-cell antigen receptor (BCR) and Toll-like receptors (TLR)^7^.

Compared with follicular B cells, MZ B cells are in an elusive pre-activation state encompassing lower BCR activation thresholds and higher TACI and TLR expression^1^. This innate-like configuration poises MZ B cells to quickly differentiate into plasmablasts^8^. In addition to undergoing explosive proliferation and massive IgM secretion, plasmablasts initiate IgM-to-IgG class switch recombination (CSR) and even some degree of Ig gene somatic hypermutation (SHM)^3,9,10^. In general, CSR and SHM unfold in the germinal center to generate class-switched antibodies with higher affinity for antigen, but become extinct in plasma cells (PC) expressing high levels of B-lymphocyte-induced maturation protein-1 (BLIMP-1)^11^. Besides activating X box protein-1 (XBP-1)-regulated unfolded protein response (UPR) programs required for antibody synthesis and secretion^12^, BLIMP-1 transcriptionally suppresses paired-box containing-5 (PAX5)-orchestrated B-cell identity programs involved in B-cell proliferation, CSR and SHM^13^.

While the regulation of plasmablast induction is relatively well understood, the inductive phase of MZ B-cell responses is unclear. Dendritic cell (DC) and T-cell activation involves metabolic reprogramming via mechanistic target of rapamycin (mTOR)^14,15^, a serine–threonine kinase that forms mTORC1 and mTORC2 complexes activated by phosphatidylinositol 3-kinase (PI3K)-induced AKT kinases^16^. Unlike mTORC2, mTORC1 is inhibited by rapamycin and mostly regulates cell metabolism^17^. Aside from lipid and nucleic acid synthesis, mTORC1 enhances protein synthesis by suppressing inhibitors of eukaryotic translation initiation factor 4E (eIF4E) and activating ribosomal S6 inducers of protein translation^16^. mTORC1 coordinates these anabolic processes with nutrient intake, glycolysis, and mitochondrial respiration, as well as mitochondrial, endoplasmic reticulum (ER), ribosome, and lysosome biogenesis, through various transcription factors, including sterol regulatory element-binding proteins (SREBP), peroxisome proliferator-activated receptor-γ (PPARγ), hypoxia-inducible factor 1α (HIF1α) and MYC^14,16^.

mTORC1 additionally shapes immune responses by regulating the activation of DC and T-cell-activating transcription factors such as interferon regulatory factor (IRF), signal transducer and activator of transcription proteins (STATs), and nuclear factor-κB (NF-κB)^14,15,18^. Moreover, mTORC1 enhances follicular B-cell responses to T-cell-dependent antigens^19–21^, whereas mTORC2 promotes BCR-induced entry of follicular B cells into the cell cycle via AKT-dependent degradation of forkhead box O1 (FOXO1)^22^. Although MZ B-cell development is regulated by mTORC1^23^, how mTOR is linked to antibody-inducing receptors such as TACI is not known^24^. Identifying this mechanism could support the use of mTOR inhibitors in autoantibody disorders involving abnormal activation of pathological MZ B cells by TACI^5,25,26^.

Here we show that mTOR interacts with TACI through the TLR adapter MyD88. By linking proximal TACI signaling events with downstream metabolic and immune transcription programs, mTOR signals from TACI contribute to the pre-activated state of MZ B cells and promote MZ B-cell induction of homeostatic as well as post-immune IgM and IgG responses against T-cell-independent antigens.

## Results

### MZ B cells have elevated TACI expression and mTOR signatures

**Fig. 1:**
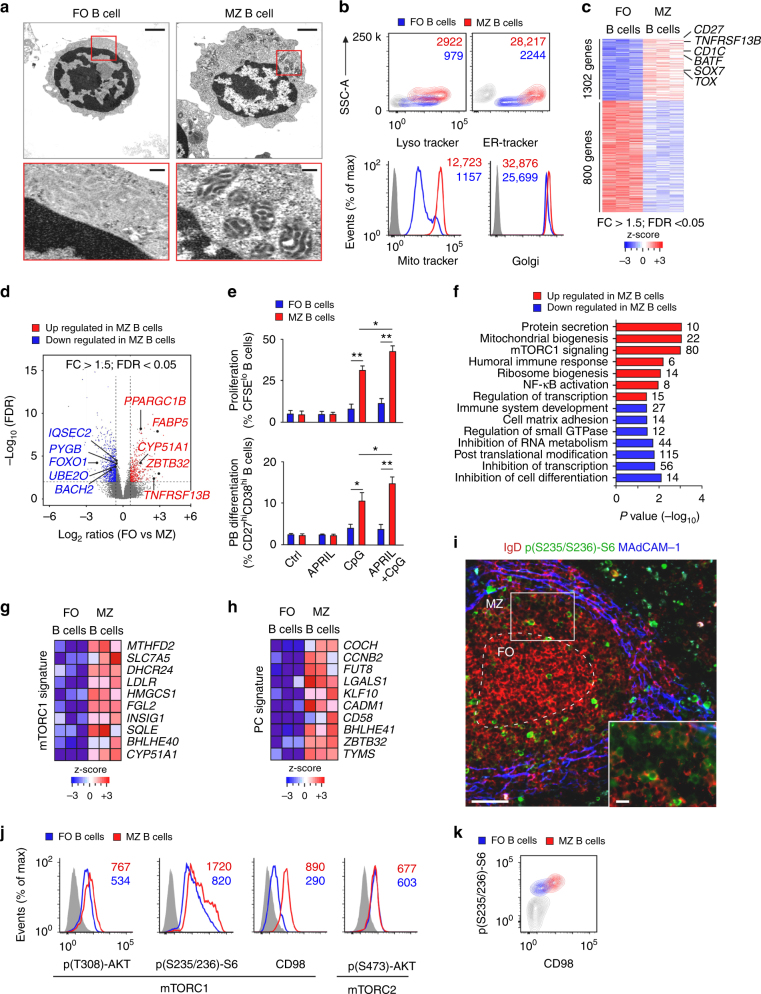
MZ B cells couple increased TACI expression with enhanced mTORC1 signaling. **a** Transmission electron microscopy of human splenic follicular (FO) and MZ B cells. Original magnification ×13,500 with 6× enlargement. Scale bars, 1 or 0.2 μm. **b** Flow cytometric analysis of LysoTracker, ER-Tracker, MitoTracker, and Golgi in human splenic FO and MZ B cells. SSC-A side scatter-area. **c**, **d** Transcriptome analysis of human splenic FO and MZ B cells. The heat map shows genes upregulated in FO (1302) and MZ (800) B cells and highlights a core MZ B-cell gene signature. The volcano plot summarizes comparably and differentially expressed genes. Red high expression, blue low expression, FC fold change, FDR false discovery rate. **e** FCM of human splenic FO and MZ B cells undergoing proliferation-induced CFSE dilution or CD19^+^CD27^hi^CD38^hi^ plasmablast differentiation following incubation with or without APRIL and/or CpG for 5 days. **f** Gene ontology analysis showing top seven biological processes upregulated (red) and downregulated (blue) by human MZ B cells compared to FO B cells. Numbers indicate genes. **g**, **h** Heat maps list top 10 genes similarly enriched or depleted in GSEA comparing genes expressed by human splenic MZ and FO B cells linked to mTORC1 activation (HALLMARK_MTORC1) (**g**) or PC differentiation (GSE22886) (**h**). **i** IFA of human spleen stained for IgD (red), p(S235/S236)-S6 (green), and MAdCAM-1 (blue). Scale bars, 100 μm (main image) or 10 μm (inset). **j**, **k** FCM of p(T308)-AKT, p(S473)-AKT, p(S235/S236)-S6, and CD98 in human FO and MZ B cells. Cells were gated as in Supplementary Fig. 11a (**b**, **j**, **k**). Data show one representative experiment of at least three with similar results (**a**, **b**, **i**–**k**), depict at least three biological replicates for each cell type (**c**, **d**, **f**-**h**), or summarize at least two experiments with at least two donors from each experimental group (**e**). Error bars, s.e.m.; *p < 0.05, **p < 0.01, ***p < 0.001 (two-tailed Student’s *t* test)

MZ B cells are poised to undergo plasmablast differentiation^27^, but this pre-activation state remains elusive, particularly in humans. We first integrated transmission electron microscopy with flow cytometry (FCM) to identify correlates of heightened activation in human splenic MZ B cells. Compared to naive splenic IgD^hi^CD27^−^ follicular B cells, splenic IgD^lo^CD27^+^ MZ B cells showed nuclei with increased loosely coiled chromatin, a hallmark of active gene transcription, and a larger cytoplasm with more abundant and hypertrophic mitochondria, lysosomes, ER, and Golgi apparatus (Fig. 1a, b and Supplementary Fig. 1a). These organelles coordinate anabolic metabolism with cell signaling and gene transcription via mTOR, a kinase also involved in immune activation^14,16^.

Next, we performed gene microarray and quantitative reverse transcriptase-polymerase chain reaction (qRT-PCR) assays to further elucidate the activation state of human MZ B cells and its possible relationship with mTOR. Compared to splenic follicular B cells and consistent with published data^28^, splenic MZ B cells showed a distinct transcriptome enriched in the differentiation molecules CD1c, CD27, *TOX*, and *SOX7* and in the activation molecule TACI (encoded by *TNFRSF13B*) (Fig. 1c, Supplementary Fig. 1b, and Supplementary Data 1), a MZ B-cell-stimulating receptor that cooperates with TLRs^4–6^. Accordingly, splenic MZ B cells also expressed more TLR9 (Supplementary Fig. 1b), a receptor for microbial CpG DNA (CpG) that increases TACI expression on MZ B cells^4,5^.

Further comparisons with splenic follicular B cells through volcano plot analysis and qRT-PCR validation assays showed that splenic MZ B cells expressed more mRNAs for BATF and *ZBTB32*, which facilitate B-cell CSR and proliferation but not memory duration^29,30^; less mRNAs for FOXO1 and BACH2, which inhibit B-cell proliferation and PC differentiation^22,31,32^; and less mRNA for *UBE2O* (Fig. 1c, d, Supplementary Fig. 1b, and Supplementary Data 1), which suppresses NF-κB induction by tumor necrosis factor receptor-associated factor 6 (TRAF6)^33^, an adapter shared by TACI and TLRs^4^. These signaling programs extensively intersect the mTOR pathway^22^, suggesting that mTOR contributes to the enhanced preparedness of MZ B cells toward expansion and plasmablast formation. Indeed, compared to splenic follicular B cells, human splenic MZ B cells underwent more proliferation and plasmablast differentiation in response to TACI and TLR9 ligation by APRIL and CpG, respectively (Fig. 1e).

We then used ingenuity pathway analysis and qRT-PCRs to identify mTOR gene networks. Compared to splenic follicular B cells, splenic MZ B cells expressed more gene products encoding mTORC1-linked regulators of protein and lipid metabolism (*PPARGC1B*; *CYP51A1*; *FABP5*; *FASN*; and *PPIB*) as well as apoptosis and cytoskeleton remodeling (*CFLAR* and *PFN1*), but fewer gene products encoding regulators of glycogenolysis and cell motility (*PYGB* and *IQSEC2*) (Supplementary Fig. 1b, c). Similarly, gene ontology analysis indicated that, compared to splenic follicular B cells, splenic MZ B cells expressed more gene products mediating mTORC1 signaling, protein secretion, and organelle biogenesis, but fewer gene products inhibiting RNA metabolism, transcription, and cell differentiation (Fig. 1f and Supplementary Data 1).

Consistent with these data, ingenuity’s upstream regulator analysis showed increased splenic MZ B-cell activation of mTOR as well as SREBF2 and SREBP cleavage-activating protein (Supplementary Fig. 1d), two mTORC1-induced lipid metabolism regulators^34^. Instead, rapamycin-insensitive companion of mTOR (RICTOR), a component of mTORC2^16^, was more inhibited in splenic MZ B cells (Supplementary Fig. 1d). These cells were also enriched in gene products linked to NF-κB induction (Fig. 1f) and showed increased activation of transcripts linked to NF-κB-inducing BCR, TLRs, and tumor necrosis factor (TNF)-like receptors (Supplementary Fig. 1d), which include TACI^2^. Thus, the pre-activated state of human splenic MZ B cells couples increased TACI expression with gene signatures reflecting enhanced mTOR as well as NF-κB activation.

### MZ B cells gene signature correlates with enhanced mTORC1 activity

Next, we studied mTORC1 activation in human splenic MZ B cells. Gene set enrichment analysis (GSEA) showed that, compared to splenic follicular B cells, splenic MZ B cells were enriched in mTORC1-related gene products regulating energy production (*CYP51A1* and *MTHFD2*), lipid metabolism (*DHCR24*, *LDR*, *HMGCS1*, *INSIG1*, and *SQLE*), protein metabolism (*SCL7A5*), and lymphocyte differentiation (*FGL2* and *BHLHE40*) (Fig. 1g, Supplementary Fig. 1e, and Supplementary Data 1). As shown by GSEA, this mTORC1 gene signature correlated with a PC gene signature comprising increased splenic MZ B-cell expression of gene products regulating cell adhesion (*COCH*, *CADM1*, and *CD58*), cell proliferation (*CCNB2* and *TYMS*), antibody glycosylation (*FUT8*), and PC differentiation and survival (*LGALS1*, *BHLHE41*, *ZBTB32*, and *KLF10*) (Fig. 1h, Supplementary Fig. 1f, and Supplementary Data 1). Similarly, FCM showed slightly increased PC-inducing proteins such as BLIMP-1 and UPR-related activating transcription factor-4 (ATF-4) in splenic MZ B cells (Supplementary Fig. 1g).

Consistent with their mTORC1 gene signature, IgD^lo^ B cells detected by immunofluorescence analysis (IFA) within splenic mucosal addressin and cell-adhesion molecule (MAdCAM-1)^+^ stromal MZ areas expressed S6 phosphorylated (p) at serine (S) 235 or 236 (Fig. 1i), a hallmark of mTORC1 activation^16^. Accordingly, FCM showed increased p(S235/236)-S6 and mTORC1-targeting AKT phosphorylated at threonine (T) 308 but comparable mTORC2-targeting p(S473)-AKT in MZ B cells compared to follicular B cells (Fig. 1i and Supplementary Fig. 1h). At the transcriptional level, splenic MZ and follicular B cells comparably expressed mRNAs for AKT, mTOR, RAPTOR, RICTOR, and the mTORC1/2 inhibitor DEP domain-containing mTOR-interacting protein (DEPTOR), but less tuberous sclerosis protein-1 (TSC1) (Supplementary Fig. 1i), which inhibits mTORC1 together with TSC2^16^. Finally, in splenic MZ but not follicular B cells, p(S235/236)-S6 phosphorylation was associated with upregulation of CD98 (Fig. 1k and Supplementary Fig. 1j), an mTORC1-induced amino acid transporter mediating T-cell-independent antibody production^35^. Thus, increased mTORC1 signaling in human splenic MZ B cells is associated with immunometabolic gene traits reflecting increased predisposition to PB differentiation.

### TACI stimulates mTORC1 transcription programs in MZ B cells

**Fig. 2:**
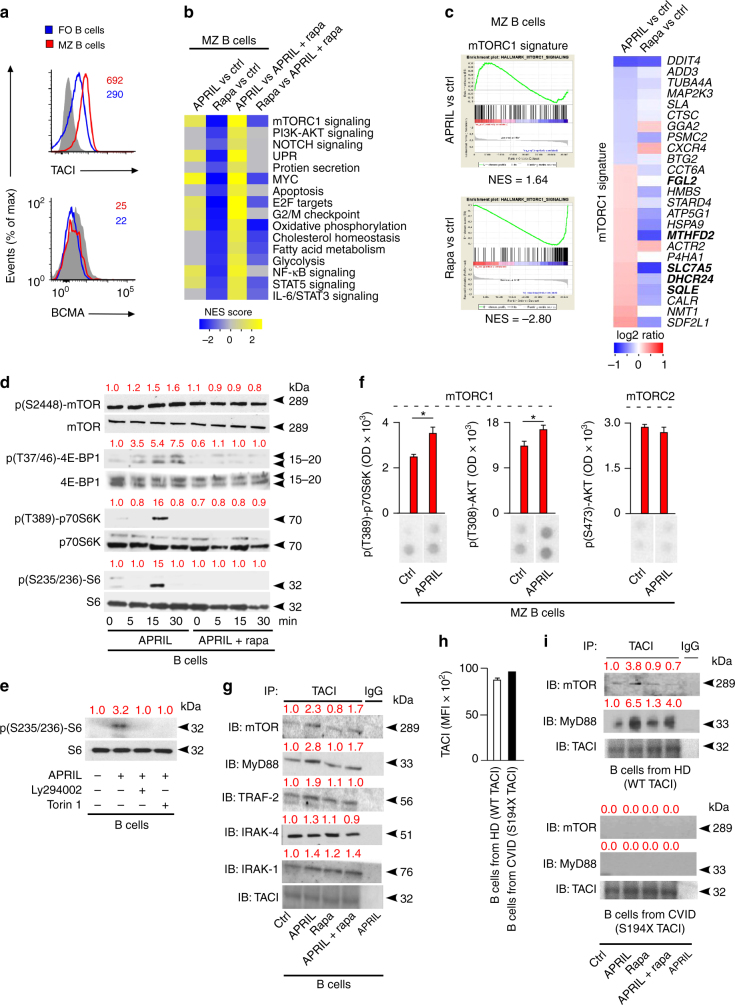
TACI activates mTORC1 signaling by recruiting mTOR. **a** FCM of TACI and BCMA on human splenic FO and MZ B cells. Cells were gated as in Supplementary Fig. 11a **b**. Heat map showing enrichment or depletion of coordinated gene sets identified by GSEA in the transcriptome from human MZ B cells cultured for 3 h with or without APRIL and/or rapamycin (rapa). Ctrl medium. NES indicates correlation between individual gene sets. Positive correlation, NES > 0 (yellow gradient); negative correlation, NES < 0 (blue gradient). **c** GSEA of the mTORC1 gene signature identified in **b** and heat map listing top 25 genes similarly enriched or depleted under different treatment conditions. Colors correspond to significant FC expression: red, high; blue, low. Bold indicates genes discussed in the text. **d** IB of total or p(S2448)-mTOR, p(37/46)-4E-BP1, p(T389)-70S6K, and p(S235/236)-S6 in human splenic IgD^+^ B cells incubated for 0, 5, 15, or 30 min with APRIL in the presence or absence of rapamycin. Red numbers indicate band intensity relative to total protein. **e** IB of total or p(S235/236)-S6 in human splenic IgD^+^ B cells incubated for 15 min with APRIL in the presence or absence of Ly294002 or Torin 1. **f** Phospho-proteome analysis of p(S235/236)-S6, p(T308)-AKT, and p(S473)-AKT from human splenic MZ B cells stimulated with APRIL for 15 min. Graphs show mean pixel density of dot blots. OD optical density. **g** IB of mTOR, MyD88, TRAF2, IRAK-4, IRAK-1, and TACI following IP with anti-TACI or irrelevant IgG of protein lysates from splenic IgD^+^ B cells incubated with or without APRIL and/or rapamycin for 15 min. Ctrl, medium alone. **h** FCM of WT and S194X TACI on lymphoblastoid B cells from a healthy donor (HD) or a common variable immunodeficiency (CVID) patient, respectively. **i** IB of mTOR, MyD88, and TACI following IP with anti-TACI or irrelevant IgG antibodies of protein lysates from B cells shown in **h** stimulated as in **g**. Data summarize one representative experiment with at least three biological replicates for each cell type (**a**–**c**, **f**, **h**) or show one experiment of at least three with similar results (**d**, **e**, **g**, **i**). Error bars, s.e.m.; *p < 0.05 (two-tailed Student’s *t* test)

In DCs, mTOR binds MyD88 to integrate TLR signaling^36^. Given that MyD88 binds TACI through a MyD88-binding site (MBS) distinct from the TIR domain of TLRs^4^, we reasoned that TACI could recruit mTOR to optimize the transcriptional activation of MZ B cells. As shown by FCM, human splenic MZ B cells expressed more TACI than splenic follicular B cells did, whereas both B-cell types lacked B-cell maturation antigen (BCMA) (Fig. 2a and Supplementary Fig. 2a), which also binds APRIL^2^. Thus, we used APRIL to specifically engage TACI. As shown by GSEA, splenic MZ B cells enhanced mTORC1-coordinated gene sets implicated in cell proliferation (MYC, G2M checkpoint, and E2F targets), activation (NF-κB and STAT5), metabolism (oxidative phosphorylation), and PC differentiation (UPR) upon incubation with APRIL for 3 h (Fig. 2b and Supplementary Table 1).

In contrast, splenic MZ B cells exposed to rapamycin downregulated these genes and those linked to PI3K-AKT signaling, apoptosis, metabolism (glycolysis, oxidative phosphorylation, cholesterol homeostasis, and fatty acid synthesis) and PC differentiation (protein synthesis, IL-6, and STAT5 signaling) (Fig. 2b). Compared to APRIL and rapamycin, APRIL alone upregulated all the above gene sets, which instead were suppressed when rapamycin was compared to its combination with APRIL (Fig. 2b). Of note, mTORC1-related *FGL2*, *MTHFD2*, *SLC7A5*, *DHCR24*, and *SQLE* were increased in APRIL-induced splenic MZ B cells (Fig. 2c) as they were in splenic MZ vs. follicular B-cell comparisons shown earlier (Fig. 1g). Thus, TACI recapitulates some of the metabolic programs underpinning the pre-activated state of human splenic MZ B cells, whereas rapamycin suppresses these programs, suggesting that TACI is functionally linked to mTORC1.

### TACI recruits mTOR and activates mTORC1 in human MZ B cells

Next, we performed immunoblotting (IB) and phospho-kinase arrays to validate mTORC1 activation by TACI. As early as 10–15 min upon incubation with APRIL, human splenic IgD^+^ B cells slightly upregulated p(S2448)-mTOR, but strongly induced canonical mTORC1 targets^16,37^, such as p(T37/46)-4E-BP1, an inhibitor of the ribosomal elongation factor eIF4E, p(T389)-p70S6 kinase, an activator of the ribosomal protein S6, and p(S235/236)-S6, an inducer of protein translation, while these inductive events were suppressed by rapamycin (Fig. 2d). Besides p(S235/236)-S6, APRIL upregulated mTORC1-inducing p(T308)-AKT but not mTORC2-inducing p(S473)-AKT and this upregulation decreased in the presence of the PI3K inhibitor Ly294002 and the mTOR inhibitor Torin 1 (Fig. 2e, f).

Given that both TACI and mTOR bind MyD88^4,36^, we verified the association of these proteins upon TACI ligation. As shown by immunoprecipitation (IP) and IB, splenic IgD^+^ B cells exposed to APRIL for 15 min increased TACI association with mTOR, MyD88, interleukin-1 receptor-activated kinase 1 (IRAK-1), IRAK-4, and TRAF2 (Fig. 2g), which binds TACI through a TRAF2-binding site (TBS) proximal to the MBS^4^. Of note, rapamycin attenuated all these APRIL-induced signaling events (Fig. 2g). Similar IP assays were performed in Epstein Barr virus (EBV)-transformed B cells from a healthy donor expressing wild-type (WT) TACI or a common variable immunodeficiency patient with an S194X substitution disrupting the cytoplasmic but not extracellular domain of TACI (Fig. 2h). We found that mTOR and MyD88 increased their association with WT but not mutant S194X TACI via an APRIL-induced mechanism abrogated by rapamycin (Fig. 2i). Thus, TACI couples PI3K-AKT-mediated mTORC1 activation with recruitment of mTOR and MyD88.

### TACI interacts with mTOR via NF-κB-inducing MyD88

**Fig. 3:**
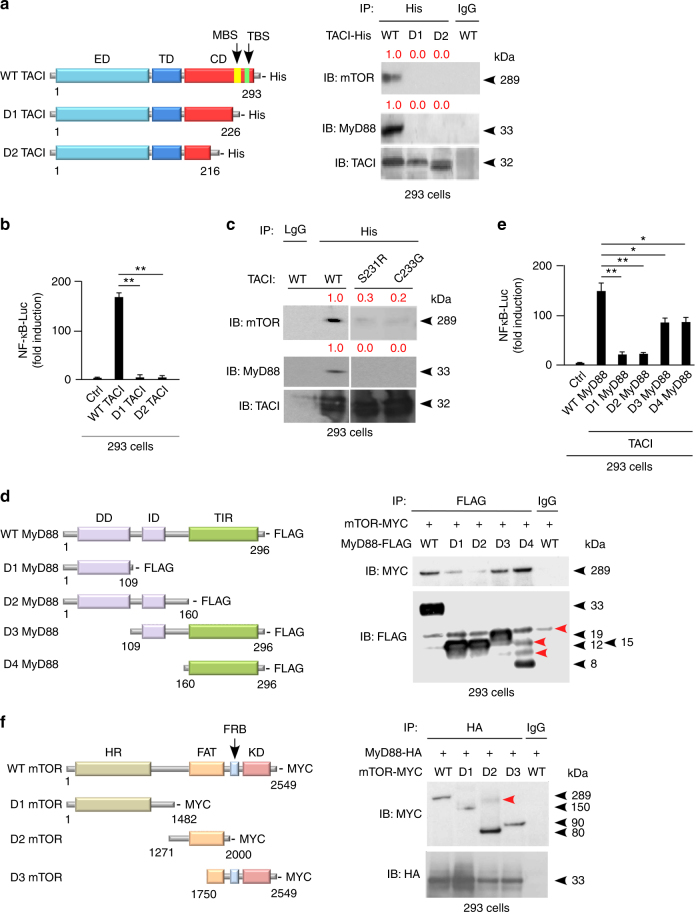
TACI uses a MyD88-binding site to attach the central domain of mTOR. **a** Left graphs: structure of His-tagged WT TACI or D1 and D2 TACI deletion mutants. Numbers indicate C-terminal and N-terminal residues. ED extracellular domain, TD transmembrane domain, CD cytoplasmic domain. Right gels: IB of mTOR, MyD88 and TACI following IP with anti-His or irrelevant IgG antibodies of protein lysates from transfected 293 cells expressing WT, D1, or D2 TACI. Red numbers indicate band intensity relative to TACI. **b** NF-κB luciferase (NF-κB-Luc) reporter assay of 293 cells transfected with WT or mutant TACI shown as in **a**. Ctrl, 293 cells transfected with an empty plasmid. **c** IB of mTOR, MyD88, and TACI following IP with anti-His or irrelevant IgG antibodies of protein lysates from transfected 293 cells expressing His-tagged WT TACI or mutant TACI proteins with site-targeted substitutions inhibiting MyD88 binding to the cytoplasmic MBS of TACI, including S231R TACI and C233G TACI. **d** Left graphs: structure of FLAG-tagged WT MyD88 or D1, D2, D3, and D4 MyD88 deletion mutants. Right gels: IB of MYC and FLAG following IP with anti-FLAG or irrelevant IgG antibodies of protein lysates from co-transfected 293 cells expressing FLAG-tagged WT, D1, D2, D3, and D4 MyD88 together with MYC-tagged mTOR. Red arrows indicate nonspecific bands. **e** NF-κB-Luc reporter assay of 293 cells co-transfected with WT TACI and WT or mutant MyD88 shown in **d** for 24 h. Ctrl, 293 cells transfected with an empty plasmid. **f** Left graphs: structure of MYC-tagged WT mTOR or D1, D2, and D3 mTOR deletion mutants. FAT stands for FRAP, ATM, TRRAP domain. Right gels: IB of MYC and HA following IP with anti-HA or irrelevant IgG antibodies of protein lysates from co-transfected 293 cells expressing MYC-tagged WT, D1, D2, and D3 mTOR together with HA-tagged MyD88. Data represent one of three experiments with similar results (**a**, **c**, **d**, **f**) or summarize three experiments (**b**, **e**). Error bars, s.e.m.; *p < 0.05, *p < 0.01 (two-tailed Student’s *t* test)

Next, we verified whether TACI interacts with mTOR via MyD88. First, 293 cells were transfected with histidine (His)-tagged WT TACI, or D1 TACI lacking both MBS and TBS, or D2 TACI lacking most of its cytoplasmic domain (Fig. 3a). IPs and luciferase reporter assays showed that WT TACI bound mTOR and MyD88 and induced NF-κB-driven gene transcription, whereas D1 and D2 TACI deletion mutants did not (Fig. 3a, b). Site-directed mutagenesis followed by IPs revealed that TACI harboring S231R or C233G substitutions, which disrupt MBS–MyD88 interaction^4^, abolished TACI association with both mTOR and MyD88 (Fig. 3c). By showing high-evolutionary conservation of MBS (Supplementary Fig. 2b), alignment studies confirmed the key role of MBS in TACI signaling.

We then asked how MyD88 interacts with mTOR. The MyD88 adapter uses a C-terminal Toll-interleukin-1 receptor (TIR) domain to interact with TLRs, an intermediary domain (ID) to interact with TACI, and an N-terminal death domain (DD) to interact with IRAK-1 and IRAK-4, which activate NF-κB^4^. Two hundred ninety-three cells were co-transfected with WT TACI, MYC-tagged WT mTOR, and FLAG-tagged WT MyD88, or D1 MyD88 lacking both ID and TIR domain, or D2 MyD88 lacking the TIR domain, or D3 MyD88 lacking the DD, or D4 MyD88 lacking both DD and ID (Fig. 3d). We found that WT, D3, and D4 but not D1 and D2 MyD88 interacted with mTOR and supported NF-κB-driven gene transcription in 293 cells, though D3 and D4 were less efficient than WT MyD88 (Fig. 3d, e).

Finally, we ascertained how MyD88 interacts with mTOR. The mTOR kinase encompasses an N-terminal HEAT repeats region (HR) binding RAPTOR or RICTOR, a central FAT (FRAP, ATM, and TRRAP) domain-binding DEPTOR, and a C-terminal kinase domain (KD) encompassing the FKBP12-rapamycin-binding motif^16,17^. 293 cells were co-transfected with hemoagglutinin (HA)-tagged WT MyD88 and MYC-tagged WT mTOR, or D1 mTOR lacking the FAT domain and KD, or D2 mTOR lacking the HR and KD, or D3 mTOR lacking the HR and most of the FAT domain (Fig. 3f). We found that WT, D2, or D3 mTOR deletion mutants interacted with MyD88 more effectively than D1 mTOR did (Fig. 3f). Thus, the MBS of TACI may recruit mTOR via an NF-κB-inducing mechanism involving binding of the TIR domain of MyD88 to the FAT domain of mTOR.

### TACI induces NF-κB activation via mTORC1 in MZ B cells

**Fig. 4:**
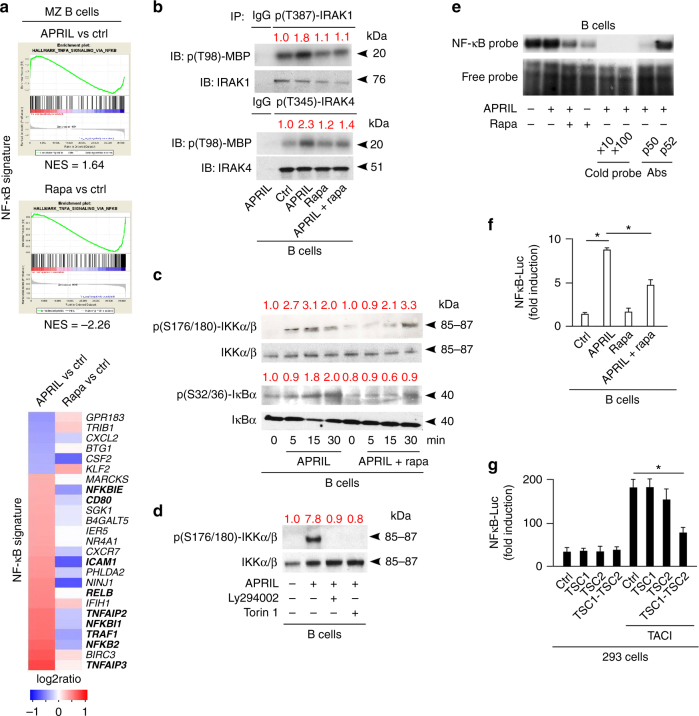
TACI activates the CSR-inducing transcription factor NF-κB through mTORC1. **a** GSEA showing enrichment or depletion of coordinated NF-κB gene sets from human splenic MZ B cells cultured for 3 h with or without APRIL or rapamycin (rapa). Ctrl, medium alone. NES indicates correlation between individual gene sets. Positive correlation, NES > 0; negative correlation, NES < 0. Heat map shows top 25 genes similarly enriched or depleted in MZ B cells exposed to different treatments. Colors correspond to significant FC expression: red (high); blue (low). Bold indicates genes discussed in the text. **b** IB of p(T98)-myelin basic protein (MBP), IRAK-1 and IRAK-4 following IP with anti-p(T387)-IRAK-1, p(T354)-IRAK-4, or irrelevant IgG of protein lysates from human 2E2 B cells cultured for 15 min with or without APRIL and/or rapamycin (rapa). Red numbers indicate band intensity relative to total proteins. **c** IB of total or p(S176/180)-IKKα/β and total or p(S32/36)-IκBα from human splenic IgD^+^ B cells incubated with APRIL in the presence or absence of rapamycin for 0, 5, 15, or 30 min. **d** IB of total or p(S176/180)-IKKα/β from human splenic IgD^+^ B cells incubated with or without APRIL in the presence or absence of Ly294002 or Torin 1 for 15 min. **e** EMSA of nuclear NF-κB bound to a radiolabeled κB DNA probe from human splenic IgD^+^ B cells incubated with APRIL in the presence or absence of rapamycin for 1 h (lanes 1–4). NF-κB–DNA interaction in the presence of increasing concentrations of a competing unlabeled cold probe (lanes 5–6) or blocking antibodies (Abs) to p50 and p52 NF-κB subunits (lanes 7–8) are also shown. **f** NF-κB-Luciferase (Luc) reporter assay of human EBV-transformed B cells incubated with APRIL in the presence or absence of rapamycin for 24 h. **g** NF-κB-Luc reporter assay of 293 cells expressing WT TACI with or without TSC1, TSC2, or both for 24 h. Ctrl, empty plasmid. Data summarize one representative experiment with at least three samples or biological replicates from each cell type (**a**, **f**, **g**), show one of at least three (**b**, **d**) or two (**e**) experiments with similar results. Error bars, s.e.m.; *p < 0.05 (two-tailed Student’s *t* test)

Given that mTOR binds TACI via the NF-κB-inducing adapter MyD88^4^, we wondered whether TACI requires mTOR to activate NF-κB. First, GSEA from human splenic MZ B cells exposed to APRIL for 3 h showed an enriched NF-κB-related gene signature that included NF-κB inhibitor α (IκBα) and IκBε (*NFKBI1* and *NFKBIE*), NF-κB p100 (*NFKB2*), CD80 and CD54 (*ICAM1*), TRAF1 and TNF-α-induced proteins 1 and 2 (*TNFAIP1* and *TNFAIP2*) (Fig. 4a). Conversely, splenic MZ B cells suppressed their constitutively increased NF-κB signature upon exposure to rapamycin (Fig. 4a). In this regard, FCM confirmed increased NF-κB p65 expression in splenic MZ B cells compared to follicular B cells (Supplementary Fig. 3a).

We then verified whether TACI activates IRAK-1 and IRAK-4 via mTOR. Activated IRAKs phosphorylate the catalytic α and β subunits of an IκBα kinase (IKK) complex that phosphorylates IκBα^38^. By eliciting IκBα degradation, this process releases p50-p65 NF-κB dimers from IκBα, thereby permitting their nuclear translocation. IP followed by kinase assays showed that human splenic IgD^+^ B cells exposed to APRIL for 15 min enhanced threonine 98 phosphorylation of myelin-binding protein, a substrate of p(T387)-IRAK-1 and p(T345)-IRAK-4, but this effect was attenuated by rapamycin (Fig. 4b). In addition, IBs showed that APRIL-induced IκBα degradation following p(S176/180)-IKKα/β and p(S32/36)-IκBα activation via a PI3K-(AKT)-mTOR-dependent mechanism attenuated or reversed by rapamycin, Torin 1, or Ly294002 (Fig. 4c, d). As shown by electrophoretic mobility shift assays (EMSAs), APRIL-triggered binding of p50-p65 NF-κB dimers to DNA, which was inhibited by rapamycin (Fig. 4e). Similarly, IFA showed APRIL-induced rapamycin-sensitive nuclear translocation of p50 and p65 in human splenic MZ B cells (Supplementary Fig. 3b).

Consistent with these findings, luciferase assays determined that rapamycin decreased NF-κB-driven gene transcription in EBV-transformed B cells from a healthy donor exposed to APRIL (Fig. 4f). Similarly, combined but not individual TSC1 and TSC2 overexpression reduced NF-κB-driven gene transcription in 293 cells stimulated through TACI (Fig. 4g). Thus, TACI may elicit PI3K-AKT-amplified TSC1–TSC2-restrained mTORC1 signals that activate NF-κB via an IKK pathway involving MyD88-interacting IRAK-1 and IRAK-4 kinases.

### TACI transcriptionally drives CSR via mTORC1 in MZ B cells

Given that TACI uses mTORC1 to enhance canonical NF-κB signaling and knowing that NF-κB is essential for CSR^39^, we verified whether TACI elicits CSR via mTORC1. CSR replaces IgM with IgG, IgA, or IgE through a mechanism guided by switch (S) regions positioned between an upstream intervening heavy chain (I_H_) promoter encompassing a non-coding I_H_ exon and a downstream constant µ (Cµ), Cγ, Cα or Cε gene^39^. Besides inducing the CSR-inducing enzyme activation-induced cytidine deaminase (AID), NF-κB-activating receptors such as TACI stimulate I_H_ promoters to initiate germline I_H_-S-C_H_ transcription and AID-mediated CSR^39^. This process removes the DNA between targeted S regions to generate extrachromosomal switch circles encoding Iµ-C_X_ transcripts, a hallmark of ongoing CSR^39^.

**Fig. 5:**
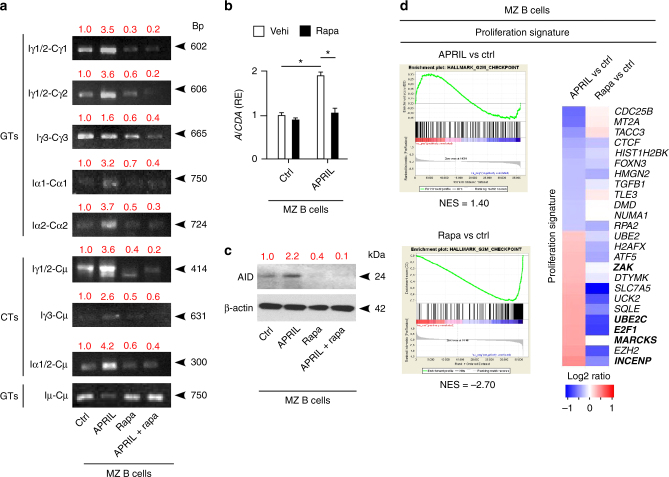
TACI triggers CSR by cooperating with TLR9 through mTORC1. **a** PCR analysis of Iγ1/2-Cγ1, Iγ1/2-Cγ2, Iγ3-Cγ3, Iα1-Cα1, Iα2-Cα2 and Iµ-Cµ germline transcripts (GTs) as well as Iγ1/2-Cµ, Iγ3-Cµ, Iα1/2-Cµ switch circle transcripts (SCTs) RT-PCR-amplified from human splenic MZ B cells incubated with APRIL in the presence or absence of rapamycin (rapa) for 3 days. Red numbers indicate band intensity relative to Iµ-Cµ bands. **b** qRT-PCR of mRNA for AID (*AICDA*) from human splenic MZ B cells incubated with APRIL in the presence of control vehicle (vehi) or rapamycin for 3 days. Results are normalized to β-actin mRNA and presented as relative expression (RE) compared to B cells incubated with medium alone (Ctrl). **c** IB of AID and β-actin from human splenic MZ B cells treated as in **a**. Red numbers indicate band intensity relative to β-actin. **d** GSEA showing enrichment or depletion of coordinated proliferation-related gene sets from human splenic MZ B cells cultured for 3 h with or without APRIL or rapamycin. Ctrl, medium alone. NES indicates correlation between individual gene sets. Positive correlation, NES > 0; negative correlation, NES < 0. Heat map showing top 25 genes similarly enriched or depleted in MZ B cells exposed to different treatments. Colors correspond to significant FC expression: red, high expression; blue, low expression. Bold indicates gene products discussed in the text. Data represent one of three experiments with similar results (**a**, **c**), summarize three experiments (**b**) or correspond to one experiment with three biological replicates (**d**). Error bars, s.e.m.; *p < 0.05, **p < 0.01 (two-tailed Student’s *t* test)

Consistent with their pre-activated state involving constitutive NF-κB activation, untreated human splenic MZ B cells expressed some germline Iγ2-Cγ2 and Iγ3-Cγ3 transcripts, switch circle Iγ1/2-Cµ, Iγ3-Cµ and Iα1/2-Cµ transcripts, AID-encoding *AICDA* transcripts and AID protein (Fig. 5a–c). In addition to upregulating these transcripts and AID, MZ B cells induced Iγ1-Cγ1 and Iα1-Cα1 following incubation with APRIL for 3 days, but addition of rapamycin abolished or mitigated both APRIL-induced as well as constitutive CSR-related events without affecting Iµ-Cµ transcripts (Fig. 5a–c).

NF-κB cooperates with AP-1, STAT, and CREB transcription factors to elicit proliferation in addition to CSR^39^. As shown by phospho-kinase arrays, incubation of splenic MZ B cells with APRIL for 15 min-activated AP-1-inducing p38 mitogen-activated protein kinase (MAPK), STAT5 and CREB (Supplementary Fig. 3c). Similarly, GSEA showed that APRIL increased splenic MZ B-cell expression of coordinated gene sets encoding proliferation-related proteins intersecting AP-1, STAT, and CREB pathways, including *ZAK*, *UBE2C*, *E2F1*, *MARCKS*, and *INCENP* (Fig. 5d). Conversely, rapamycin inhibited this gene set in unstimulated splenic MZ B cells presumably primed by APRIL in vivo (Fig. 5d). Thus, besides enhancing CSR, TACI signaling via mTORC1 may transcriptionally lower the activation threshold of MZ B cells to facilitate their expansion into class-switched plasmablasts.

### TACI and TLRs cooperatively activate MZ B cells via mTORC1

**Fig. 6:**
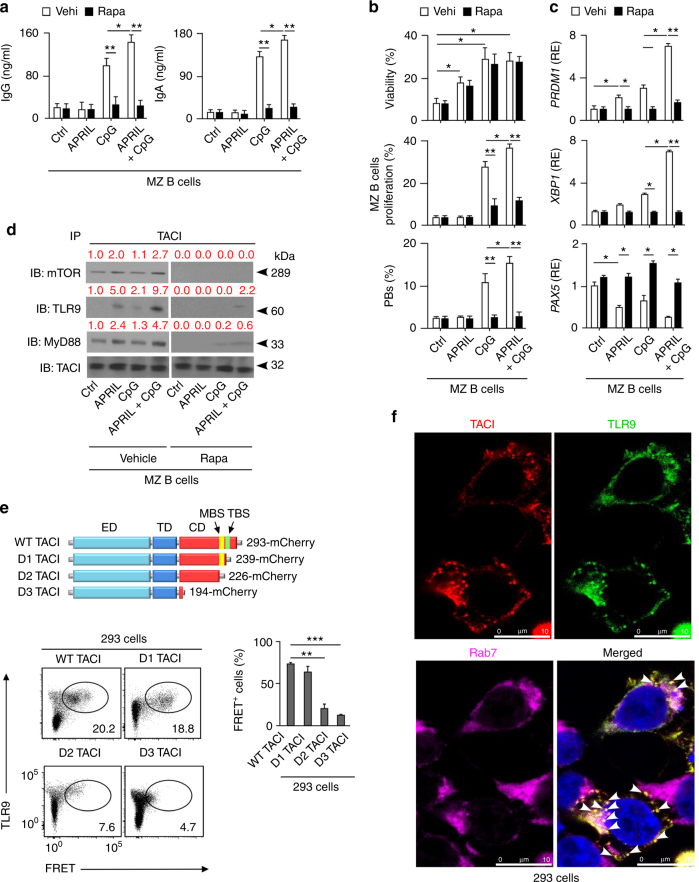
TACI interacts with active TLR9 and MyD88 through mTORC1. **a** ELISA of total IgG and IgA from human splenic MZ B cells cultured with or without APRIL and/or CpG and in the presence of control vehicle (vehi) or rapamycin (rapa) for 5 days. Ctrl, medium alone. **b** Frequency of viable cells, proliferated CFSE^lo^ cells and CD19^+^CD27^hi^CD38^hi^ plasmablasts after culturing human splenic MZ B cells as in **a**. Proliferation data derive from experiments identical to those in Fig. 1e, which includes comparisons between MZ and follicular B cells with no exposure to rapamycin. **c** qRT-PCRs of mRNAs for BLIMP-1 (*PRDM1*), XBP-1 (*XBP1*), and PAX5 (*PAX5*) from human splenic MZ B cells cultured for 72 h as in **a**. Results are normalized to β-actin mRNA and presented as relative expression (RE) compared to B cells incubated with medium alone (ctrl). **d** IB of mTOR, TLR9, MyD88, and TACI following IP with an anti-TACI antibody of protein lysates from splenic IgD^+^ B cells cultured for 60 min with or without APRIL and/or CpG in the presence of control vehicle or rapamycin. Ctrl, medium alone. Red numbers indicate band intensity relative to TACI bands. **e** Top schematics: structure of mCherry-tagged WT TACI or D1, D2, and D3 TACI deletion mutants (acceptors) used in FRET assays. Bottom graphs: FCM of FRET signal in co-transfected 293 cells expressing donor TLR9-eYFP together with mCherry-tagged WT or mutant acceptor TACI proteins as well as frequency of FRET^+^ 293 cells (**f**) IFA of 293 cells stained for transfected TACI (red) and TLR9 (green) as well as endogenous endosomal Rab7 (magenta). Bar, 10 μm. White arrows indicate endosomal TACI-TLR9 co-localization. Data summarize two experiments with at least two donors in each experimental group (**a**, **c**, **e**), show one representative experiment of at least three with similar results (**d**, **f**), or correspond to three biological replicates for each cell type (**b**). Error bars, s.e.m.; *p < 0.05, **p < 0.01, ***p < 0.001 (two-tailed Student’s *t* test)

TACI requires co-signals from TLRs, such as CpG-binding TLR9, to elicit MZ B-cell expansion and PB differentiation^4,5,26^. As shown by enzyme-like immunosorbent assays (ELISAs), FCM and carboxyfluorescein diacetate succinimidyl ester dilution assays, human splenic MZ B cells increased IgG and IgA secretion, survival, proliferation, and CD19^+^CD27^hi^CD38^hi^ PB differentiation upon 5-day exposure to APRIL and CpG but not APRIL alone, which only enhanced MZ B-cell survival (Fig. 6a, b and Supplementary Fig. 4a–c). These effects involved an additive effect of APRIL over CpG alone, correlated with detection of IgM^+^Ki-67^+^ plasmablasts in splenic MAdCAM-1^+^ MZ areas, and showed a dramatic reduction upon addition of rapamycin (Fig. 6a, b and Supplementary Fig. 4d). Yet, rapamycin spared splenic MZ B-cell survival (Fig. 6b). As shown by qRT-PCRs, rapamycin also inhibited the upregulation of PB-inducing BLIMP-1 and XBP-1, but reversed the downregulation of PB-suppressing PAX5 in MZ B cells exposed to APRIL and CpG (Fig. 6c). Next, we ascertained whether very low concentrations of rapamycin could specifically inhibit the induction of CSR by TACI and TLR9 ligands without impairing splenic MZ B-cell proliferation. We found that 0.1 or 0.01 nM rapamycin did not inhibit splenic MZ B-cell proliferation, plasmablast differentiation, BLIMP-1 induction, PAX5 downregulation, and IgM secretion, but sufficed to inhibit IgG or IgA secretion (Supplementary Fig. 5a–d). We then verified whether rapamycin could also impair splenic follicular B-cell responses to CD40L. In agreement with recently published studies^24^, we found that rapamycin inhibited both plasma cell differentiation and proliferation of splenic follicular B cells exposed to CD40L supplemented with IL-21 and IL-10 (Supplementary Fig. 5e, f). Altogether, these data indicate that mTOR is essential for the activation of splenic MZ and follicular B cells by T-cell-independent or T-cell-dependent signals, respectively.

Given that both TACI and TLR9 recruit mTOR and MyD88^4,36^, we wondered whether mTOR and MyD88 facilitate TACI interaction with TLR9, an endosomal receptor that initiates signaling upon CpG-induced cleavage of its ectodomain^40^. Indeed, similar to other TNF-like receptors, TACI may traffic into endosomes upon ligation-induced internalization^41^. IP followed by IB showed that splenic IgD^+^ B cells induced robust TACI association with cleaved TLR9 as well as MyD88 and mTOR upon exposure to APRIL and CpG for 1 h (Fig. 6d). Though more modestly, this association also occurred in response to CpG or APRIL alone, but became undetectable upon addition of rapamycin under all culture conditions (Fig. 6d).

TACI interaction with TLR9 was further suggested by fluorescence resonance energy transfer (FRET) assays in 293 cells co-transfected with donor-enhanced yellow fluorescent protein (eYFP)-labeled TLR9 and acceptor mCherry-labeled WT, D1 (lacking TBS but not MBS), D2 (lacking MBS and TBS), or D3 (lacking the cytoplasmic domain) TACI proteins. As shown by FCM, WT and D1 TACI-induced significantly more FRET signal than D2 or D3 TACI did (Fig. 6e). Accordingly, IFA showed co-localization of TACI and TLR9 in Rab7-expressing endosomes from co-transfected 293 cells (Fig. 6f). Thus, TACI and TLR9 may cooperatively activate human splenic MZ B cells through transcriptionally regulated mTORC1-induced mechanisms possibly entailing TACI interaction with TLR9 via MyD88 and mTOR.

### TACI requires MZ B-cell-intrinsic mTOR to induce IgG

**Fig. 7:**
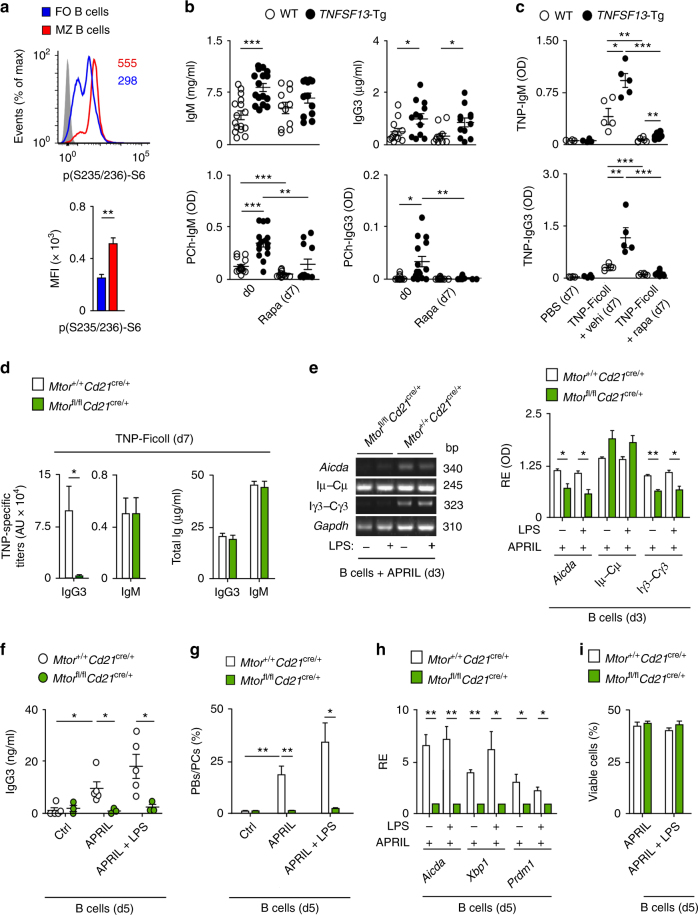
TACI requires mTOR to induce CSR in mouse MZ B cells. **a** FCM of p(S235/236)-S6 in splenic follicular and MZ B cells. Numbers in top panel indicate mean fluorescence intensity (MFI), whereas results from different experiments are summarized in bottom graph. Cells were gated as in Supplementary Fig. 11b. **b** ELISA of serum total and PCh-reactive IgM and IgG3 from WT and *TNFSF13*-Tg mice before (d0) and after (d7) daily i.p. treatment with rapamycin. **c** ELISA of serum TNP-specific IgM and IgG3 (d7) from WT and *TNFSF13*-Tg mice following i.p. immunization with TNP-Ficoll supplemented with daily i.p. injection of control vehicle or rapamycin. **d** ELISA of serum TNP-specific or total IgM and IgG3 (d7) from *Mtor* ^*+/+*^ *Cd21* ^*cre/+*^ or *Mtor* ^*fl/fl*^ *Cd21* ^*cre/+*^ mice following i.p. immunization with TNP-Ficoll. **e** RT-PCRs of mRNAs for AID (*Aicda*), Iµ-Cµ, Iγ3-Cγ3, and GAPDH (*Gapdh*) in splenic resting B cells from *Mtor* ^*fl/fl*^ *Cd21* ^*cre/+*^ or *Mtor* ^*+/+*^ *Cd21* ^*cre/+*^ mice treated for 3 days with APRIL in the presence or absence of LPS. Results are normalized to mRNA for *Gapdh* and quantified as relative expression (RE) compared to B cells from control *Mtor* ^*+/+*^ *Cd21* ^*cre/+*^ mice. **f** ELISA of total IgG3 secreted by splenic resting CD43^−^ B cells from *Mtor* ^*+/+*^ *Cd21* ^*cre/+*^ or *Mtor* ^*fl/fl*^ *Cd21* ^*cre/+*^ mice treated for 5 days with or without APRIL combined or not with LPS. **g** FCM of viable B220^−^CD138^+^ plasmablasts and PCs generated by splenic resting CD43^−^ B cells from *Mtor* ^*+/+*^ *Cd21* ^*cre/+*^ or *Mtor* ^*fl/fl*^ *Cd21* ^*cre/+*^ mice following stimulation as in **f**. **h** qRT-PCRs of mRNAs for AID (*Aicda*), XBP-1 (*Xbp1*), and BLIMP-1 (*Prdm1*) in splenic resting CD43^−^ B cells from *Mtor* ^*+/+*^ *Cd21* ^*cre/+*^ or *Mtor* ^*fl/fl*^ *Cd21* ^*cre/+*^ treated as in **f**. Results are normalized to mRNA for *Gapdh* and presented as RE compared to B cells from control *Mtor* ^*fl/fl*^ *Cd21* ^*cre/+*^ mice. **i** Frequency of viable splenic resting CD43^−^ B cells cultured as in **f**. Data summarize at least two experiments with at least two animals in each experimental group (**a**–**g**) and show one representative replicate (**e**, **g**). Error bars, s.e.m.; *p < 0.05, **p < 0.01, ***p < 0.001 (two-tailed Student’s *t* test)

Finally, we determined the impact of mTOR in mouse TACI-induced antibody responses. Similar to human MZ B cells, splenic B220^+^CD21^hi^CD23^lo^ MZ B cells from WT C57BL/6 mice expressed more TACI and mTORC1-induced p(S235/236)-S6 but not BCMA compared to splenic B220^+^CD21^+^CD23^hi^ follicular B cells (Fig. 7a and Supplementary Fig. 6a). We then verified whether mTORC1 inhibition by rapamycin affected homeostatic MZ B-cell responses to phosphorylcholine (PCh), a T-cell-independent antigen linked to microbial polysaccharides. ELISAs showed that WT mice treated for 7 days with rapamycin decreased serum PCh-specific but not total IgM and IgG3 (Fig. 7b). Rapamycin induced a similar effect in mice expressing a human APRIL-encoding *TNFSF13* transgene (*TNFSF13*-Tg) that strongly activates TACI^42^. Indeed, compared to controls, *TNFSF13*-Tg mice showed increased PCh-specific IgM and IgG3 (Fig. 7b). When administered following intraperitoneal immunization with TNP-Ficoll, which is a TACI-activating T-cell-independent antigen mimicking microbial polysaccharides^6^, rapamycin treatment decreased TNP-specific IgM and IgG3 induction but not splenic B-cell survival in both WT and *TNFSF13*-Tg mice (Fig. 7b and Supplementary Fig. 6b, c). As shown by IFA, rapamycin also reduced splenic p(S235/236)-S6^+^IgM^+^ plasmablasts in immunized *TNFSF13*-Tg mice (Supplementary Fig. 6d). In agreement with earlier human in vitro data, rapamycin also inhibited splenic follicular B-cell responses to a TNP-haptenated T-cell-dependent protein antigen, including TNP-specific IgM and IgG production, GC formation, and TNP-specific PB induction (Supplementary Fig. 7). We then verified whether mTOR signaling is impaired in MZ B cells lacking TACI. Flow cytometry and RT-PCRs demonstrated comparable expression of p-S6 and immune activation (*Bach2* and *Zbtb32*) or mTORC1-related (*Tsc1*, *Slc7a5*, and *Atf4*) transcripts in splenic MZ B cells from WT and *Tnfrsf13b* ^*−/−*^ mice (Supplementary Fig. 8a, b). These findings may reflect overstimulation of MZ B cells from *Tnfrsf13b* ^*−/−*^ mice by mTOR-inducing signals emanating from BAFF-R. Consistent with this possibility, BAFF serum levels were higher in *Tnfrsf13b* ^*−/−*^ mice compared to WT mice (Supplementary Fig. 8c). Next, we verified whether IgG responses to a TACI-dependent T-cell-independent antigen required MZ B-cell-intrinsic signals from mTOR. Compared to control *Mtor* ^*+/+*^ *Cd21* ^*cre/+*^ mice, *Mtor* ^*fl/fl*^ *Cd21* ^*cre/+*^ mice specifically lacking mTOR in B220^+^ B cells but not B220^−^ non-B cells showed conserved splenic follicular and MZ B cells as well as serum IgM and IgG3 (Supplementary Fig. 9a–d). However, TNP-specific IgG3 was decreased in *Mtor* ^*fl/fl*^ *Cd21* ^*cre/+*^ mice immunized for 7 days with TNP-Ficoll, whereas total IgM and IgG3 were not affected (Fig. 7d). Of note, TNP-specific IgM was normal in immunized *Mtor* ^*fl/fl*^ *Cd21* ^*cre/+*^ mice (Fig. 7d), likely due to conserved mTOR expression by splenic and peritoneal B220^+^CD21^−^CD23^−^CD43^+^ B-1 cells.

Finally, we elucidated the role of B-cell-intrinsic mTOR in antibody production induced by TACI ligation. Compared to controls, resting splenic CD43^−^ B cells from *Mtor* ^*fl/fl*^ *Cd21* ^*cre/+*^ mice showed comparable Iµ-Cµ and survival, but induced less Iγ3-Cγ3, *Aicda*, IgG3, B220^−^CD138^+^ plasmablasts, *Prdm1* (encoding BLIMP-1) and *Xbp1* upon 3- or 5-day exposure to APRIL alone or combined with a suboptimal concentration of lipopolysaccharide (LPS) (Fig. 7e–i and Supplementary Fig. 9e), a TLR4 ligand that strongly stimulates mouse B cells. Thus, TACI may recruit mTOR via MyD88 to integrate TLR-amplified transcription programs implicated in mouse MZ B-cell responses to T-cell-independent antigens (Supplementary Fig. 10).

## Discussion

Here we show that elevated TACI expression correlated with increased mTOR activation in pre-activated MZ B cells. By interacting with TACI via MyD88, mTOR linked proximal TACI signaling events with distal metabolic and immune transcription programs that facilitated the differentiation of MZ B cells into IgG class-switched plasmablasts. These programs encompassed NF-κB and their interruption by rapamycin or MZ B-cell-conditional mTOR deficiency impaired IgG responses to T-cell-independent antigens.

The kinase mTOR functions as a rheostat that integrates immunity with metabolism^14,15^. This is exemplified by studies showing that T cells require the large neutral amino acid transporter SLC7A5 to undergo clonal expansion in response to antigen^43^. By optimizing the intracellular supply of leucine, SLC7A5 induces mTORC1 and MYC signals that coordinate T-cell metabolism with proliferation^43^. In B cells, SLC7A5 belongs to a CD98 protein complex that promotes T-cell-independent antibody production by interacting with integrins^35^. In agreement with these findings from mouse B cells, we found that human splenic MZ B cells featured a pre-activation state characterized by increased CD98 expression and mTORC1 activation compared to splenic follicular B cells.

Besides promoting metabolic remodeling, mTORC1 may transcriptionally sustain the increased propensity of splenic MZ B cells to undergo proliferation and PB differentiation. Indeed, compared to human splenic follicular B cells, human splenic MZ B cells coupled enhanced AKT and mTORC1 activation with reduced expression of FOXO1 and BACH2, two transcriptional inhibitors of B-cell proliferation and PC differentiation suppressed by AKT-induced mTORC1 or mTORC2^22,31,32^. Conversely, human splenic MZ B cells expressed more MYC, BLIMP-1, ZBTB32, and BHLHE41, which transcriptionally induce proliferation and PB differentiation via gene networks partly controlled by AKT and mTORC1^12,19,20,22,30,43–46^.

Consistent with a key role of mTORC1, human splenic MZ B cells contained more mTORC1-regulated mitochondria, ER, lysosomes, and Golgi apparatus and featured an mTORC1 gene signature linked to increased organelle biogenesis as well as lipid and protein synthesis compared to human splenic follicular B cells. This metabolic gene signature paralleled phenotypic, transcriptional, and functional properties reflecting increased human splenic MZ B-cell responsiveness to T-cell-independent stimuli, including enhanced proliferation and PB differentiation in response to APRIL and CpG, which correlated with increased TACI and TLR9 expression. While rapamycin inhibited both metabolic and immune activation signatures, TACI ligation increased them, suggesting that the pre-activated state of human splenic MZ B cells involves TACI cooperation with mTORC1. Indeed, TACI ligation co-enhanced mTORC1 and NF-κB gene signatures and activated NF-κB via mTORC1. These findings in splenic MZ B cells from TACI-sufficient humans were seemingly contradicted by the detection of conserved mTORC1 signaling in splenic MZ B cells from TACI-deficient mice. However, it is important to bear in mind that peripheral B cells from TACI-deficient mice undergo expansion as a result of increased B-cell survival signals from BAFF-R, an mTOR-activating BAFF receptor alternative to TACI^47–49^. By showing increased BAFF production in TACI-deficient mice, our data suggest that the conserved mTORC1 signaling observed in MZ B cells from TACI-deficient mice stems from enhanced BAFF-R activity.

In general, immune receptors activate mTOR through the PI3K-AKT axis^14,15^. However, additional evidence shows that mTOR activation further involves mTOR interaction with adapter proteins linked to immune receptors. For instance, plasmacytoid DCs integrate interferon-inducing IRF7 signals from TLR9 by promoting mTOR interaction with MyD88^36,50^. Similarly, MZ B cells integrate antibody-inducing NF-κB signals from TACI by recruiting MyD88 in addition to TRAFs^4^. This implies that mTOR association with MyD88 could contribute to the activation of NF-κB by TACI. Accordingly, we found that TACI-induced NF-κB through an MBS-dependent rapamycin-sensitive pathway involving MyD88 association with mTOR via TIR and FAT domains, respectively.

In agreement with the intertwined nature of TACI-induced mTORC1 and NF-κB signals, rapamycin attenuated not only the phosphorylation of 4E-BP1, p70S6K, and S6 downstream of mTORC1, but also the activation of IRAK-1, IRAK-4, and IKK as well as the nuclear translocation of NF-κB in human B cells or 293 cells induced by TACI. Similar inhibitory effects emerged upon inhibition of PI3K, blockade of the mTORC1 catalytic site by Torin 1, or overexpression of the inhibitory TSC1–TSC2 complex, indicating that TACI augments NF-κB activation via PI3K-AKT-dependent TSC-restrained signals from mTORC1.

In T cells and DCs, mTORC1 regulates activation and fate decisions by regulating the activation of MYC, SREBPs, IRFs, STATs, NF-κB, FOXP3, T-bet, GATA3, and RORγt proteins^18^. In follicular B cells, mTORC1 promotes IgG responses^19,20^, but it remains unclear how antibody-inducing receptors are mechanistically linked to mTOR. By showing that mTORC1 links proximal TACI signaling events with distal NF-κB transcription programs via MyD88, our data suggest that the MyD88-mTOR signaling node is central to the activation of human splenic MZ B cells as it is to the activation of mouse T cells and DCs^36,50,51^. The precise mechanism whereby TACI–MyD88 interaction activates mTORC1 remains unclear, but binding of MyD88 to the FAT domain of mTOR could displace DEPTOR, an mTORC1 inhibitory protein^16^. Besides modulating TACI signaling through its kinase activity, mTORC1 may function as a scaffolding protein optimizing TACI recruitment of MyD88, IRAKs, and TRAFs.

In addition to NF-κB, TACI ligation activated AP-1-inducing p38 MAPK, STAT5, and CREB transcription factors, which cooperate with NF-κB to promote B-cell proliferation, CSR, and differentiation^7^. Moreover, TACI ligation-induced binary PB-inducing switch gene networks that coupled BLIMP-1 and XBP-1 induction with PAX5 suppression through a TLR9-amplified pathway generating IgG class-switched plasmablasts. Consistent with evidence showing that NF-κB, STATs, CREB, and BLIMP-1 intersect signals from mTORC1^12,14,15^, rapamycin abrogated all these transcriptional and functional events, but increased PAX5 expression and did not affect human splenic MZ B-cell survival. Of note, low concentrations of rapamycin could inhibit T-cell-independent CSR without affecting human splenic MZ B-cell proliferation and plasmablast differentiation, including IgM secretion. This finding indicates that mTORC1 specifically regulates CSR-inducing signaling pathways emanating from TACI and TLR9. In general, mTORC1 may coordinate multiple MZ B-cell-stimulating transcription programs by integrating TACI, TLR9, and perhaps even BCR signals via MyD88. Accordingly, TLRs and BCR cooperatively bind T-cell-independent antigens and loss of MyD88 impairs human MZ but not follicular B-cell responses^7,52,53^.

The cooperation of surface TACI with TLR9 could involve TACI internalization by endosomes, a ligation-induced event that regulates signaling from TNF-like receptors similar to TACI^41^. Consistent with this possibility, TACI associated with cleaved endosomal TLR9 as well as MyD88 and mTOR upon stimulation of human B cells with APRIL and CpG. This association was abrogated by rapamycin or loss of the MBS of TACI, suggesting that MyD88 from internalized TACI co-opts mTORC1 to enhance CpG-induced activation of endolysosomal proteases required for TLR9 cleavage and signaling^40^. This interpretation could explain why some APRIL-engaged TACI interacts with endosomal TLR9 even in the absence of CpG^54^.

Similar to human splenic MZ B cells, mouse splenic MZ B cells showed increased TACI expression and mTORC1 activation compared to splenic follicular B cells. Accordingly, homeostatic or post-immune IgG3 and IgM responses to T-cell-independent antigens, such as PCh and TNP-Ficoll, increased in APRIL-overexpressing *TNFSF13*-Tg mice compared to WT controls. Blockade of mTORC1 by rapamycin reduced TNP-specific IgG3 responses as well as mTORC1 activation in antigen-induced plasmablasts without affecting total IgG3 and IgM production and splenic B-cell survival. As previously published by others^20,21,24,55^, inhibition of mTORC1 by rapamycin blocked not only T-cell-independent antibody responses to a polysaccharide antigen, but also T-cell-dependent antibody responses to a protein antigen. Finally, TNP-specific but not total IgG3 responses decreased in *Mtor* ^*fl/fl*^ *Cd21* ^*cre/+*^ mice specifically lacking mTOR in mature CD21^+^ B cells. This deficiency neither affected splenic MZ and follicular B-cell survival nor TNP-specific IgM responses, which may derive from mTOR-sufficient splenic and peritoneal CD21^−^ B-1 cells.

Similar to rapamycin-treated human splenic MZ B cells, resting mouse splenic B cells lacking mTOR showed impaired IgM-to-IgG3 CSR, PB differentiation, and IgG3 secretion when exposed to APRIL alone or combined with LPS, a TACI-inducing TLR4 ligand^5,26^. These inhibitory effects correlated with impaired induction of genes encoding AID, BLIMP-1, and XBP-1, whereas B-cell survival was unaffected. In summary, our human and mouse data indicate that mTORC1 drives innate-like MZ B-cell responses by linking proximal TACI signaling events with distal transcription programs through a TLR-amplified mechanism involving MyD88. Given the involvement of both TACI and MyD88 in autoantibody production^25,26,54^, these findings lend support to the use of mTORC1 inhibitors in autoimmune disorders.

## Methods

### Donors

Human splenocytes were obtained from fresh tissue samples as reported in published studies^56^. Briefly, mononuclear cells were isolated from histologically normal spleens from deceased organ donors or trauma patients without clinical signs of infection or inflammation that had undergone splenectomy. The use of blood and tissues was approved by the Ethical Committee for Clinical Investigation of the Institut Hospital del Mar d’Investigacions Mèdiques (CEIC-IMIM 2011/4494/I) and was mediated by the European Research Council (ERC-2011-ADG-20110310). Tissue samples were collected by the Department of Immunology of Hospital del Clínic (Barcelona, Spain). EBV-transformed B-cell lines were obtained as reported^57^.

### Mice

WT C57BL/6 mice were from Charles River Laboratories (Saint-Aubin-lès-Elbeuf, France), whereas *TNFSF13*-Tg (*Lck-APRIL Tg*), *Mtor* ^*fl/fl*^ *Cd21* ^*cre/+*^, and *Mtor* ^*+/+*^ *Cd21* ^*cre/+*^ mice were generated as reported previously^20,42,58^. To generate B-cell-conditional mTor knockout mice, *Mtor* ^*fl/*fl^ mice generated as published previously^2^ were crossed with *Cd21-cre* mice (Jackson Lab). The resulting *Mtor* ^*fl/fl*^ *Cd21* ^*cre/+*^ progeny specifically deleted the *Mtor* allele in B cells, whereas control *Mtor* ^*+/+*^ *Cd21* ^*cre/+*^ mice did not. *Mtor* ^*fl/fl*^ *Cd21* ^*cre/+*^ and *Mtor* ^*+/+*^ *Cd21* ^*cre/+*^ alleles were PCR identified using forward 5′-CTCACTGCTGTGCTCTATGACCTGAG-3′ and reverse 5′-TCTGGATGAGCATCTTGCGCAG-3′ primers, which yielded 660-bp and 480-bp fragments of exon 12 from the *Mtor* gene in WT and *Mtor* ^*fl/fl*^ *Cd21* ^*cre/+*^ mice, respectively. TACI-deficient mice (*Tnfrsf13b* ^*−/−*^) were kindly provided by Richard J. Bram (Department of Pediatric and Adolescent Medicine, Mayo Clinic, Rochester, MN, USA) and housed and bred at Icahn School of Medicine at Mount Sinai. All mice were maintained in specific pathogen-free conditions and both male and female were used at 8–14 weeks of age. Experiments were approved by the Ethics Committee of the Barcelona Biomedical Research Park and performed according to the Spanish and European legislations and in accordance with the guidelines provided by the Animal Care and Use Committee of the National Cancer Institute under protocol LG-009 and in accordance with the guidelines of the Mount Sinai Animal Care and Use Committee.

### Cell isolation and culture

Mouse splenic resting B cells were negatively isolated with magnetic beads^20,58^ (Cat. 130-090-862, Miltenyi Biotec), whereas human follicular and MZ B cells were FACSorted as further detailed below. Human splenic total IgD^+^ B cells were MACSorted. Briefly, splenocytes were first incubated with biotin-conjugated anti-IgD antibody (Supplementary Table 2) and then anti-biotin beads. Each labeling step included 15-min incubation at 4 °C. Magnetic column-based selections were performed and resulting positive fracion was isolated (Miltenyi Biotec). Splenocytes, 2E2 B cells and human embryonic kidney 293 cells were kept in culture as already reported^4,56^. Human MZ, naive, or IgD^+^ B cells were incubated with 500 ng/ml APRIL MegaLigand (Alexis) with or without 0.1 μg/ml CpG (Invitrogen). When indicated, 10 nM rapamycin (Sigma), 10 nM Torin 1 (Cell Signaling), or 25 μM Ly294002 (Calbiochem) were added 30 min before treatment with APRIL and/or CpG. Mouse B cells were stimulated with 100 ng/ml APRIL (R&D) in the presence or absence of 0.5 μg/ml LPS (Sigma-Aldrich).

### Gene expression profiling

cRNA from unstimulated or stimulated splenic follicular and MZ B cells was hybridized to the Agilent SurePrint G3 Human gene expression 8 × 60 K microarray and data were processed as reported^9^. Differentially expressed genes were identified based on >1.5-fold change and adjusted *p* value <0.05 (Supplementary Data 1 and Supplementary Table 1). Results were corrected for multiple testing according to the false discovery rate (FDR) method. The gene expression omnibus accession number for the transcriptional profiles reported in this paper is GSE85289. Gene categories were analyzed using ingenuity pathway analysis (Qiagen) and compared to available data sets through GSEA. Briefly, Agilent probes corresponding to an identical gene were summarized using their median intensity value so as to obtain one intensity per gene symbol. Those 36337 gene intensities were then fed to the GSEA software. The analysis was performed using standard parameters: the Signal2Noise parameter was used to calculate the gene’s differential expression with respect to the two phenotypes. Permutations were done at the gene set level. Genes sets analyzed were extracted from the MSigDB collections (http://software.broadinstitute.org/gsea/msigdb/collections.jsp). Transcriptional profiles of mTORC1 (HALLMARK_MTORC1_SIGNALING) and PC (GSE22886) signatures were compared through GSEA.

### Flow cytometry and cell sorting

Cell suspensions were treated with red blood cell lysis buffer (eBioscience), washed and incubated with Fc blocking reagent (BioLegend) and appropriate labeled mAbs (Supplementary Tables 2 and 3) for 20 min at 4 °C. Dead cells were excluded with 4′-6′-diamidine-2′-phenylindole (DAPI) (Sigma). To stain intracellular antigens, cells were first incubated with labeled mAbs to specific surface molecules, fixed and permeabilized with fix/perm buffer (BioLegend), and further incubated with specific labeled mAbs to intracellular antigens (Supplementary Tables 3 and 4). For organelle detection, cells were stained with LysoTracker Red DND-99, ER-Tracker Green, MitoTracker Green FM, and BODIPY FL C5-Ceramide complexed to bovine serum albumin (BSA) according to the manufacturer’s instructions (Thermo Fischer Scientific). Cells were acquired with an LSR Fortessa or FACSCalibur flow cytometers (BD Biosciences) and data analyzed with FlowJo software (TreeStar). Gates and quadrants were drawn to give ≤1% total positive cells in samples incubated with isotype-matched control mAbs. Human splenic CD19^+^IgD^hi^CD27^−^ naive follicular B cells and CD19^+^IgD^lo^CD27^+^ MZ B cells were first stained with appropriate fluorochrome-labeled mouse antibodies (Supplementary Tables 2 and 3) and later sorted with a FACSAria II (BD Biosciences) upon exclusion of dead cells through DAPI staining. The purity of sorted cells was consistently >95%. FACS gating strategies are presented in Supplementary Fig. 11.

### Viability and proliferation assays

Cell survival was measured with Annexin-V Apoptosis Detection Kit II and 7-amino-actinomycin D (Cat. 556547, BD Pharmingen). Cell proliferation was assessed by staining cells with 1 μM CFSE using a CellTrace CFSE Cell Proliferation Kit (Cat. C34554, Invitrogen).

### Immunofluorescence

Human and mouse spleens were collected and frozen in Tissue-Tek OCT Compound (Sakura). Five-micrometer sections were cut in a cryostat microtome (Leica). Slides were fixed in acetone and stored at −80 °C. Before staining, samples were extensively washed with PBS and blocked with 5% FBS in PBS for 30 min at room temperature. Endogenous biotin was blocked by using an Avidin/Biotin blocking reagent (Vector Laboratories). Sorted MZ B cells were resuspended in cell adhesive solution (Crystalgen), applied onto slides (Gold Seal Products), fixed with 1% paraformaldehyde, and permeabilized with 0.2% Triton-100 in PBS. Slides were incubated with various antibody combinations that were later detected using appropriate reagents (Supplementary Tables 2 and 3). Nuclear DNA was stained with DAPI. Coverslips were applied with FluorSave reagent (Calbiochem) and images were obtained with a Leica TCS SP5 microscope (Leica) and further analyzed with ImageJ software.

### ELISA

To measure antigen-specific antibodies, plates were coated overnight with 5 μg/ml PCh_16_-BSA or 4 μg/ml TNP_18_-BSA (Biosearch Technologies) diluted in PBS at 4 °C, washed three times with PBS and 0.1% Tween-20 (Thermo Fisher Scientific), blocked for 2 h at room temperature with PBS and 1% BSA, and washed four times. Plates were then incubated at room temperature for 2 h with appropriately diluted sera and washed four times prior to incubation for 1 h at 37 °C with appropriate detection antibodies (Supplementary Table 3). After four additional washes, plates were incubated with streptavidin-conjugated peroxidase (Vector) and washed four more times. Reactions were developed with horseradish peroxidase substrate 3,3′,5,5′-tetramethylbenzidine (Beckton-Dickinson) and stopped using 2 N H_2_SO_4_. Plates were read at 450 nm.

### Immunoprecipitations and kinase assays

Cells were washed once in ice-cold PBS and lysed in RIPA buffer (50 mM Tris pH 7.4, 150 mM NaCl, 1% NP-40, 0.5% sodium deoxycholate, 0.1% SDS) supplemented with protease and phosphatase inhibitors (Thermo Fisher Scientific). Lysates were first pre-cleared for 30 min at 4 °C with 100 μl protein A/G-Sepharose beads (GE Healthcare), then incubated overnight with 5 μg control IgG mAb or specific antibodies (Supplementary Tables 2 and 3). The next day, lysates were incubated for 1 h at 4 °C with 100 μl protein A/G-Sepharose beads to precipitate immune complexes. The resulting proteins were separated by sodium dodecyl sulfate-polyacrylamide gel electrophoresis (SDS-PAGE) and analyzed by IB. Kinase assays were performed after precipitating whole protein lysates from IgD^+^ 2E2 cells (2 × 10^7^) with control antibodies or antibodies to activated IRAK-1 or IRAK-4 (Supplementary Tables 2 and 3). The phosphorylated substrate was then analyzed by IB analysis using a mAb to phosphorylated myelin basic protein (MBP, Millipore).

### Immunoblotting and phospho-kinase array

Immunoblots were performed using protein lysates from splenic IgD^+^ cells (2 × 10^7^), IgD^+^ 2E2 B cells (2 × 10^7^), or transfected 293 cells (5 × 10^6^) as reported previously. Equal amounts of proteins were fractionated through SDS-PAGE and transferred onto polyvinylidene difluoride membranes (BioRad). After blocking, membranes were incubated with a primary antibody, followed by a corresponding secondary antibody (Supplementary Tables 2 and 3). Membranes were developed using an enhanced chemiluminescence detection system (Amersham). AKT, p70S6K, p38, CREB, and STAT5 phosphorylation was further analyzed using a Proteome Profiler Human Phospho-Kinase Array Kit (Cat. ARY003B, R&D Systems). A transmission-mode scanner (Epson) and Fiji analysis software (ImageJ 1.51d) was used to perform a densitometric analysis of immunoreactions. Full-length uncropped blots are presented in Supplementary Fig. 12.

### RNA extraction and PCR

Total cellular RNA was isolated with the RNeasy Micro kit (Qiagen) by following the manufacturer’s protocol. Approximately 2 ng of RNA were reversed transcribed into cDNA using TaqMan^®^ Reverse Transcription Reagents and Random hexamers (Thermo Fisher). qRT-PCRs were performed with specific primer pairs (Supplementary Tables 4 and 5). For the analysis of germline transcripts, PCRs were carried out using specific primers (Supplementary Table 4) in a 25 µl PCR volume with AmpliTaq Gold PCR Mastermix (Thermo Fisher). Nested PCR analysis of Iγ1/2-Cµ circle transcript was carried out using two sets of specific primer pairs (Supplementary Table 4) and the following cycling conditions. In the first PCR round, external primers were used in an initial denaturing step at 95 °C for 9 min followed by 30 cycles comprised of 94 °C for 30 s, 60 °C for 1 min, and 72 °C for 10 min. In the second RT-PCR round, internal primers were used in an initial denaturing step at 95 °C for 9 min followed by 25 cycles comprised of 94 °C for 30 s, 60 °C for 1 min, and 72 °C for 10 min. For the quantification of mouse gene products, total RNA was extracted with TRIzol reagent (Invitrogen) and cDNAs generated with TaqMan Reverse Transcription Reagents (Applied Biosystems). qRT-PCR was run in a LightCycler 480 real-time PCR system (Roche Diagnostics) with SYBR Green I Master Kit (Roche Diagnostics) and specific primer pairs (Supplementary Table 5). Gene expression was normalized to that of the gene encoding β-actin or glyceraldehyde 3-phosphate dehydrogenase (GAPDH).

### EMSA

Nuclear protein extracts from splenic IgD^+^ B cells (2 × 10^7^) were used to perform NF-κB-specific EMSAs and supershift assays. Oligonucleotides encompassing commercially available consensus NF-κB binding (Santa Cruz) were labeled with [α-32P] ATP and used at ~50,000 c.p.m. in each reaction. Reaction samples were prepared and electrophoresed through a 5% non-denaturing polyacrylamide gel. The composition of DNA-bound protein complexes was determined by incubation of the reaction mixture with 1 µg polyclonal antibody to p50 or p52 subunits of NF-κB before the addition of radiolabeled probe. Increasing concentrations of unlabeled (cold) probe were used to test the specificity of the reaction.

### Cell transfections and luciferase reporter assays

Two hundred ninety-three cells were transiently transfected using SuperFect transfection reagent (Qiagen) as reported previously^4^. The 2E2 B-cell line was co-transfected with 1 µg TACI-expressing vector and 500 ng NF-κB-driven reporter plasmid expressing firefly luciferase (Promega) together with 200 ng pRL-TK reporter plasmid expressing renilla luciferase (Promega). Nucleofection was performed using the Pulse controller Plus (BioRad). Transfected cells kept for 18 h at 37 °C and 5% CO_2_ were treated with 10 nM rapamycin. A dual luciferase assay system (Promega) was used to measure luciferase activity and results were normalized to the renilla signal (Supplementary Table 6).

### FRET assays

Comparable amounts of plasmids expressing human TLR9 C-terminally tagged with eYFP and human TACI C-terminally tagged with mCherry were co-transfected in 293 cells as published earlier^57^. Briefly, the pCI-TACI-mCherry plasmid was generated using an In-Fusion^®^ Cloning Kit (Clontech). Briefly, pCI-Neo was linearized using *Xho*I and *Not*I restriction enzymes (NEB). The TACI cassette (CD5L-HA-TACI) was PCR amplified from a pRetig-TACI plasmid using a reverse primer targeting the 5′ end of mCherry and containing a *Hind*III restriction site (Supplementary Table 7). mCherry DNA was PCR amplified from a pLVX-IRES-mCherry plasmid (from Enric Esplugues, Icahn School of Medicine) using a forward primer targeting the 3′ end of TACI and containing a *Hind*III restriction site (Supplementary Table 7). A third PCR was performed to obtain a TACI-mCherry cassette encompassing 5′ end 3′ ends overlapping with segments of the pCI plasmid (Supplementary Table 7). Negative controls were performed using 293 cells co-transfected with empty pZ-eYFP and pLVX-IRES-mCherry plasmids. After 48 h, co-transfected 293 cells were gently washed in staining solution (1 × PBS, 0.5% BSA, and 2 mM ethylenediaminetetraacetic acid) and 1 × PBS, suspended to 5 × 10^5^ cells/ml, and filtered through 35-μm filter top tubes. To measure eYFP, fluorescence from cells excited with a 488-nm laser was collected in the eYFP channel with a standard 530/20 filter. The FRET signal was measured with a 610/620 filter. FRET measurements were performed using an LSRII analyzer (BD Biosciences). To measure mCherry, fluorescence from cells excited with a 532-nm laser was collected with a 610/620 filter. A minimum of 5 × 10^4^ cells was evaluated within the adjusted gate and data were analyzed using FlowJo software (TreeStar).

### Transmission electron microscopy

Cell-sorted MZ and naive follicular B cells were fixed with 2.5% glutaraldehyde for 2 h at 4 °C, post fixed in 1% osmium tetroxide for 2 h at 4 °C, dehydrated with ethanol, and embedded in epoxy resin. Samples were examined with a JEM-1011 transmission electron microscope (JEOL). At least 30 cells were captured for ultrastructural analysis.

### Mouse immunization and rapamycin treatment

Mice were intraperitoneally (i.p.) immunized with 50 µg TNP_36_-Ficoll T-cell-independent antigen (Biosearch Technologies) or with 100 µg TNP_18_-KLH T-cell-dependent antigen (Biosearch Technologies) with adjuvant (Sigma) at day 0. Serum samples were collected from the tail vein (day 0) or by cardiac puncture (day 7 and day 10, respectively). Rapamycin (InvivoGen) was diluted in 100-µl phosphate buffer solution (PBS) with vehicle including 0.25% PEG and 0.25% Tween 80 (Sigma) and injected i.p. at a dose of 1 mg/kg. Injections were given daily, starting 1 day before immunization and ending 7 or 10 days after immunization, respectively. Control mice were injected with the same volume of PBS and vehicle.

### Statistical analysis

Differences between means from independent groups were assessed using Prism 5.03 software (GraphPad) and unpaired two-tailed Student’s *t* tests, unless otherwise indicated. *P* values of <0.05 were considered significant. In animal experiments, at least three mice per group were randomly distributed into treatment groups so that all groups were age-matched and sex-matched. No specific randomization or blinding protocol was used and no animals were excluded from analysis.

### Data availability

Microarray data that support the findings of this study have been deposited in NCBI GEO with the primary accession code GSE85289. The authors declare that all other data supporting the findings of this study are available within the article and its Supplementary Information files or from the corresponding author on request.

## Electronic supplementary material

Supplementary information

Peer review file

Description of Additional Supplementary Files

Supplementary Data 1

## References

[1] Cerutti A, Cols M, Puga I (2013). Marginal zone B cells: virtues of innate-like antibody-producing lymphocytes. Nat. Rev. Immunol..

[2] Mackay F, Schneider P (2009). Cracking the BAFF code. Nat. Rev. Immunol..

[3] Balázs M, Martin F, Zhou T, Kearney JF (2002). Blood dendritic cells interact with splenic marginal zone B cells to initiate T-independent immune responses. Immunity.

[4] He B (2010). The transmembrane activator TACI triggers immunoglobulin class switching by activating B cells through the adaptor MyD88. Nat. Immunol..

[5] Figgett WA (2013). The TACI receptor regulates T-cell-independent marginal zone B cell responses through innate activation-induced cell death. Immunity.

[6] von Bulow GU, van Deursen JM, Bram RJ (2001). Regulation of the T-independent humoral response by TACI. Immunity.

[7] Rawlings DJ, Schwartz MA, Jackson SW, Meyer-Bahlburg A (2012). Integration of B cell responses through Toll-like receptors and antigen receptors. Nat. Rev. Immunol..

[8] Martin F, Oliver AM, Kearney JF (2001). Marginal zone and B1 B cells unite in the early response against T-independent blood-borne particulate antigens. Immunity.

[9] Chorny A (2016). The soluble pattern recognition receptor PTX3 links humoral innate and adaptive immune responses by helping marginal zone B cells. J. Exp. Med..

[10] Di Niro R (2015). Salmonella infection drives promiscuous B cell activation followed by extrafollicular affinity maturation. Immunity.

[11] Martins G, Calame K (2008). Regulation and functions of Blimp-1 in T and B lymphocytes. Annu. Rev. Immunol..

[12] Tellier J (2016). Blimp-1 controls plasma cell function through the regulation of immunoglobulin secretion and the unfolded protein response. Nat. Immunol..

[13] Shapiro-Shelef M (2003). Blimp-1 is required for the formation of immunoglobulin secreting plasma cells and pre-plasma memory B cells. Immunity.

[14] Weichhart T, Hengstschlager M, Linke M (2015). Regulation of innate immune cell function by mTOR. Nat. Rev. Immunol..

[15] Thomson AW, Turnquist HR, Raimondi G (2009). Immunoregulatory functions of mTOR inhibition. Nat. Rev.Immunol..

[16] Laplante M, Sabatini DM (2012). mTOR signaling in growth control and disease. Cell.

[17] Alessi DR, Kulathu Y (2013). Structural biology: security measures of a master regulator. Nature.

[18] Zeng H, Chi H (2014). mTOR signaling and transcriptional regulation in T lymphocytes. Transcription.

[19] Keating R (2013). The kinase mTOR modulates the antibody response to provide cross-protective immunity to lethal infection with influenza virus. Nat. Immunol..

[20] Zhang S (2013). B cell-specific deficiencies in mTOR limit humoral immune responses. J. Immunol..

[21] Jones DD (2016). mTOR has distinct functions in generating versus sustaining humoral immunity. J. Clin. Invest..

[22] Limon JJ, Fruman DA (2012). Akt and mTOR in B cell activation and differentiation. Front. Immunol..

[23] Benhamron S, Tirosh B (2011). Direct activation of mTOR in B lymphocytes confers impairment in B-cell maturation andloss of marginal zone B cells. Eur. J. Immunol..

[24] Iwata TN, Ramirez-Komo JA, Park H, Iritani BM (2017). Control of B lymphocyte development and functions by the mTOR signaling pathways. Cytokine Growth Factor Rev..

[25] Figgett WA (2015). Deleting the BAFF receptor TACI protects against systemic lupus erythematosus without extensive reduction of B cell numbers. J. Autoimmun..

[26] Groom JR (2007). BAFF and MyD88 signals promote a lupuslike disease independent of T cells. J. Exp. Med..

[27] Martin F, Kearney JF (2002). Marginal-zone B cells. Nat. Rev. Immunol..

[28] Descatoire M (2014). Identification of a human splenic marginal zone B cell precursor with NOTCH2-dependent differentiation properties. J. Exp. Med..

[29] Ochiai K (2013). Transcriptional regulation of germinal center B and plasma cell fates by dynamical control of IRF4. Immunity.

[30] Jash A (2016). ZBTB32 restricts the duration of memory B cell recall responses. J. Immunol..

[31] Otipoby KL (2015). The B-cell antigen receptor integrates adaptive and innate immune signals. Proc. Natl Acad. Sci. USA.

[32] Kometani K (2013). Repression of the transcription factor Bach2 contributes to predisposition of IgG1 memory B cells toward plasma cell differentiation. Immunity.

[33] Zhang X, Zhang J, Zhang L, van Dam H, ten Dijke P (2013). UBE2O negatively regulates TRAF6-mediated NF-kappaB activation by inhibiting TRAF6 polyubiquitination. Cell Res..

[34] Lamming DW, Sabatini DM (2013). A central role for mTOR in lipid homeostasis. Cell Metab..

[35] Cantor J (2009). CD98hc facilitates B cell proliferation and adaptive humoral immunity. Nat. Immunol..

[36] Cao W (2008). Toll-like receptor-mediated induction of type I interferon in plasmacytoid dendritic cells requires the rapamycin-sensitive PI(3)K-mTOR-p70S6K pathway. Nat. Immunol..

[37] Weichhart T (2008). The TSC-mTOR signaling pathway regulates the innate inflammatory response. Immunity.

[38] Vallabhapurapu S, Karin M (2009). Regulation and function of NF-kappaB transcription factors in the immune system. Annu. Rev. Immunol..

[39] Chaudhuri J, Alt FW (2004). Class-switch recombination: interplay of transcription DNA deamination and DNA repair. Nat. Rev. Immunol..

[40] Ewald SE (2008). The ectodomain of Toll-like receptor 9 is cleaved to generate a functional receptor. Nature.

[41] Schutze S, Tchikov V, Schneider-Brachert W (2008). Regulation of TNFR1 and CD95 signalling by receptor compartmentalization. Nat. Rev. Mol. Cell Biol..

[42] Stein JV (2002). APRIL modulates B and T cell immunity. J. Clin. Invest..

[43] Sinclair LV (2013). Control of amino-acid transport by antigen receptors coordinates the metabolic reprogramming essential for T cell differentiation. Nat. Immunol..

[44] Cho SH (2016). Germinal centre hypoxia and regulation of antibody qualities by a hypoxia response system. Nature.

[45] Limon JJ (2014). mTOR kinase inhibitors promote antibody class switching via mTORC2 inhibition. Proc. Natl Acad. Sci. USA.

[46] Meyer-Bahlburg A, Bandaranayake AD, Andrews SF, Rawlings DJ (2009). Reduced c-myc expression levels limit follicular mature B cell cycling in response to TLR signals. J. Immunol..

[47] Woodland RT (2008). Multiple signaling pathways promote B lymphocyte stimulator dependent B-cell growth and survival. Blood.

[48] Otipoby KL (2008). BAFF activates Akt and Erk through BAFF-R in an IKK1-dependent manner in primary mouse B cells. Proc. Natl Acad. Sci. USA.

[49] Zeng, Q. et al. Rapamycin attenuates BAFF-extended proliferation and survival via disruption of mTORC1/2 signaling in normal and neoplastic B-lymphoid cells. *J. Cell Physiol*. https://doi.org/10.1002/jcp.25913 (2017). 10.1002/jcp.25913 PMC5600640 28300280

[50] Schmitz F (2008). Mammalian target of rapamycin (mTOR) orchestrates the defense program of innate immune cells. Eur. J. Immunol..

[51] Chang J (2013). MyD88 is essential to sustain mTOR activation necessary to promote T helper 17 cell proliferation by linking IL-1 and IL-23 signaling. Proc. Natl Acad. Sci. USA.

[52] Maglione PJ (2014). IRAK-4 and MyD88 deficiencies impair IgM responses against T-independent bacterial antigens. Blood.

[53] Weller S (2012). IgM+IgD+CD27+B cells are markedly reduced in IRAK-4-, MyD88- and TIRAP- but not UNC-93B-deficient patients. Blood.

[54] Romberg N (2013). CVID-associated TACI mutations affect autoreactive B cell selection and activation. J. Clin. Invest..

[55] Ersching J (2017). Germinal center selection and affinity maturation require dynamic regulation of mTORC1 kinase. Immunity.

[56] Magri G (2014). Innate lymphoid cells integrate stromal and immunological signals to enhance antibody production by splenic marginal zone B cells. Nat Immunol.

[57] Garcia-Carmona Y (2015). Differential induction of plasma cells by isoforms of human TACI. Blood.

[58] Zhang S (2011). Constitutive reductions in mTOR alter cell size, immune cell development, and antibody production. Blood.

