# Supplementary material for: mTOR intersects antibody-inducing signals from TACI in marginal zone B cells
Source: Nat Commun. 2017 Nov 13;8:1462. doi: 10.1038/s41467-017-01602-4 (PMC5684130; doi:10.1038/s41467-017-01602-4)
Supplement: Supplementary file 1 — Supplementary information [file 41467_2017_1602_MOESM1_ESM.pdf]

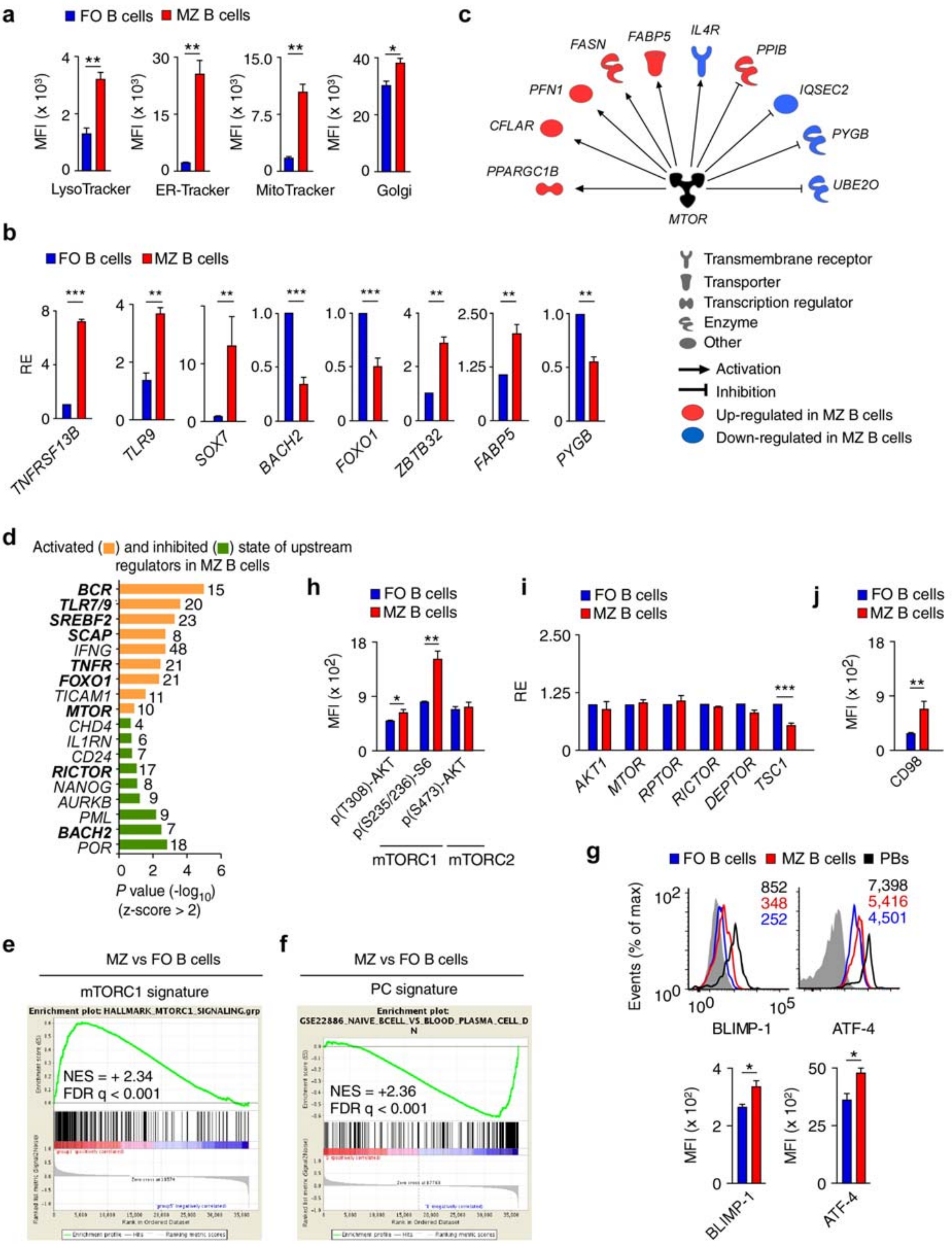
a
FO B cells
MZ B cells
MFI (x 10³)
LysoTracker
ER-Tracker
MitoTracker
Golgi
b
RE
TNFRSF13B
TLR9
SOX7
BACH2
FOXO1
ZBTB32
FABP5
PYGB
c
FASN
FABP5
IL4R
PPIB
PFN1
IQSEC2
CFLAR
PYGB
PPARGC1B
UBE2O
MTOR
Transmembrane receptor
Transporter
Transcription regulator
Enzyme
Other
Activation
Inhibition
Up-regulated in MZ B cells
Down-regulated in MZ B cells
d
Activated ( ) and inhibited ( ) state of upstream regulators in MZ B cells
BCR
TLR7/9
SREBF2
SCAP
IFNG
TNFR
FOXO1
TICAM1
MTOR
CHD4
IL1RN
CD24
RICTOR
NANOG
AURKB
PML
BACH2
POR
P value (-log10)
(z-score > 2)
h
MFI (x 10²)
p(T308)-AKT
p(S235/236)-S6
p(S473)-AKT
mTORC1
mTORC2
i
AKT1
MTOR
RPTOR
RICTOR
DEPTOR
TSC1
j
CD98
g
FO B cells
MZ B cells
PBs
Events (% of max)
852
348
252
7,398
5,416
4,501
BLIMP-1
ATF-4
e
MZ vs FO B cells
mTORC1 signature
Enrichment plot: HALLMARK_MTORC1_SIGNALING.grp
NES = + 2.34
FDR q < 0.001
f
MZ vs FO B cells
PC signature
Enrichment plot: GSE22886_NAIVE_BCELL_VS_BLOOD_PLASMA_CELL_DN
NES = +2.36
FDR q < 0.001

**Supplementary Figure 1. MZ B cells couple elevated TACI and TLR9 expression with increased activation of mTOR-related metabolic and immune signaling pathways.** (**a**) MFI of LysoTracker, ER-Tracker, MitoTracker and Golgi in human splenic FO and MZ B cells analyzed by FCM. (**b**) qRT-PCR of mRNAs for TACI (*TNFRSF13B*), TLR9, SOX7, BACH2, FOXO1, ZBTB32, FABP5 and PYGB in human splenic FO and MZ B cells. Results are normalized to mRNA for β-actin and presented as relative expression (RE) compared to a reference set of FO B cells. (**c**) Ingenuity Pathway Analysis of the transcriptome from human splenic MZ B cells showing a gene network pertaining to metabolic and immune pathways downstream of mTOR. Red and blue indicate increased and decreased expression in MZ B cells compared to FO B cells. Arrows illustrate activating effects, whereas blunt ended lines show inhibitory effects. (**d**) Ingenuity's Upstream Regulator Analysis showing activated (yellow) and inhibited (green) state of upstream regulators of gene products linked to metabolic and immune pathways differentially expressed in MZ and FO B cells. A Fisher's Exact Test p-value was calculated to assess the significance of enrichment in gene expression. Bold gene products are discussed in main text. (**e**,**f**) GSEA of genes linked to mTORC1 activation (HALLMARK_MTORC1) (**e**) or PC differentiation (GSE22886) (**f**) in MZ and FO B cells. Normalized enrichment score (NES) indicates correlation between individual gene sets. Positive correlation, NES > 0 (red bars); negative correlation, NES < 0 (blue bars). (**g**) FCM histograms and MFI of BLIMP-1 and ATF-4 in human splenic FO, MZ B cells and PBs. (**h**) MFI of p(T308)-AKT, p(S235/S236)-S6 and p(S473)-AKT in human splenic FO and MZ B cells analyzed by FCM. (**i**) qRT-PCR of mRNAs for AKT (*AKT1*), mTOR, RAPTOR (*RPTOR*), RICTOR, DEPTOR and TSC1 in human splenic FO and MZ B cells. Results are normalized to mRNA for β-actin and presented as RE compared to a reference set of FO B cells. (**j**) MFI of CD98 receptor on human splenic FO and MZ B cells. Cells were gated as in **Supplementary Fig. 11a** (**a**,**g**,**h**,**j**). Data summarize at least two experiments involving at least two donors in each experimental group (**a**,**b**,**e**,**f**,**h**-**j**) or are from at least three biological replicates for each cell type (**c**,**d**) or show one representative experiment from at least three yielding similar results (**g**). Error bars, s.e.m.; $*$ $p < 0.05$, $**$ $p < 0.01$, $***$ $p < 0.001$ (two-tailed Student's *t* test).

**a**

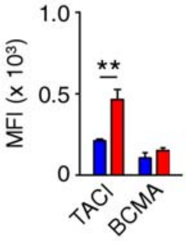

MFI (x 10³)
1.0
0.5
0
**
TACI
BCMA


**b**

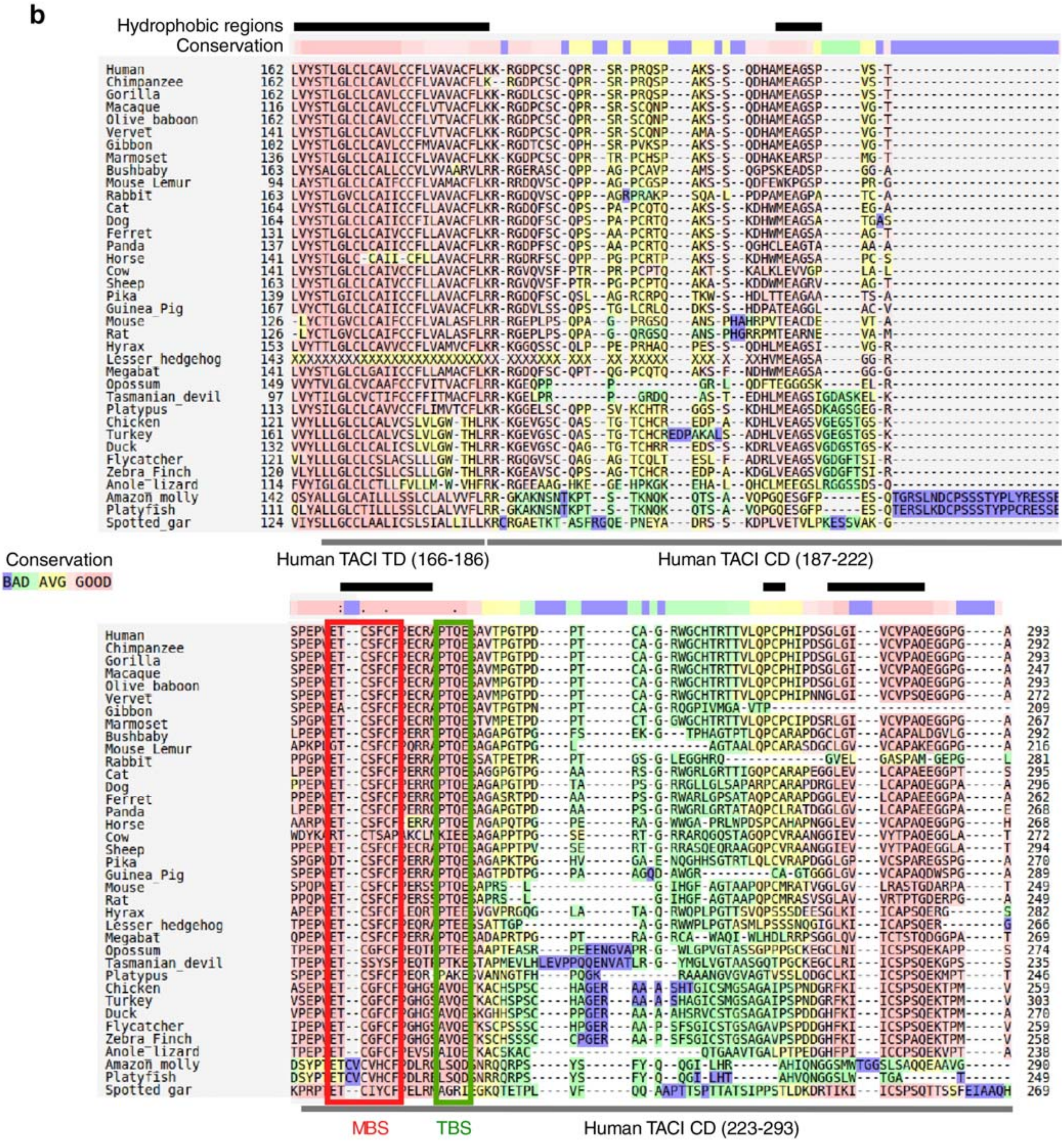

Hydrophobic regions
Conservation
Conservation
BAD AVG GOOD
Human TACI TD (166-186)
Human TACI CD (187-222)
MBS
TBS
Human TACI CD (223-293)

**Supplementary Figure 2. The MBS of TACI is highly conserved among vertebrates.** (**a**) MFI of TACI and BCMA expression on human splenic FO and MZ B cells analyzed by FCM. Cells were gated as in **Supplementary Fig. 11a**. (**b**) T-Coffee multiple sequence alignment of the cytoplasmic domain from annotated *TNFRSF13B* orthologs listed in Ensembl database. The hydrophobocity of the human TACI protein is depicted in black and the degree of amino acidic conservations is shown as a color range. Red and green boxes respectively mark MBS (MyD88-binding site) and TBS (TRAF2-binding site) as defined by published studies. Data are from at least four biological replicates for each cell type (**a**). Error bars, s.e.m.; ∗∗ $p < 0.01$ (two-tailed Student's *t* test).

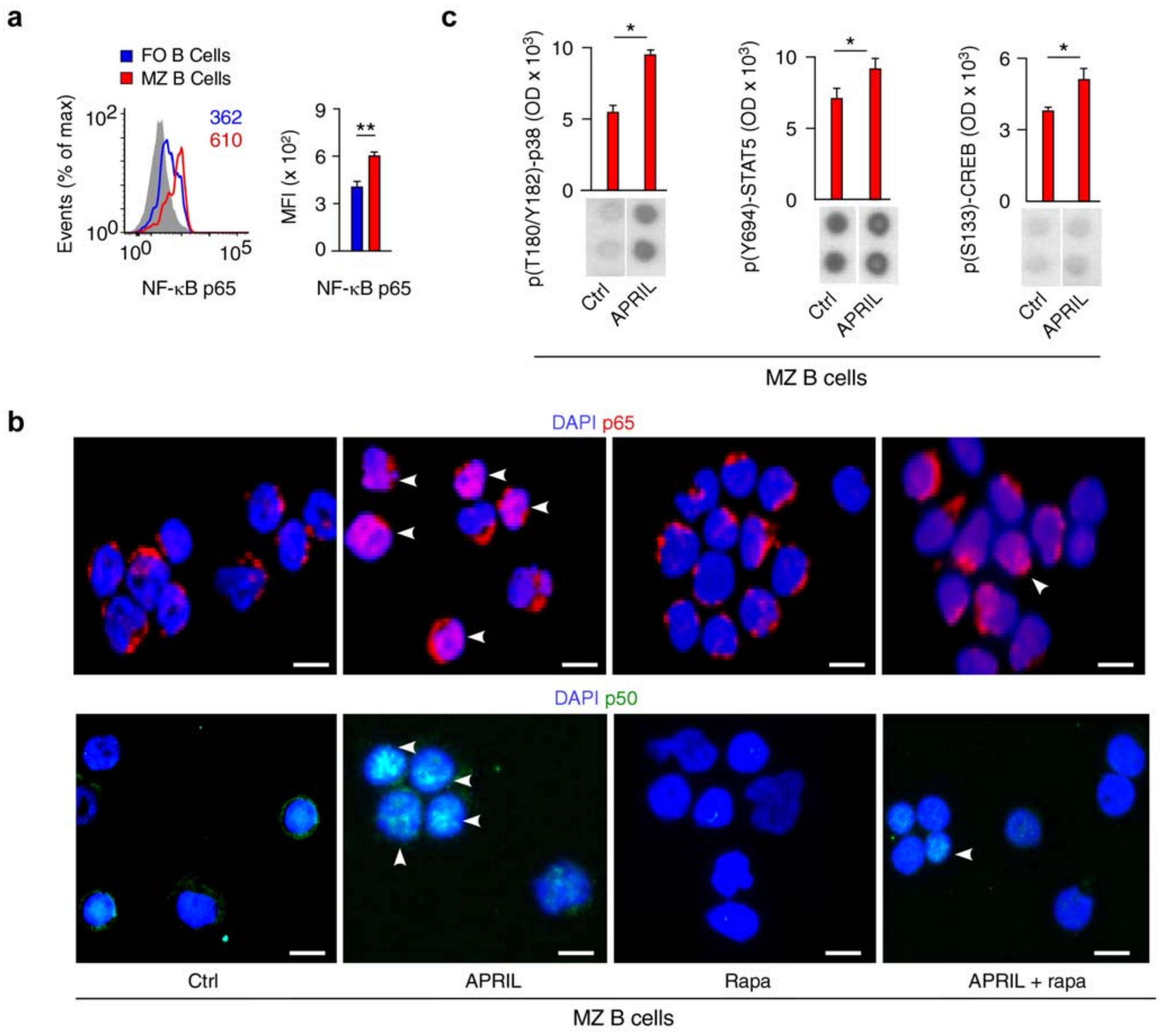


**Supplementary Figure 3. TACI ligation enhances NF-κB activation in MZ B cells through mTORC1.** (**a**) FCM and MFI of p65 NF-κB in human splenic FO and MZ B cells. Cells were gated as in **Supplementary Fig. 11a**. (**b**) IFA of p65 NF-κB (red, upper panels), p50 NF-κB (green, bottom panels) and DAPI (blue) in human splenic MZ B cells cultured with or without APRIL and/or rapamycin (rapa) APRIL for 60 min. Ctrl, medium alone. Images are from one of two experiments yielding similar results. Scale bars, 5 μm. (**c**) Phospho-proteome analysis of p(T180/Y182)-p38 MAPK, p(Y694)-STAT5 and p(S133)-CREB in human splenic MZ B cells stimulated with or without APRIL for 15 min. Ctrl, medium

alone. Bars indicate the mean pixel density of dot blots underneath each graph. OD, optical density. Data are from at least four biological replicates for each cell type (**a**) or experimental condition (**c**) or show one representative experiment of at least three with similar results (**b**). Error bars, s.e.m.; $* p < 0.05$, $** p < 0.01$ (two-tailed Student's t test).

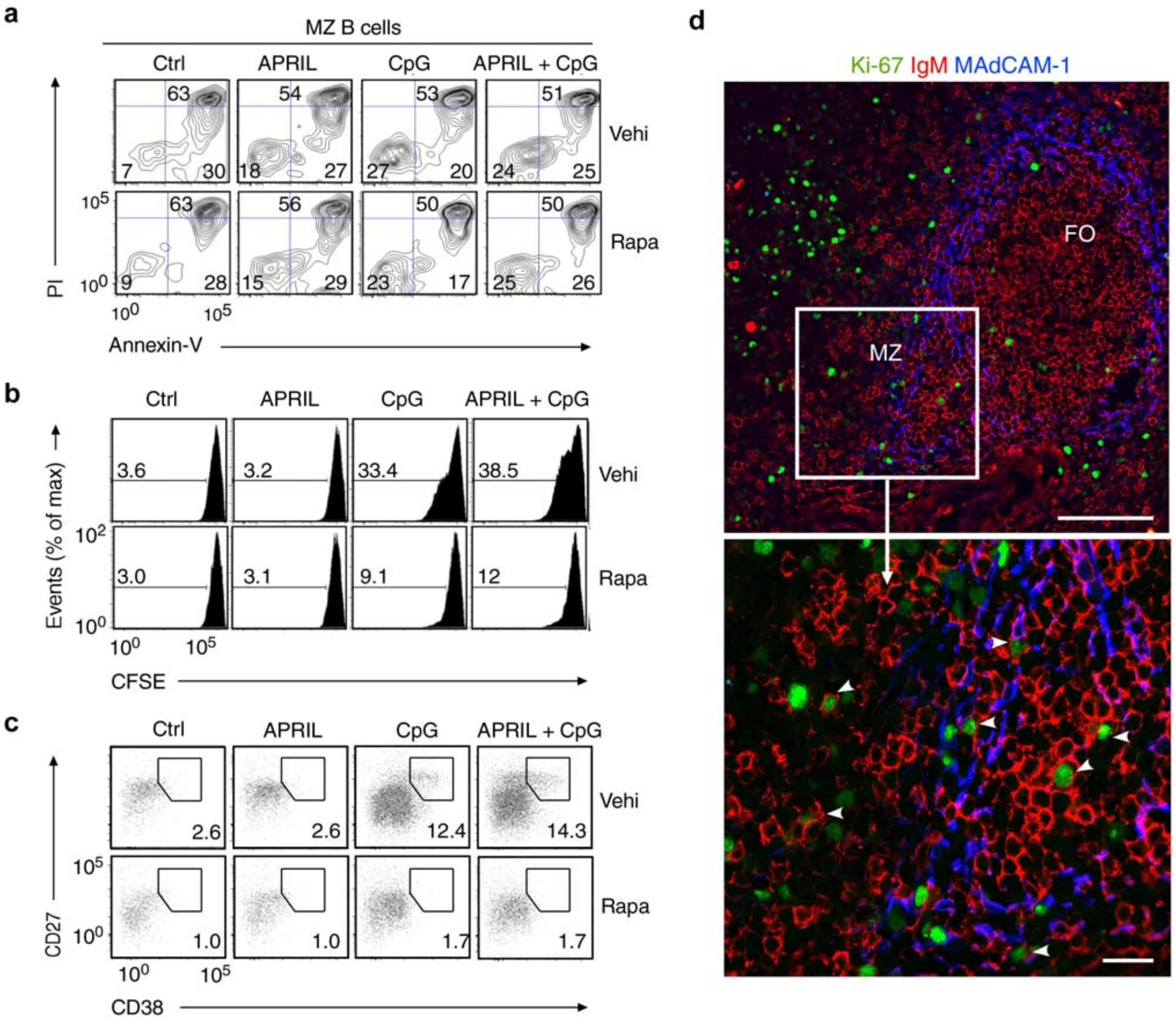


**Supplementary Figure 4. TACI and TLR9 cooperatively activate MZ B cell proliferation and PB differentiation through mTORC1.** (**a**) Flow cytometry of annexin-V and propidium iodide (PI) in human splenic MZ B cells cultured for 5 d with or without APRIL and/or CpG DNA in the presence of control vehicle (vehi) or rapamycin (rapa). Bottom-left, bottom-right and top-right numbers indicate frequency of viable annexin-$V^-PI^-$, early apoptotic annexin-$V^+ PI^-$ and late apoptotic annexin-$V^+PI^+$ cells, respectively. (**b**) FCM of human splenic MZ B cells stained with CFSE and cultured as in (**a**). Numbers indicate frequency of $CFSE^{lo}$ B cells that have diluted CFSE due to proliferation. (**c**) Flow cytometry of viable $DAPI^-CD19^+CD27^{hi}CD38^{hi}$ PBs generated by human splenic MZ B cells cultured for 5 d as in (**a**).

Numbers indicate PB frequency. (**d**) IFA of human splenic tissue stained for Ki-67 (green), IgM (red) and MAdCAM-1 (blue). Scale bars, 100 µm (upper panel) or 20 µm (lower panel). Arrowheads point to PBs. Data show one representative experiment of at least three with similar results (**a**-**d**).

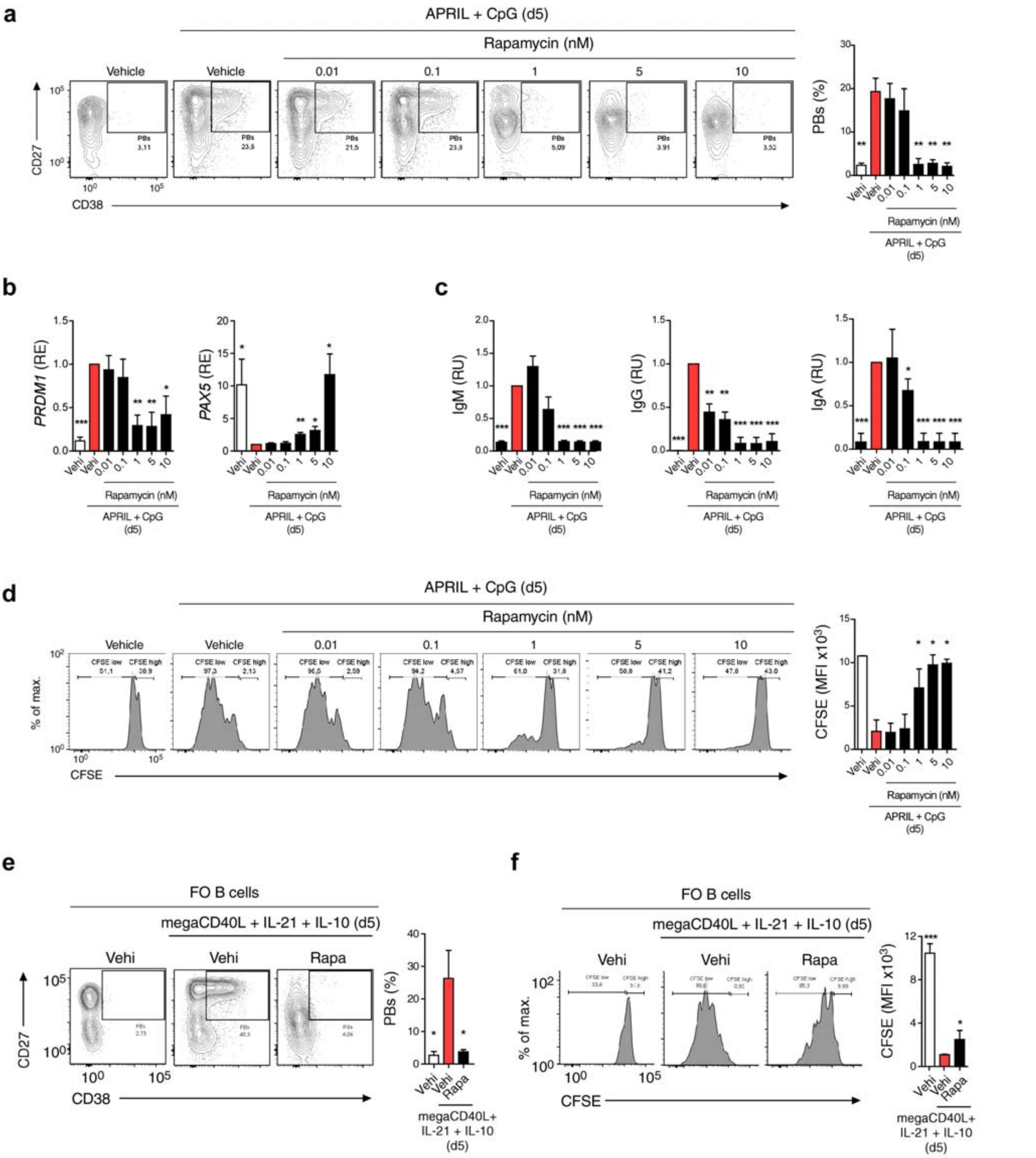


**Supplementary Figure 5. Blockade of mTORC1 by rapamycin can inhibit TACI-induced CSR without impairing MZ B cell proliferation.** (**a**) Flow cytometry of viable CD38$^+$CD27$^+$ plasmablasts (PBs) generated by human splenic MZ B cells following 5-day stimulation with APRIL plus CpG with or

without different concentrations or rapamycin. Bar plot summarizes frequency of PBs (%). (**b**) qRT-PCR of mRNAs for BLIMP-1 (*PRDM1*) and PAX-5 in human splenic MZ B cells stimulated as in (**a**). Results are normalized to mRNA for β-actin and presented as RE compared to stimulated cells without rapamycin (red bar). (**c**) ELISA of total IgM, IgG and IgA from supernatants obtained from human splenic MZ B cell cell cultures performed as in (**a**). Results are normalized as relative units (RU) by comparing to stimulated cells without rapamycin (red bar). (**d**) Proliferation of CFSE-labeled human splenic MZ B cells (labeling was performed with a low concentration of the cytosolic dye CFSE) stimulated as in (**a**). (**e**) Flow cytometry of viable $CD38^{+}CD27^{+}$ PBs generated by splenic FO B cells following stimulation with megaCD40L, IL-21 and IL-10 for 5 days. Bar plot shows frequency of PBs (%) (**f**) Proliferation of human splenic FO B cells stimulated as in (**e**). Bar plot shows MFI of CFSE. Data summarize at least two experiments with at least two replicates in each experimental group (**a-f**). Error bars, s.e.m.; ∗ $p < 0.05$, ∗∗ $p < 0.01$, ∗∗∗ $p < 0.001$ (two-tailed Student's t test).

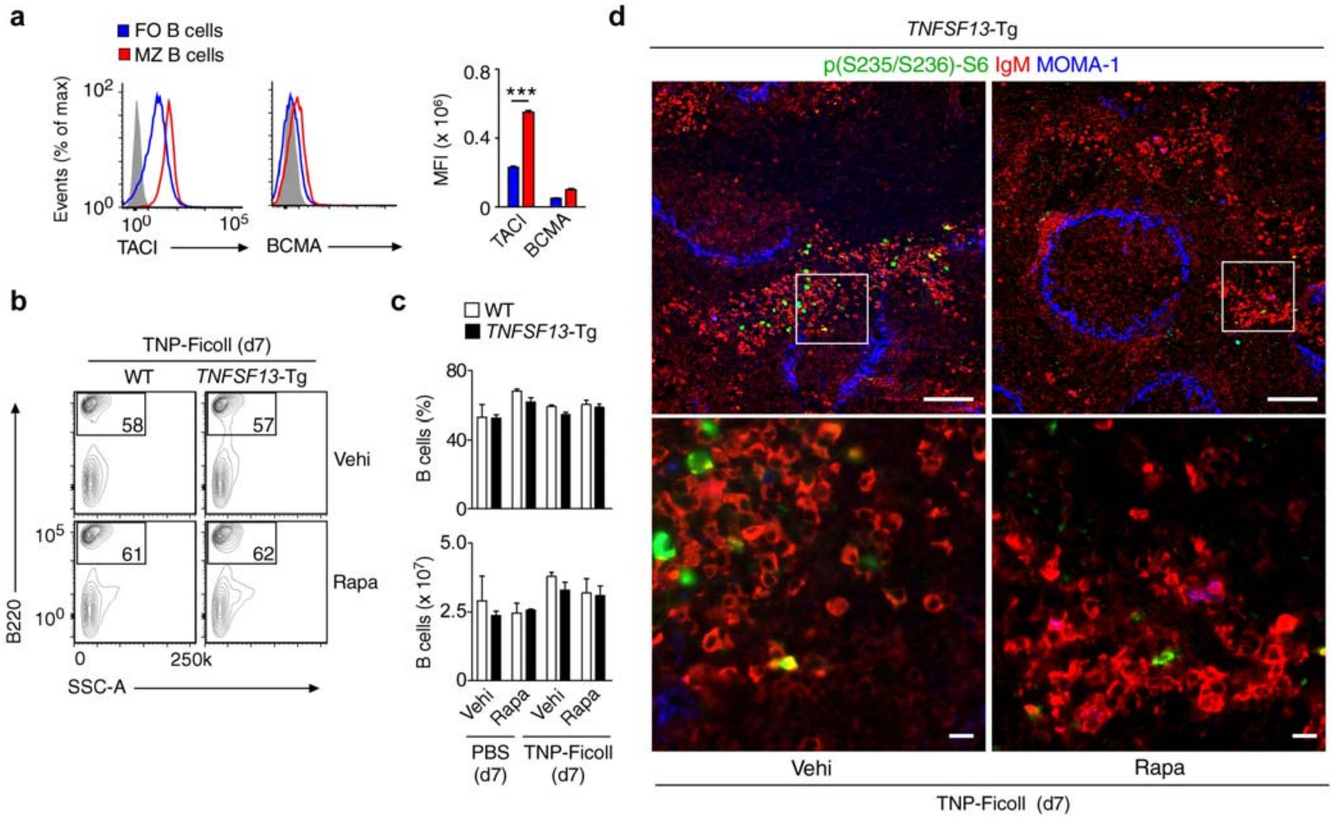


**Supplementary Figure 6. Blockade of mTORC1 by rapamycin does not affect TACI-induced splenic B cell survival.** (**a**) FCM and mean fluorescence intensity (MFI) of TACI and BCMA on splenic FO and MZ B cells from WT C57BL/6 mice. Cells were gated as in **Supplementary Fig. 11b.** (**b**) FCM of splenic B220$^+$ B cells from WT and *TNFSF13*-Tg mice following i.p. immunization with TNP-Ficoll for 7 days and daily i.p. injection of control vehicle (vehi) or rapamycin (rapa). SSC-A, side scatter-area. (**c**) FCM of frequency (top) and absolute number (bottom) of splenic B220$^+$ B cells from WT and *TNFSF13*-Tg mice treated as in (**b**). (**d**) IFA of IgM (red), p(S235/S236)-S6 (green) and MOMA-1 (blue) from splenic tissue of *TNFSF13*-Tg mice immunized with TNP-Ficoll and treated with vehi or rapa as in (**b**). Scale bars, 100 µm (upper panel) or 10 µm (lower panel). Data summarize at least two experiments with at least two animals in each experimental group (**a-d**) and show one representative replicate (**a,b,d**). Error bars, s.e.m.; ∗∗∗ $p < 0.001$ (two-tailed Student's *t* test).

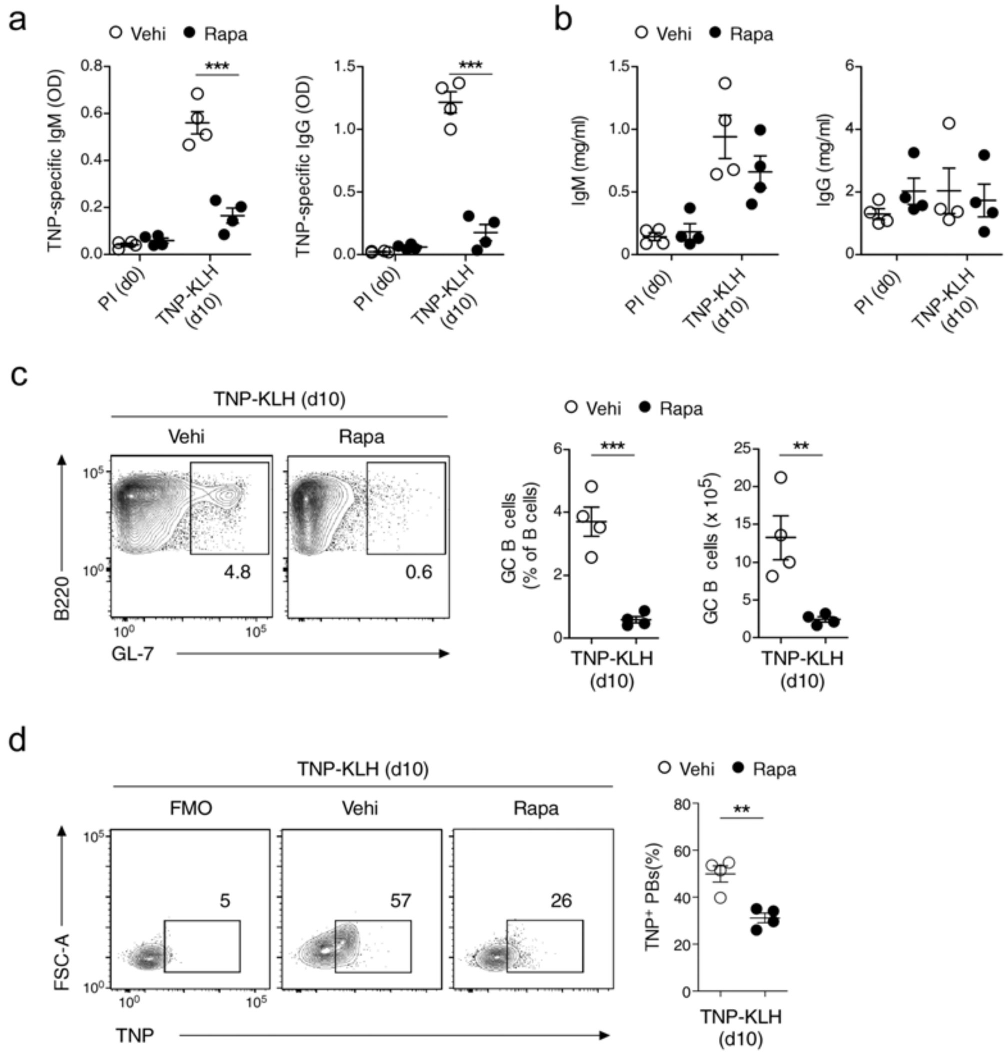


**Supplementary Figure 7. mTORC1 signaling is also required for FO B cell responses to TD antigens.** ELISA of serum TNP-specific (**a**) and total (**b**) IgM and IgG antibodies from pre-immune (PI) WT mice (d0) or post-immuune WT mice (d10) i.p. injected with TNP-keyhole limpet hemocyanin (KLH) and daily i.p. treated with control vehicle (vehi) or rapamycin (rapa). (**c**) FCM of GL-7$^+$ GC B cells in animals immunized as in (**a**). Numbers in dot plots indicate frequency of splenocytes, whereas frequency (right) and total GC B cell numbers (left) from different experiments are summarized in barplots. (**d**) FCM of TNP-binding PBs in animals immunized as in (**a**). Numbers in dot plots indicate frequency of PBs. Plots summarize data from two experiments with two animals per group. Error bars, s.e.m.; ∗∗ $p < 0.01$, ∗∗∗ $p < 0.001$ (two-tailed Student's t test).

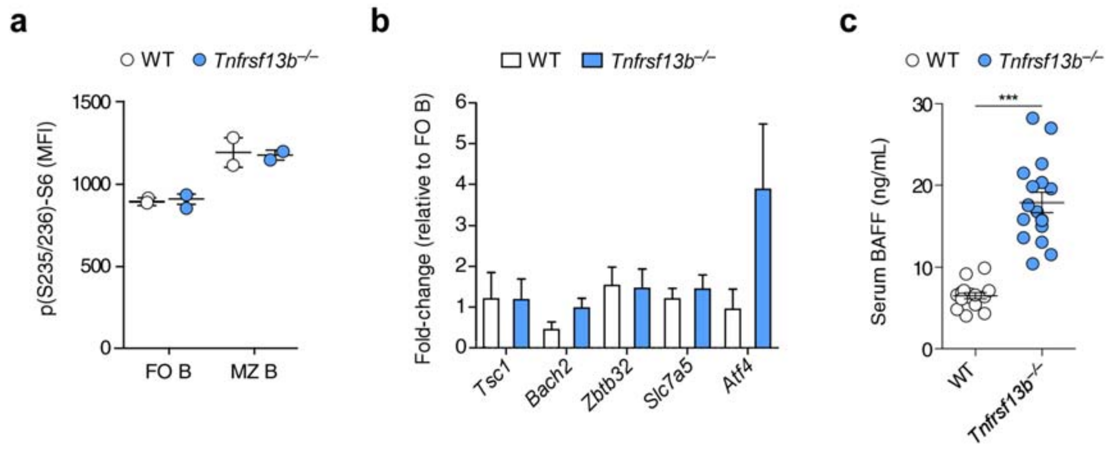


**Supplementary Figure 8. Mouse FO and MZ B cells show conserved mTORC1 signaling in the absence of TACI, possibly due to enhanced production of mTOR-inducing BAFF.** (**a**) Flow cytometry of p(S235/S236)-S6 in splenic FO and MZ B cells from WT (open bars) and *Tnfrsf13b*$^{-/-}$ (solid blue bars) mice. Cells were gated as in **Supplementary Fig. 11b.** (**b**) qRT-PCR of mRNAs for TSC1, BACH2, ZBTB32, SLC7A5 and ATF4 in MZ B cells from WT or *Tnfrsf13b*$^{-/-}$ mice. Results are normalized to mRNA for β-actin and presented as RE compared to a reference set of FO B cells. (**c**) Concentration of serum BAFF in WT (open bars) and *Tnfrsf13b*$^{-/-}$ (solid blue bars) mice. Data depict two (a), four (b) or at least 12 biological replicates for each strain (c). Error bars, s.e.m.; ∗∗∗ $p < 0.001$ (two-tailed Student's t test).

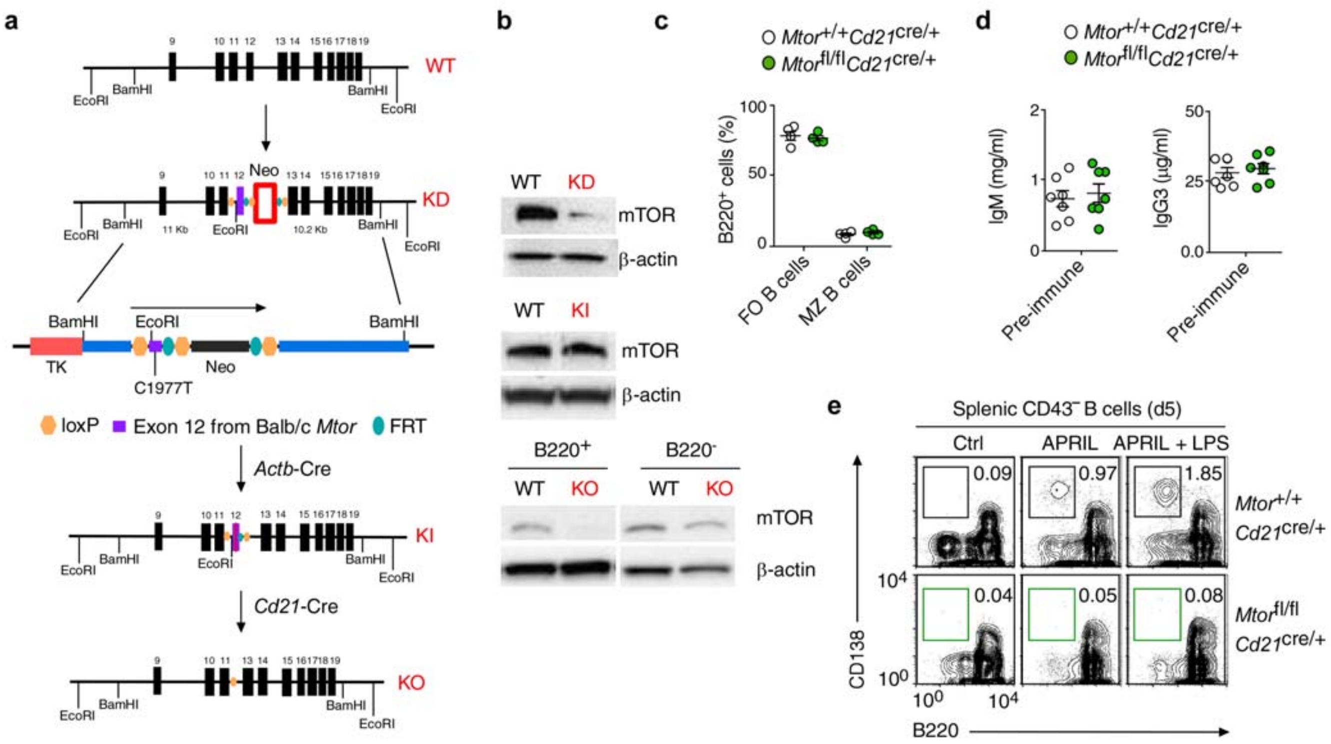


**Supplementary Figure 9. Strategy used to generate Cre-mediated mTOR deletion in mature mouse B cells.** (**a**) Schematic diagram of WT, knockdown (KD), knock-in (KI) and knockout (KO) *Mtor* alleles from C57BL/6 mice. In the KD allele, exon 12, which encodes part of the HEAT domain of mTOR, is replaced by a BALB/c-derived loxP-FRT-flanked exon 12. This floxed exon is followed by a loxP-FRT-flanked neomycin (neo) cassette located upstream of exon 13. KD mice carrying the neo-*Mtor* allele were bred with *Actb* (β-actin)-Cre mice and the resulting KI progeny showed ubiquitous deletion of the neomycin cassette (KI neo$^{-/-}$), but retained a functional *Mtor* allele. These *Mtor*$^{fl/fl}$ mice show mTOR expression and antigen-specific IgG responses comparable to those of WT mice [2, 3]. KO mice specifically lacking the *Mtor* allele in mature CD21$^{+}$ B cells were obtained by crossing *Mtor*$^{fl/fl}$ mice with *Cd21*-cre mice. (**b**) IB of mTOR in total splenocytes from WT, KD and KI animals and in B220$^{+}$ or control B220$^{-}$ cells from WT and KO mice. (**c,d**) FCM of splenic FO and MZ B cells and ELISA of total serum IgM and IgG3 from *Mtor*$^{+/+}$*Cd21*$^{cre/+}$ or *Mtor*$^{fl/fl}$ *Cd21*$^{cre/+}$ mice. (**e**) FCM of B220$^{low}$CD138$^{+}$ PBs induced by splenic resting CD43$^{-}$ B cells from *Mtor*$^{+/+}$*Cd21*$^{cre/+}$ or *Mtor*$^{fl/fl}$ *Cd21*$^{cre/+}$ mice following 5-d incubation

with medium alone (ctrl), APRIL or APRIL and LPS. Data show one representative experiment of at least three with similar results (**b,e**) or summarize at least two experiments with 3 mice per experimental group (**c,d**).

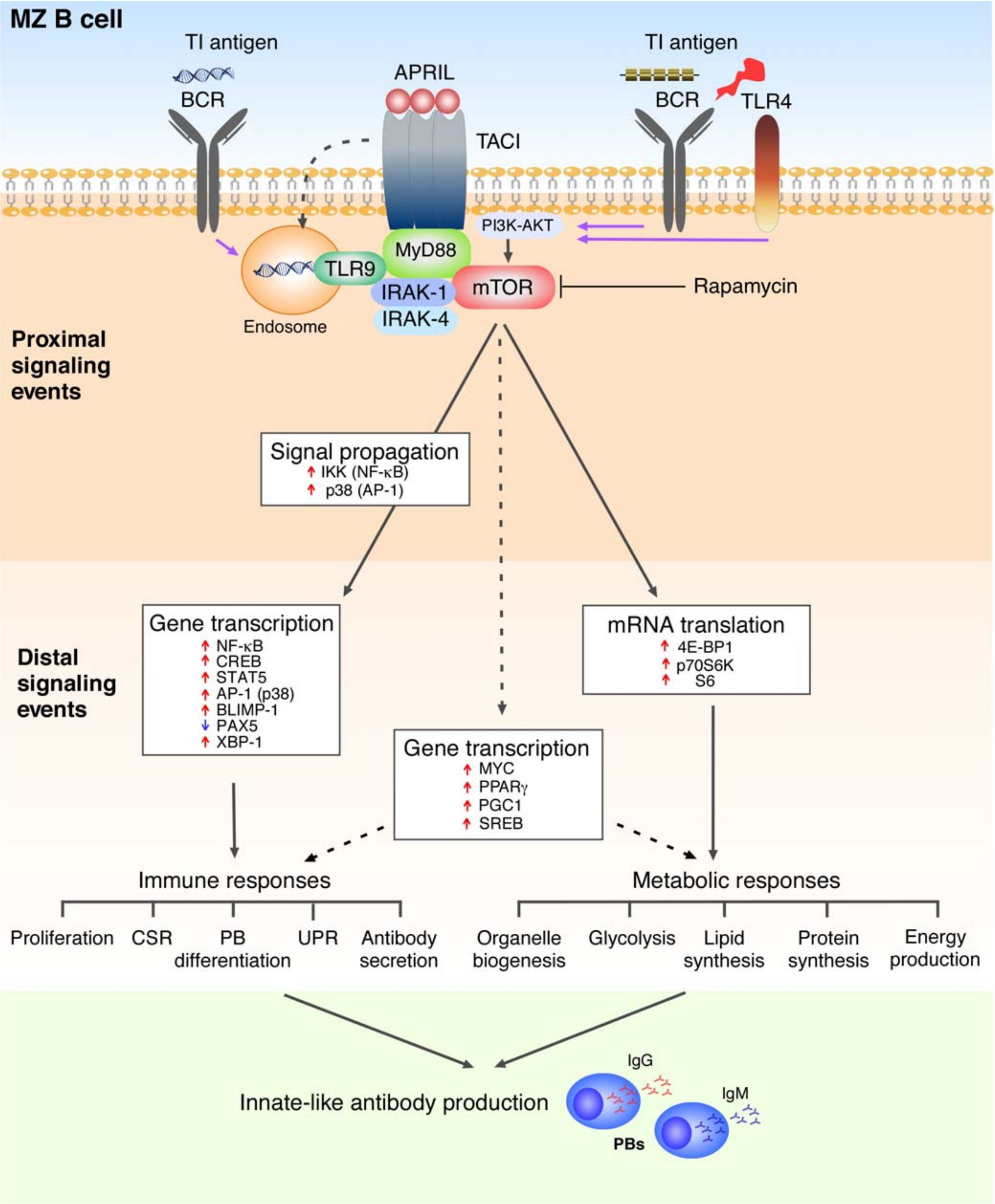


**Supplementary Figure 10. Model summarizing the proposed role of mTOR in the activation of MZ B cells by TACI.** Engagement of TACI by innate ligands such as APRIL triggers recruitment of the

metabolic rheostat mTOR to the cytoplasmic domain of TACI through the TLR adaptor protein MyD88. This event is associated with PI3K-AKT-regulated induction of rapamycin-sensitive mTOR signals that shape the pre-activated state of MZ B cells. TACI signalling via mTOR also drive MZ B cell responses to TI antigens in cooperation with signals from TLR4 or TLR9, which recognize conserved microbial structures often embedded in TI antigens. In particular, TACI may enhance cleavage-dependent signaling from TLR9 through an mTOR-dependent mechanism possibly involving ligation-induced internalization of TACI into TLR9-containing endosomes, followed by TACI association with cleaved TLR9. Besides signals from TLRs, the TACI-mTOR pathway likely intersects signals from BCR to elicit MZ B cell responses against TI antigens. These responses involve activation of coordinated immune and metabolic transcription programs in MZ B cells. (**a**) Immune transcription programs. mTOR signals from TACI induce canonical NF-κB transcription factors by activating IKK through a mechanism possibly involving MyD88-interacting IRAK-1 and IRAK4 kinases. mTOR signals from TACI further stimulate CREB, STAT5 and AP-1-inducing p38, which may cooperate with NF-κB to transcriptionally enhance proliferation, CSR and antibody production. Finally, mTORC1 signals from TACI transcriptionally stimulate PB differentiation and antibody secretion by activating a binary gene network coupling suppression of PAX5 with induction of BLIMP-1. This latter further promotes the UPR via XBP-1. (**b**) Metabolic transcription programs. mTOR signals from TACI elicit phosphorylation of 4E-BP1 and S6, a p70S6K-induced ribosomal protein required for mRNA translation and protein synthesis. TACI signalling via mTOR may further enhance organelle biogenesis, glycolysis, lipid synthesis and energy production by inducing transcription factors such as MYC, SREBP and PGC1, a co-factor of PPARγ. Dashed arrows: TACI-induced mTORC1 pathways needing further investigation; purple arrows: known BCR and TLR4 pathways.

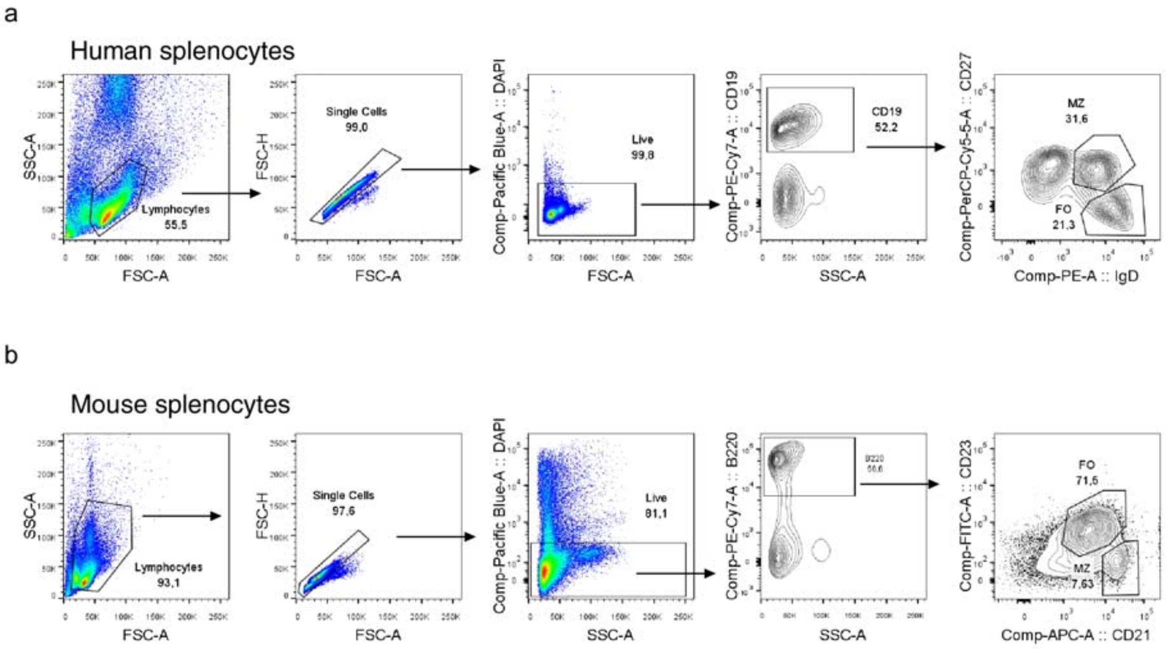


**Supplementary Figure 11. Gating strategies.** Gating strategies used to discriminate human (**a**) and mouse (**b**) splenic follicular (FO) and marginal zone (MZ) B cell subsets.

**Supplementary Figure 12.** Scanned original full length images of gels and immunoblots.

## Fig. 2d

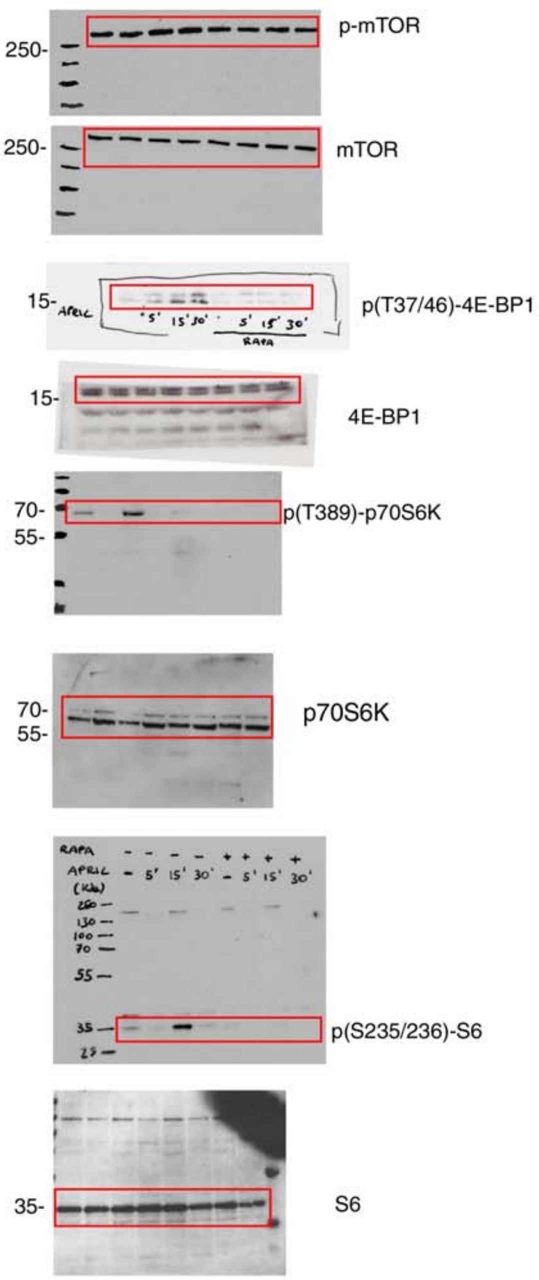


## Fig. 2e

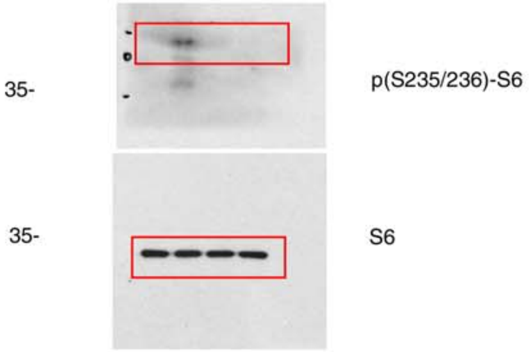


## Fig. 2g

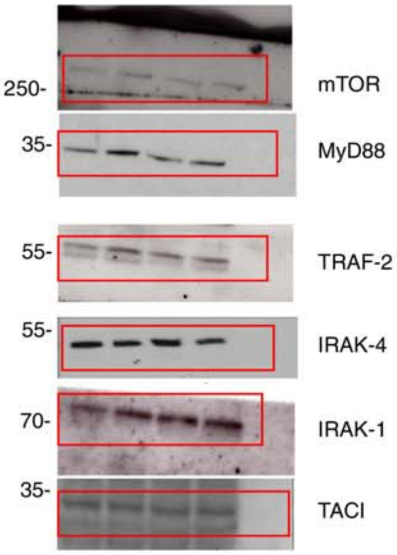


## Fig. 2i

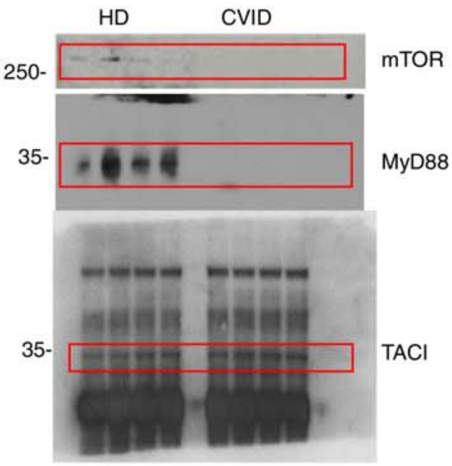

## Fig. 2f

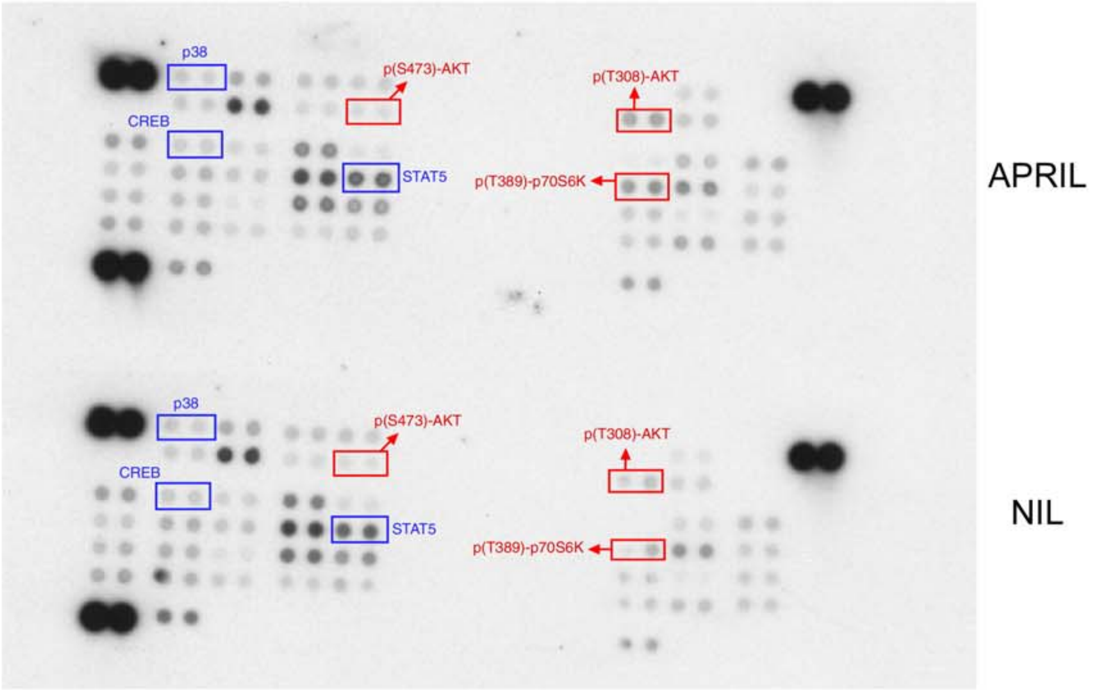


## Fig. 3a

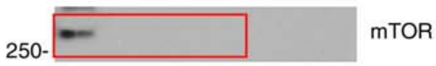


## Fig. 3c

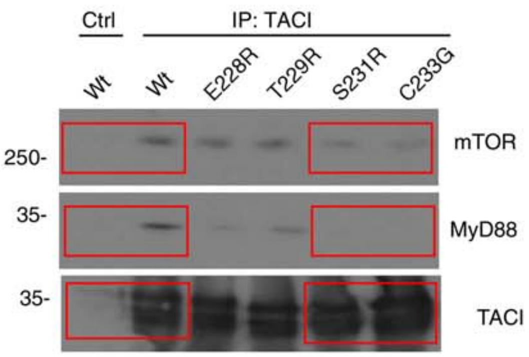


## Fig. 3d

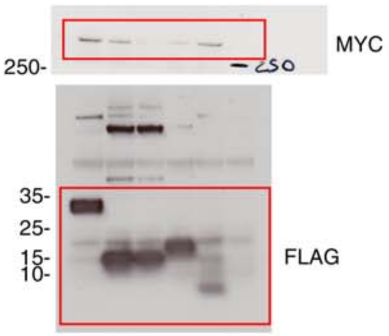


## Fig. 3f

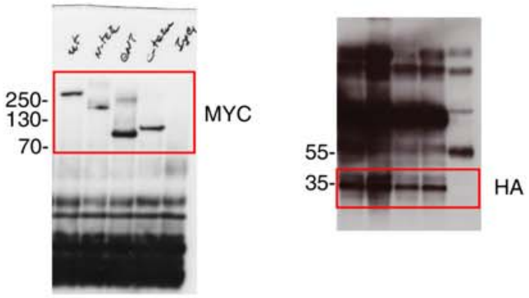

## Fig 4b

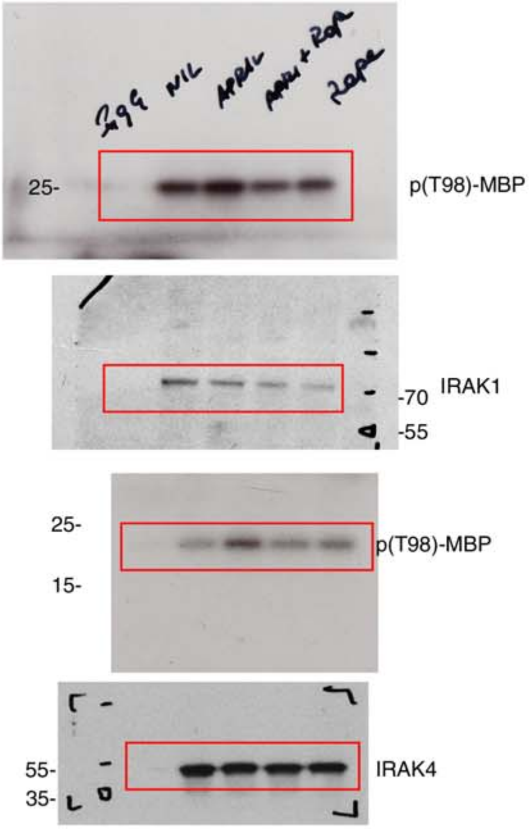


## Fig 4c

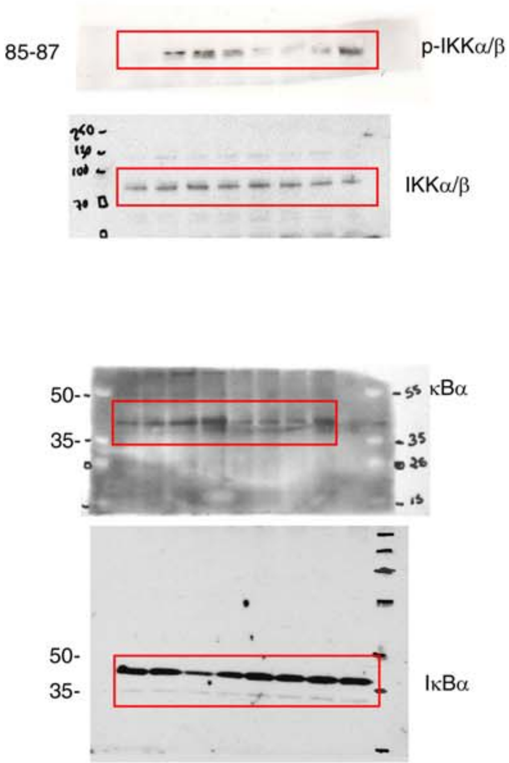


## Fig 4d

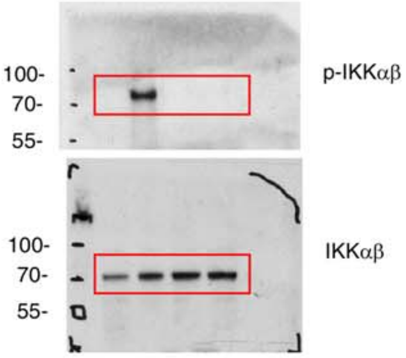


## Fig 4e

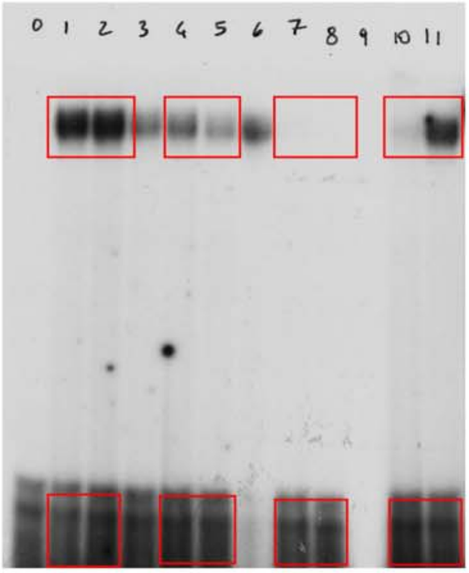

## Fig 5a

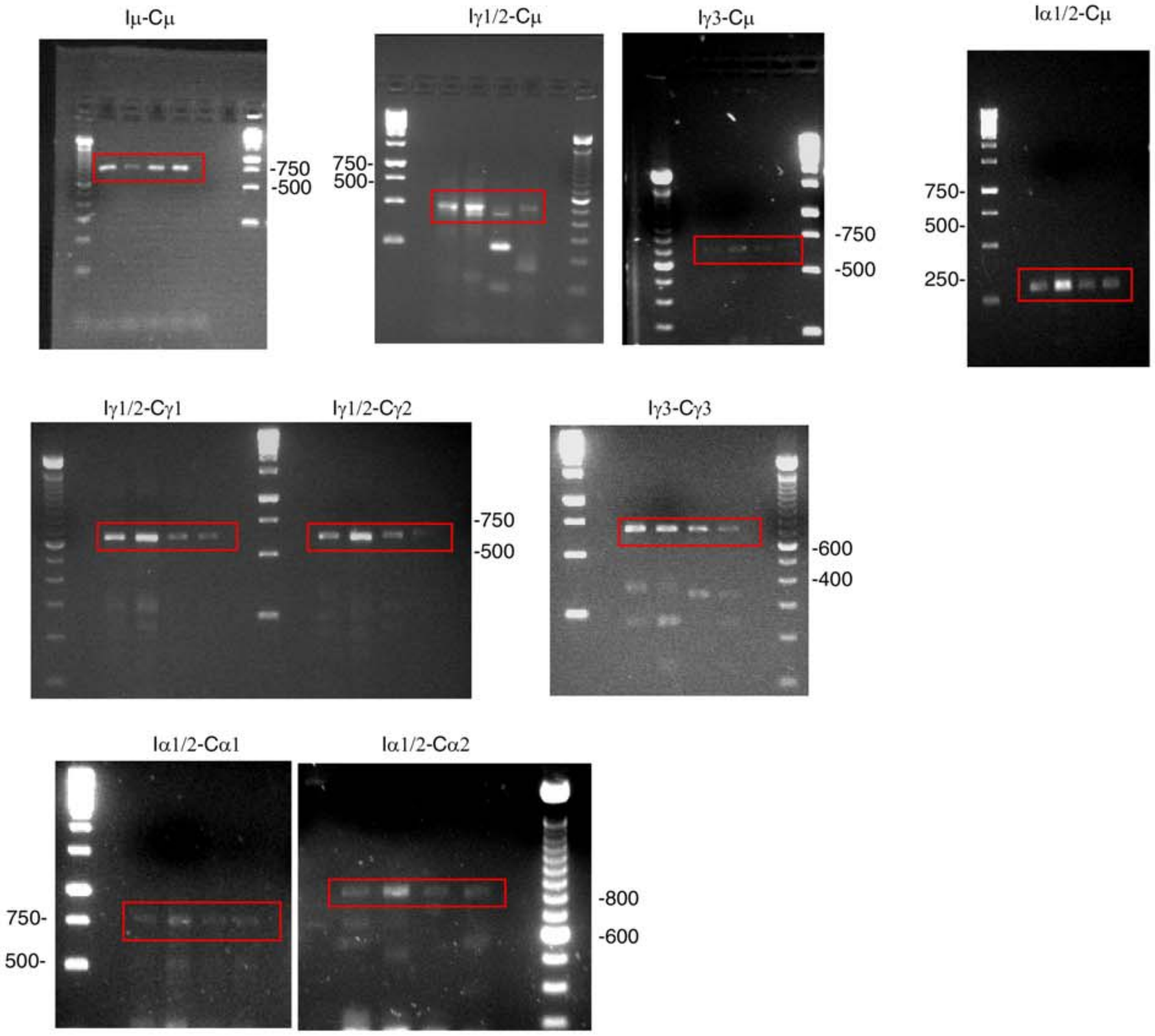
Iμ-Cμ
-750
-500
Iγ1/2-Cμ
750-
500-
Iγ3-Cμ
-750
-500
Iα1/2-Cμ
750-
500-
250-
Iγ1/2-Cγ1
Iγ1/2-Cγ2
-750
-500
Iγ3-Cγ3
-600
-400
Iα1/2-Cα1
750-
500-
Iα1/2-Cα2
-800
-600

## Fig 5c

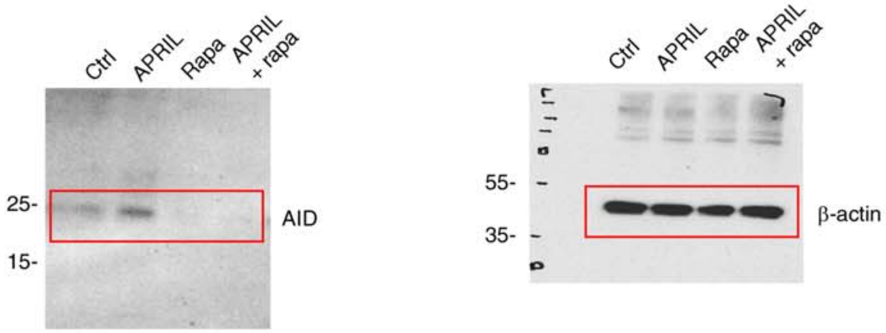
Ctrl
APRIL
Rapa
APRIL
+ rapa
25-
15-
AID
Ctrl
APRIL
Rapa
APRIL
+ rapa
55-
35-
β-actin

Fig 7e

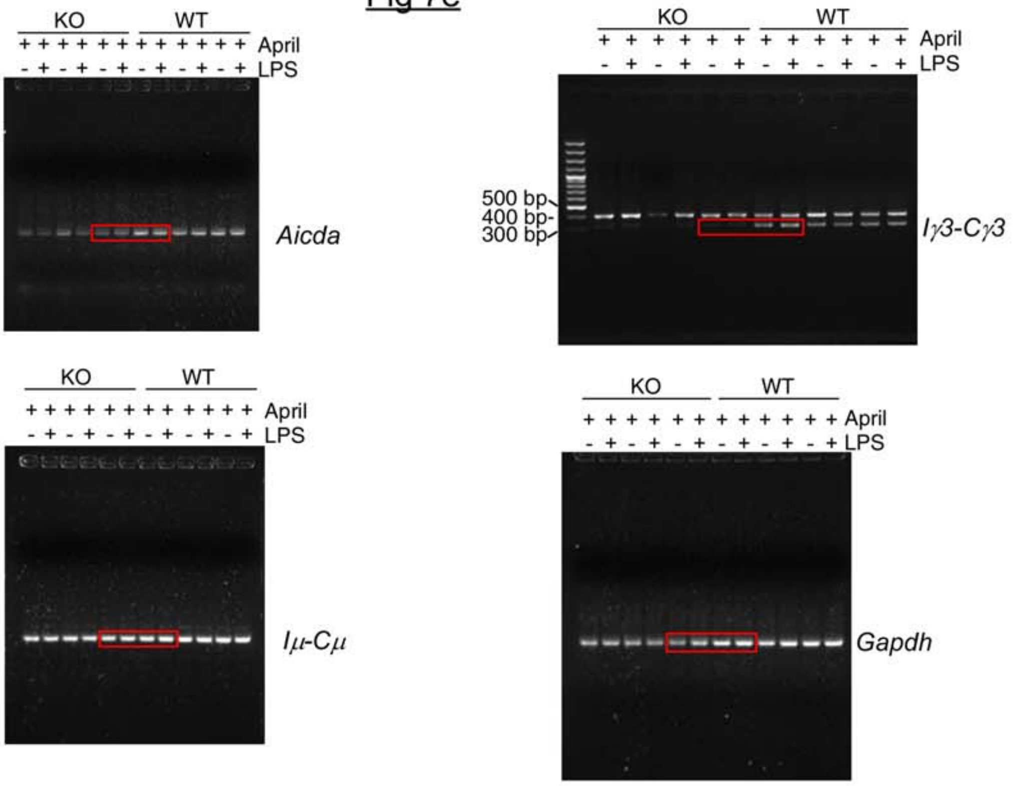

<u>Supl. Fig 7b</u>

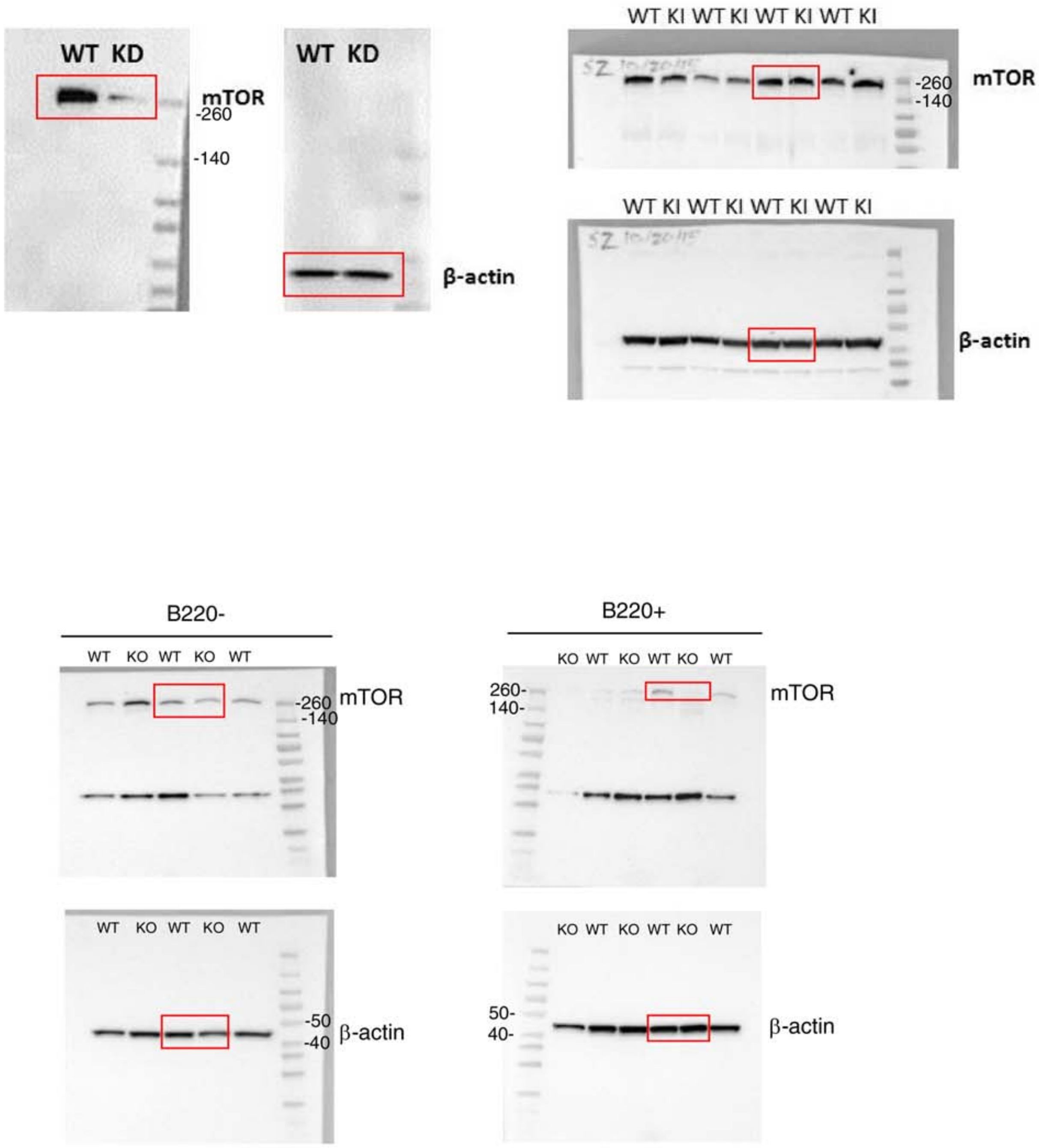

## Supplementary Tables

**Supplementary Table 1. Genes with highest degree of differential expression in human splenic MZ B cells exposed to APRIL or rapamycin.**

| APRIL vs unstimulated | | | Rapamycin vs unstimulated | | | Rapamycin vs APRIL | | |
|---|---|---|---|---|---|---|---|---|
| **Gene symbol** | **FC** | **BP** | **Gene symbol** | **FC** | **BP** | **Gene symbol** | **FC** | **BP** |
| *CCL17* | 2,39 | *a* | *AK055942* | 1,68 | *l* | *IGF1R* | 1,73 | *a* |
| *PLA2G4C* | 1,77 | *b* | *KLF17* | 1,66 | *f* | *GOLGA6L9* | 1,70 | *l* |
| *CBLN3* | 1,70 | *c* | *ABCA9* | 1,62 | *b* | *TBC1D9* | 1,69 | *e* |
| *NOD2* | 1,70 | *a,d* | *M15530* | 1,59 | *l* | *MLL3* | 1,67 | *k* |
| *TLCD1* | 1,67 | *e* | *PCNA* | -1,52 | *h* | *TRAF4* | -1,93 | *a,d* |
| *ZMIZ2* | 1,63 | *f* | *LAP3* | -1,52 | *a* | *MTHFD2* | -1,94 | *h,m,o* |
| *SYNPO* | 1,62 | *g,i* | *FASN* | -1,54 | *b,m* | *FSCN1* | -1,95 | *h* |
| *EBI3* | 1,62 | *a,i* | *PRDX3* | -1,54 | *h,n* | *ICAM1* | -1,99 | *a,d* |
| *TNFAIP3* | 1,59 | *a* | *CD1C* | -1,55 | *a* | *NOD2* | -2,01 | *a,d* |
| *TRAF4* | 1,56 | *d* | *DDIT4* | -1,56 | *b,m,o* | *SYNPO* | -2,05 | *g,i* |
| *CYB5R2* | 1,51 | *b* | *CHEK1* | -1,60 | *h* | *NINJ1* | -2,05 | *d* |
| *TESC* | 1,51 | *e* | *ISOC2* | -1,61 | *l* | *PAICS* | -2,13 | *h* |
| *CCDC28B* | 1,47 | *j* | *IFIT1* | -1,62 | *a,o* | *CDC45* | -2,15 | *h* |
| *SH3PXD2A* | 1,46 | *a* | *PHGDH* | -1,65 | *m* | *PLXNA1* | -2,17 | *g* |
| *CLEC12A* | 1,45 | *a* | *CCR1* | -1,68 | *i* | *BATF3* | -2,24 | *a,p* |
| *C17orf49* | 1,43 | *k* | *CKS2* | -1,74 | *h* | *TLCD1* | -2,33 | *e* |
| *STARD10* | 1,42 | *b* | *TNF* | -1,75 | *a,d,i* | *DNASE1L3* | -2,48 | *l* |
| *SLC45A3* | 1,40 | *b* | *DNASE1L3* | -1,94 | *l* | *CCL4* | -2,51 | *a,d* |
| *PASK* | 1,40 | *b,c* | *CCL4* | -2,56 | *a,d* | *CCL17* | -2,77 | *a* |
| *STEAP1* | 1,39 | *l* | *C1QTNF5* | -2,68 | *l* | *CCL22* | -2,97 | *a* |

BP, biological process.

Category annotation was performed by GSEA using the Molecular Signature Data Base (MSigDB) at: http://software.broadinstitute.org/gsea/msigdb/search.jsp

*a* Activation of immune responses
*b* Lipid and glucose metabolic processes
*c* Protein secretion
*d* NF-κB signaling
*e* Calcium transport
*f* Regulation of gene transcription
*g* Cytoskeleton organization
*h* Cell proliferation
*i* IL-6/STAT3 signaling
*j* Organelle assembly
*k* DNA metabolic process
*l* Other
*m* mTORC1 signaling
*n* Oxidative phosphorylation
*o* UPR
*p* STAT5 signaling

**Supplementary Table 2. Antibodies to human antigens.**

| Antigen | Label | Isotype | Clone | Manufacturer | Use |
|---|---|---|---|---|---|
| CD19 | PE-Cy7[f] | m°,IgG1,κ | HIB19 | Biolegend | FCM |
| CD27 | PerCp-Cy5.5[⌡] | m,IgG1,κ | M-T271 | BD | FCM |
| CD38 | APC[^] | m,IgG1,κ | HIT2 | BD | FCM |
| TACI | PE | m,IgG2a,κ | 11H3 | eBioscience | FCM |
| BCMA | PE | Goat, IgG | FAB193P | R&D | FCM |
| CD98 | PE | m,IgG1,κ | UM7F8 | BD | FCM |
| CD45 | Alexa 700 | m,IgG1,κ | HI30 | BioLegend | FCM |
| BLIMP-1 | PE | m,IgG1,κ | 646702 | R&D | FCM |
| XBP-1s | PE | m,IgG1,κ | Q3-695 | BD | FCM |
| IgD | FITC[‡] | Goat, IgG | 2032-02 | Southern | FCM |
| IgD | Biotin | Goat, IgG | 2032-08 | Southern | IFA |
| p-(S235/236)S6 | PE | Rb*, IgG | D57.2.2E | Cell Signalling | FCM/IFA |
| p-(T308)AKT | PE | Rb, IgG | D25E6 | Cell Signalling | FCM |
| p-(S473)AKT | PE | Rb, IgG | D9E | Cell Signalling | FCM |
| MAdCAM-1 | Unlabeled | m,IgG1 | 314G8 | AbD Serotech | IFA |
| β-actin | Unlabeled | m,IgG1,κ | AC-15 | Santa Cruz | IB |
| TLR9 | Unlabeled | m,IgG1,κ | 26C593.2 | Thermo Fischer | IB |
| TACI | Unlabeled | Goat IgG | ab109852 | Abcam | IB/IP |
| MyD88 | Unlabeled | Rb, IgG | ab2068 | Abcam | IB |
| TRAF2 | Unlabeled | Rb, IgG | sc-876 | Santa Cruz | IB |
| IRAK1 | Unlabeled | Rb, IgG | D51G7 | Cell Signalling | IB |
| p-(T387)IRAK-1 | Unlabeled | Rb, IgG | 4365 | Cell Signalling | IB/IP |
| IRAK4 | Unlabeled | Rb, IgG | 4363 | Cell Signalling | IB |
| p-(T345/S346) IRAK4 | Unlabeled | Rb, IgG | D6D7 | Cell Signalling | IB/IP |
| mTOR | Unlabeled | Rb, IgG | 2972 | Cell Signalling | IB |
| p-(S2448)mTOR | Unlabeled | Rb, IgG | 2971 | Cell Signalling | IB |
| p70S6K | Unlabeled | Rb, IgG | 9202 | Cell Signalling | IB |
| p-(T389)p70S6K | Unlabeled | Rb, IgG | 9205 | Cell Signalling | IB |
| S6K | Unlabeled | Rb, IgG | 5G10 | Cell Signalling | IB |
| p-(S235/236)S6K | Unlabeled | Rb, IgG | 2F9 | Cell Signalling | IB |
| 4E-BP1 | Unlabeled | Rb, IgG | 53H11 | Cell Signalling | IB |
| p-(T37/46)4E-BP1 | Unlabeled | Rb, IgG | 236B4 | Cell Signalling | IB |
| IKKα/β | Unlabeled | Rb, IgG | sc-7607 | Santa Cruz | IB |
| p-(S176/180) IKKα/β | Unlabeled | Rb, IgG | 16A6 | Cell Signalling | IB |
| IκBα | Unlabeled | m,IgG1 | L35A5 | Cell Signalling | IB |
| p-(S32/36) IκBα | Unlabeled | m,IgG1 | 5A5 | Cell Signalling | IB |
| p-(T98) MBP | Unlabeled | m,IgG | P12 | Millipore | IB |
| p-(S792) RAPTOR | Unlabeled | Rb, IgG | 2083 | Cell Signalling | IB/FCM |
| ATF4 | Unlabeled | Rb, IgG | D4B8 | Cell Signalling | FCM |
| NFκB p65 | APC | m,IgG2a,κ | 14G10A21 | BioLegend | FCM |
| NFκB p65 | Unlabeled | Rb, IgG | sc-293111 | Santa Cruz | IB/IFA |
| NFκB p50 | Unlabeled | Rb, IgG | sc-114 | Santa Cruz | IB/EMSA/IFA |
| NFκB p52 | Unlabeled | Rb, IgG | sc-848 | Santa Cruz | IB/EMSA/IFA |
| Histidine | Unlabeled | m,IgG1 | 27E8 | Cell Signalling | IB/IP |
| Myc | Unlabeled | m,IgG1 | Myc.A7 | Abcam | IB |
| FLAG | Unlabeled | Rb, IgG | F7425 | Sigma | IB |
| HA | Unlabeled | m,IgG1 | HA-7 | Sigma | IB |
| Isotype IgG (m) | Unlabeled | m,IgG | sc-2025 | Santa Cruz | Isotype |

| | | | | | |
|---|---|---|---|---|---|
| Isotype IgG (Rb) | Unlabeled | Rb, IgG | sc-2028 | Santa Cruz | Isotype |

[f]PE and Cy, phycoerythrin and cyanin
[o]m, mouse
[∫] PerCP, peridinin chlorophyll
[^]APC, allophycocyanin
[‡]FITC, fluorescein
*Rb, rabbit

**Supplementary Table 3. Antibodies to mouse antigens.**

| Antigen | Label | Isotype | Clone | Manufacturer | Use |
|---|---|---|---|---|---|
| CD21/CD35 | APC | Rat IgG2b,κ | 7G6 | BD | FCM |
| CD23 | FITC | Rat IgG2a,κ | B3B5 | eBioscience | FCM |
| CD43 | APC-Cy7 | Rat IgG2a,κ | 1B11 | BioLegend | FCM |
| B220 | PE-Cy7 | Rat IgG2a,κ | RA3-6B2 | BD | FCM |
| CD138 | APC | Rat IgG2a,κ | 281-2 | BD | FCM |
| TACI | PE | Rat IgG2a,κ | 8F10 | BioLegend | FCM |
| BCMA | FITC | Rat IgG1 | 161616 | R&D | FCM |
| IgG3 | PE | Rat IgG2a,λ | SB76b | Southern | FCM |
| IgM | FITC | Rat IgG2a,κ | RMM-1 | BioLegend | FCM |
| IgM | Biotin | Goat F(ab')2 | 1022-08 | Southern | IFA/ELISA |
| Ki-67 | FITC | Rat IgG2a,κ | 16A8 | eBioscience | FCM |
| p(S235/236)-S6 | PE | Rabbit IgG | D57.2.2E | Cell Signalling | FCM/IFA |
| MOMA-1 | Biotin | Rat IgG2a,κ | MOMA-1 | Abcam | IFA |
| IgG | Biotin | Goat IgG | 1033-08 | Southern | ELISA |
| IgM | Biotin | Rat IgG2a,κ | II/41 | BD | ELISA |
| IgG3 | Biotin | Rat IgG2a,κ | R40-82 | BD | ELISA |
| mTOR | Unlabeled | Rb, IgG | 2972 | Cell Signalling | IB |
| β-actin | Unlabeled | Rb, IgG | 13E5 | Cell Signalling | IB |

**Supplementary Table 4. Primers for human gene products.**

| **Target gene** | | **Primer sequence** | **Use** |
|---|---|---|---|
| *ACTB* | S[^] | GGATGCAGAAGGAGATCACT | qRT-PCR |
| | AS[#] | CGATCCACACGGAGTACTTG | qRT-PCR |
| *AICDA* | S | AGAGGCGTGACAGTGCTACA | qRT-PCR |
| | AS | TGTAGCGGAGGAAGAGCAAT | qRT-PCR |
| *PAX5* | S | TTGCTCATCAAGGTGTCAGG | qRT-PCR |
| | AS | CTGATCTCCCAGGCAAACAT | qRT-PCR |
| *PRDM1* | S | GTGGTATTGTCGGGACTTTGCAG | qRT-PCR |
| | AS | TCGGTTGCTTTAGACTGCTCTGTG | qRT-PCR |
| *XBP1* | S | AGGAGTTAAGACAGCGCTTGG | qRT-PCR |
| | AS | AGAGGTGCACGTAGTCTGAGTGCTG | qRT-PCR |
| *TNFRSF13B* | S | CAGACAACTCGGGAAGGTACC | qRT-PCR |
| | AS | GCCACCTGATCTGCACTCAGCTTC | qRT-PCR |
| *TLR9* | S | ACAACAACATCCACAGCCAAGTGTC | qRT-PCR |
| | AS | AAGGCCAGGTAATTGTCACGGAG | qRT-PCR |
| *BACH2* | S | CCAGCAATGACTCAGGCATC | qRT-PCR |
| | AS | TCATGAGTCTTGTCGCTGGT | qRT-PCR |
| *FOXO1* | S | TTATGACCGAACAGGATGATCTTG | qRT-PCR |
| | AS | TGTTGGTGATGAGAGAAGGTTGAG | qRT-PCR |
| *ZBTB32* | S | GAGTGCAGTCCCTGGAAGAG | qRT-PCR |
| | AS | TCTCTGGCTCCTCCTGATGT | qRT-PCR |
| *AKT1* | S | TCTATGGCGCTGAGATTGTG | qRT-PCR |
| | AS | CTTAATGTGCCCGTCCTTGT | qRT-PCR |
| *MTOR* | S | TTGCTTGAGGTGCTACTG | qRT-PCR |
| | AS | CTGACTTGACTTGGATTCTG | qRT-PCR |
| *DEPTOR* | S | CACCATGTGTGTGATGAGCA | qRT-PCR |
| | AS | TGAAGGTGCGCTCATACTTG | qRT-PCR |
| *RPTOR* | S | ACTGATGGAGTCCGAAATGC | qRT-PCR |
| | AS | TCATCCGATCCTTCATCCTC | qRT-PCR |
| *RICTOR* | S | GGAAGCCTGTTGATGGTGAT | qRT-PCR |
| | AS | GGCAGCCTGTTTTATGGTGT | qRT-PCR |
| *MYD88* | S | GAGCGTTTCGATGCCTTCAT | qRT-PCR |
| | AS | CGGATCATCTCCTGCACAAA | qRT-PCR |
| *TSC1* | S | CCGAGGAGCCCGAAGTTCT | qRT-PCR |
| | AS | TCATTTGCTCTGCGTCTTCC | qRT-PCR |
| *FABP5* | S | CCTGTCCAAAGTGATGATGG | qRT-PCR |
| | AS | CAGCCATCAGGAGTGGGATG | qRT-PCR |
| *PYGB* | S | GACGAGGTGTTCATCAGGGA | qRT-PCR |
| | AS | ATTGTACAGGGTGACGACGT | qRT-PCR |
| Iμ | S | GTGATTAAGGAGAAACACTTTGAT | PCR |
| Cμ 243 | AS | CACACCACGTGTTCGTCTG | PCR |
| Cμ 268 | AS | GTTGCCGTTGGGGTGCTGGAC | PCR |
| Iα 1/2 | S | CAGCAGCCCTCTTGGCAGGCAGCCAG | PCR |
| Iα internal | S | CTCAGCACTGCG GGCCCTCCA | PCR |
| Cα1 | AS | GGGTGGCGGTTAGCGGGGTCTTGG | PCR |
| Cα2 | AS | TGTTGGCGGTTAGTGGGGTCTTCA | PCR |
| Iγ 1/2 | S | GGGCTTCCAAGCCAACAGGGCAGGACA | PCR |
| Iγ internal | S | GACCTGAGCTCAGGAGGCAGCAGAGCGA | PCR |
| Iγ3 | S | AGGTGGGCAGCCTTCAGGCACCGAT | PCR |

| | | | |
|---|---|---|---|
| Cγ1 | AS | GTTTTGTCACAAGATTTGGGCTC | PCR |
| Cγ2 | AS | GTGGGCACTCGACACAACATTTGCG | PCR |
| Cγ3 | AS | TTGTGTCACCAAGTGGGGTTTTGAGC | PCR |

[^]S, sense primer
[#]AS, antisense primer

**Supplementary Table 5. Primers for mouse gene products.**

| Target gene | | Primer sequence | Use |
|---|---|---|---|
| 18S rRNA | S^ | GCCGCTAGAGGTGAAATTCTTG | qRT-PCR |
| | AS# | CATTCTTGGCAAATGCTTTCG | qRT-PCR |
| *Aicda* | S | GCCACCTTCGCAACAAGTCT | qRT-PCR |
| | AS | CCGGGCACAGTCATAGCAC | qRT-PCR |
| *Xbp1* | S | ACATCTTCCCATGGACTCTG | qRT-PCR |
| | AS | TAGGTCCTTCTGGGTAGACC | qRT-PCR |
| *Prdm1* | S | GGAGAGGCTCCACTACCCTT | qRT-PCR |
| | AS | GCTTTGGGTTGCTTTCCGTT | qRT-PCR |
| Iμ | S | CTCTGGCCCTGCTTATTGTTG | PCR |
| Iγ3 | S | TGGGCAAGTGGATCTGAACA | PCR |
| Cμ | AS | GAAGACATTTGGGAAGGACTGACT | PCR |
| Cγ3 | AS | CTCAGGGAAGTAGCCTTTGACA | PCR |

**Supplementary Table 6. Human constructs.**

| Construct | Plasmid | Manufacturer | Tag | Residues |
|---|---|---|---|---|
| TACI WT | pCDNA3 | Cerutti lab | His | 1-293 |
| TACI D1 | pCDNA3 | Cerutti lab | His | 1-226 |
| TACI D2 | pCDNA3 | Cerutti lab | His | 1-216 |
| TACI-mCherry | pCI-neo | Cunningham-Rundles lab | mCherry | 1-293 |
| MyD88 WT | pCMV-Flag | Cerutti lab | HA | 1-296 |
| MyD88 WT | pCMV-Flag | Cerutti lab | FLAG | 1-296 |
| MyD88 D1 | pCMV-Flag | Cerutti lab | FLAG | 1-109 |
| MyD88 D2 | pCMV-Flag | Cerutti lab | FLAG | 1-160 |
| MyD88 D3 | pCMV-Flag | Cerutti lab | FLAG | 109-296 |
| MyD88 D4 | pCMV-Flag | Cerutti lab | FLAG | 160-296 |
| mTOR WT | pCDNA3.1A | Addgene | MYC | 1-2549 |
| mTOR D1 | pCDNA3.1A | Addgene | MYC | 1-1482 |
| mTOR D2 | pCDNA3.1A | Addgene | MYC | 1271-2000 |
| mTOR D3 | pCDNA3.1A | Addgene | MYC | 1750-2549 |
| TSC1 | pCDNA3.1 | Addgene | MYC | 1-1164 |
| TSC2 | pCDNA3.1 | Addgene | MYC | 1-1807 |
| TLR9-eYFP | pCDNA3 | Addgene | eYFP | 1-1032 |
| NF-κB | pGL3 | Promega | Luciferase | - |

**Supplementary Table 7. Primers for Human In-Fusion Cloning.**

| Target Gene | | Primer sequence |
|---|---|---|
| TACI cassette | S | CATTGGCTCGAGATGCCCATGGGGTC |
| | AS | CCAGTCAAGCTTTCTGCACCTGGGCC |
| mCherry | S | GGTAAGCTTGATGGTGAGCAAGG |
| | AS | CAAGCGGCCGCCTACTTGTACAG |
| TACI-mCherry | S | GACTCACTATAGGCTAGCCTCGAGATGCCCATGGGG |
| | AS | CTAAAGGGAAGCGGCCGCCTACTTGTACAGCTCGTC |
