# Supplementary material for: mTOR intersects antibody-inducing signals from TACI in marginal zone B cells
Source: Nat Commun. 2017 Nov 13;8:1462. doi: 10.1038/s41467-017-01602-4 (PMC5684130; doi:10.1038/s41467-017-01602-4)
Supplement: Supplementary file 3 — Description of Additional Supplementary Files [file 41467_2017_1602_MOESM3_ESM.pdf]

Description of Additional Supplementary Files

File Name: Supplementary Data 1
Description: Genes with highest degree of differential expression in human splenic MZ B cells compared to naïve follicular B cells.
