# Supplementary material for: mTOR intersects antibody-inducing signals from TACI in marginal zone B cells
Source: Nat Commun. 2017 Nov 13;8:1462. doi: 10.1038/s41467-017-01602-4 (PMC5684130; doi:10.1038/s41467-017-01602-4)
Supplement: Supplementary file 4 — Supplementary Data 1 [file 41467_2017_1602_MOESM4_ESM.docx]

**Supplementary Data 1. Genes with highest degree of differential expression in human splenic MZ B cells compared to naïve follicular B cells.**

| **Gene symbol** | **Entrez gene name** | **FC** | **FDR** | **Cellular location** |
| --- | --- | --- | --- | --- |
| *CD27* | CD27 molecule | 12.58 | 0.000 | Plasma membrane |
| *TESC* | tescalcin | 9.89 | 0.000 | Cytoplasm |
| *TMEM132A* | transmembrane protein 132A | 9.38 | 0.003 | Cytoplasm |
| *FCRL4* | Fc receptor like 4 | 7.85 | 0.021 | Cytoplasm |
| *CLECL1* | C-type lectin like 1 | 7.55 | 0.000 | Plasma membrane |
| *KLK1* | kallikrein 1 | 7.29 | 0.000 | Cytoplasm |
| *LINC00152* | long intergenic non-protein coding RNA 152 | 7.23 | 0.000 | Other |
| *GNG11* | G protein subunit gamma 11 | 7.12 | 0.044 | Plasma membrane |
| *MIR155HG* | MIR155 host gene | 7.00 | 0.007 | Other |
| *CYB5R2* | cytochrome b5 reductase 2 | 6.90 | 0.000 | Cytoplasm |
| *BHLHE41* | basic helix-loop-helix family member e41 | 6.74 | 0.000 | Nucleus |
| *PDCD1* | programmed cell death 1 | 6.52 | 0.003 | Plasma membrane |
| *TYMS* | thymidylate synthetase | 6.29 | 0.027 | Nucleus |
| *ZBTB32* | zinc finger and BTB domain containing 32 | 6.26 | 0.001 | Nucleus |
| *MAST4* | microtubule associated serine/threonine kinase family member 4 | 6.21 | 0.000 | Other |
| *CCL3* | C-C motif chemokine ligand 3 | 6.09 | 0.022 | Extracellular Space |
| *TNF* | tumor necrosis factor | 6.03 | 0.000 | Extracellular Space |
| *AIM2* | absent in melanoma 2 | 5.95 | 0.004 | Cytoplasm |
| *TNFRSF13B* | tumor necrosis factor receptor superfamily member 13B | 5.87 | 0.005 | Plasma membrane |
| *FABP5* | fatty acid binding protein 5 | 5.85 | 0.000 | Cytoplasm |
| *TCF7* | transcription factor 7 (T-cell specific, HMG-box) | 5.82 | 0.001 | Nucleus |
| *CCL4* | C-C motif chemokine ligand 4 | 5.48 | 0.024 | Extracellular Space |
| *SEMA7A* | semaphorin 7A (John Milton Hagen blood group) | 5.44 | 0.009 | Plasma membrane |
| *MARCKS* | myristoylated alanine rich protein kinase C substrate | 5.40 | 0.000 | Plasma membrane |
| *MTHFD2* | methylenetetrahydrofolate dehydrogenase (NADP+ dependent) 2 | 5.28 | 0.002 | Cytoplasm |
| *LOC284191* | uncharacterized LOC284191 | 5.16 | 0.000 | Other |
| *TFPI2* | tissue factor pathway inhibitor 2 | 4.75 | 0.000 | Extracellular Space |
| *CALHM3* | calcium homeostasis modulator 3 | 4.74 | 0.000 | Extracellular Space |
| *KIAA0101* | KIAA0101 | 4.74 | 0.041 | Nucleus |
| *PLA2G16* | phospholipase A2 group XVI | 4.73 | 0.046 | Nucleus |
| *DOK3* | docking protein 3 | 4.72 | 0.000 | Cytoplasm |
| *LGALS1* | lectin, galactoside binding soluble 1 | 4.64 | 0.000 | Extracellular Space |
| *DPP4* | dipeptidyl peptidase 4 | 4.62 | 0.003 | Plasma membrane |
| *DNASE1L3* | deoxyribonuclease I like 3 | 4.60 | 0.002 | Nucleus |
| *TLCD1* | TLC domain containing 1 | 4.51 | 0.000 | Other |
| *COL4A4* | collagen type IV alpha 4 | 4.46 | 0.000 | Extracellular Space |
| *CD70* | CD70 molecule | 4.37 | 0.000 | Extracellular Space |
| *NINJ1* | ninjurin 1 | 4.35 | 0.008 | Plasma membrane |
| *RHOBTB3* | Rho related BTB domain containing 3 | 4.34 | 0.013 | Cytoplasm |
| *TNFSF9* | tumor necrosis factor superfamily member 9 | 4.20 | 0.000 | Plasma membrane |
| *DUSP2* | dual specificity phosphatase 2 | 4.18 | 0.000 | Nucleus |
| *MDFIC* | MyoD family inhibitor domain containing | 4.11 | 0.000 | Nucleus |
| *C3orf14* | chromosome 3 open reading frame 14 | 4.10 | 0.001 | Other |
| *CCL22* | C-C motif chemokine ligand 22 | 4.07 | 0.040 | Extracellular Space |
| *HCST* | hematopoietic cell signal transducer | 4.07 | 0.006 | Plasma membrane |
| *LGALS3* | lectin, galactoside binding soluble 3 | 4.05 | 0.004 | Extracellular Space |
| *TFEC* | transcription factor EC | 4.02 | 0.000 | Nucleus |
| *TRIM69* | tripartite motif containing 69 | 3.94 | 0.030 | Nucleus |
| *IL2RB* | interleukin 2 receptor subunit beta | 3.92 | 0.001 | Plasma membrane |
| *CCND2* | cyclin D2 | 3.91 | 0.013 | Nucleus |
| *CDT1* | chromatin licensing and DNA replication factor 1 | 3.88 | 0.044 | Nucleus |
| *TP53I3* | tumor protein p53 inducible protein 3 | 3.84 | 0.005 | Cytoplasm |
| *CKS2* | CDC28 protein kinase regulatory subunit 2 | 3.79 | 0.004 | Other |
| *IFI44L* | interferon induced protein 44 like | 3.79 | 0.005 | Nucleus |
| *EBI3* | Epstein-Barr virus induced 3 | 3.71 | 0.000 | Extracellular Space |
| *PTP4A3* | protein tyrosine phosphatase type IVA, member 3 | 3.70 | 0.007 | Plasma membrane |
| *MCOLN2* | mucolipin 2 | 3.66 | 0.000 | Plasma membrane |
| *TXN* | thioredoxin | 3.62 | 0.000 | Cytoplasm |
| *MIR3142HG* |  | 3.59 | 0.000 | Other |
| *UTS2B* | urotensin 2B | 3.57 | 0.015 | Extracellular Space |
| *NLGN4X* | neuroligin 4, X-linked | 3.52 | 0.019 | Plasma membrane |
| *CDC45* | cell division cycle 45 | 3.48 | 0.045 | Nucleus |
| *COL4A3* | collagen type IV alpha 3 | 3.42 | 0.000 | Extracellular Space |
| *LOC101928433* | uncharacterized LOC101928433 | 3.41 | 0.000 | Other |
| *CBFA2T3* | CBFA2/RUNX1 translocation partner 3 | 3.40 | 0.000 | Nucleus |
| *SOCS3* | suppressor of cytokine signaling 3 | 3.39 | 0.022 | Cytoplasm |
| *TMEM163* | transmembrane protein 163 | 3.35 | 0.001 | Cytoplasm |
| *IFIT1* | interferon induced protein with tetratricopeptide repeats 1 | 3.35 | 0.002 | Cytoplasm |
| *TRIM47* | tripartite motif containing 47 | 3.35 | 0.000 | Cytoplasm |
| *TNFSF14* | tumor necrosis factor superfamily member 14 | 3.29 | 0.046 | Extracellular Space |
| *BCL2A1* | BCL2 related protein A1 | 3.28 | 0.000 | Cytoplasm |
| *FOXD1* | forkhead box D1 | 3.25 | 0.001 | Nucleus |
| *PLEKHG7* | pleckstrin homology and RhoGEF domain containing G7 | 3.23 | 0.016 | Other |
| *RORA* | RAR related orphan receptor A | 3.22 | 0.018 | Nucleus |
| *ATOX1* | antioxidant 1 copper chaperone | 3.17 | 0.000 | Cytoplasm |
| *TNNC2* | troponin C2, fast skeletal type | 3.16 | 0.000 | Cytoplasm |
| *PACSIN1* | protein kinase C and casein kinase substrate in neurons 1 | 3.16 | 0.001 | Cytoplasm |
| *C19orf48* | chromosome 19 open reading frame 48 | 3.15 | 0.049 | Other |
| *NCF2* | neutrophil cytosolic factor 2 | 3.13 | 0.002 | Cytoplasm |
| *AAGAB* | alpha- and gamma-adaptin binding protein | 3.13 | 0.025 | Cytoplasm |
| *CAMP* | cathelicidin antimicrobial peptide | 3.10 | 0.001 | Cytoplasm |
| *LRFN3* | leucine rich repeat and fibronectin type III domain containing 3 | 3.10 | 0.001 | Plasma membrane |
| *TCTEX1D2* | Tctex1 domain containing 2 | 3.10 | 0.000 | Other |
| *CD1C* | CD1c molecule | 3.10 | 0.001 | Plasma membrane |
| *IGF1* | insulin like growth factor 1 | 3.09 | 0.032 | Extracellular Space |
| *CD300A* | CD300a molecule | 3.08 | 0.000 | Plasma membrane |
| *ADPRH* | ADP-ribosylarginine hydrolase | 3.07 | 0.000 | Other |
| *BBC3* | BCL2 binding component 3 | 3.07 | 0.017 | Cytoplasm |
| *BIRC5* | baculoviral IAP repeat containing 5 | 3.06 | 0.025 | Cytoplasm |
| *JCHAIN* | joining chain of multimeric IgA and IgM | 3.06 | 0.002 | Extracellular Space |
| *PDE2A* | phosphodiesterase 2A | 3.05 | 0.000 | Cytoplasm |
| *BATF* | basic leucine zipper ATF-like transcription factor | 3.04 | 0.000 | Nucleus |
| *LGALSL* | lectin, galactoside binding like | 3.01 | 0.001 | Other |
| *XCL1* | X-C motif chemokine ligand 1 | 3.01 | 0.030 | Extracellular Space |
| *CISH* | cytokine inducible SH2 containing protein | 2.99 | 0.000 | Cytoplasm |
| *E2F1* | E2F transcription factor 1 | 2.97 | 0.012 | Nucleus |
| *ZWINT* | ZW10 interacting kinetochore protein | 2.97 | 0.010 | Nucleus |
| *LIMS2* | LIM zinc finger domain containing 2 | 2.96 | 0.004 | Cytoplasm |
| *ARID3A* | AT-rich interaction domain 3A | 2.91 | 0.000 | Nucleus |
| *HMOX1* | heme oxygenase 1 | 2.89 | 0.005 | Cytoplasm |
| *MRPL42P5* | mitochondrial ribosomal protein L42 pseudogene 5 | -2.71 | 0.000 | Other |
| *LOC100505771* | uncharacterized LOC100505771 | -2.72 | 0.000 | Other |
| *MXI1* | MAX interactor 1, dimerization protein | -2.73 | 0.000 | Nucleus |
| *OTUD1* | OTU deubiquitinase 1 | -2.76 | 0.000 | Other |
| *ICOSLG/LOC102723996* | inducible T-cell co-stimulator ligand | -2.78 | 0.000 | Plasma membrane |
| *SATB1* | SATB homeobox 1 | -2.80 | 0.002 | Nucleus |
| *TMEM123* | transmembrane protein 123 | -2.80 | 0.000 | Plasma membrane |
| *PI4KAP2* | phosphatidylinositol 4-kinase alpha pseudogene 2 | -2.83 | 0.006 | Other |
| *ARHGAP22* | Rho GTPase activating protein 22 | -2.83 | 0.001 | Cytoplasm |
| *MAGED4/MAGED4B* | melanoma antigen family D4B | -2.83 | 0.002 | Other |
| *TAGLN* | transgelin | -2.83 | 0.000 | Cytoplasm |
| *CMTM3* | CKLF like MARVEL transmembrane domain containing 3 | -2.84 | 0.000 | Extracellular Space |
| *LOC728392* | uncharacterized LOC728392 | -2.84 | 0.003 | Other |
| *MGAT5B* | mannosyl (α-1,6-)-glycoprotein β-1,6-N-acetyl-glucosaminyltransferase | -2.85 | 0.003 | Cytoplasm |
| *P2RX1* | purinergic receptor P2X 1 | -2.85 | 0.000 | Plasma membrane |
| *IGHV3OR16-10* | immunoglobulin heavy variable 3/OR16-10 (non-functional) | -2.86 | 0.003 | Other |
| *AMT* | aminomethyltransferase | -2.87 | 0.000 | Cytoplasm |
| *LOC643733* | caspase 4, apoptosis-related cysteine peptidase pseudogene | -2.87 | 0.007 | Other |
| *ADAM28* | ADAM metallopeptidase domain 28 | -2.91 | 0.003 | Plasma membrane |
| *STARD9* | StAR related lipid transfer domain containing 9 | -2.94 | 0.000 | Cytoplasm |
| *KCNH8* | potassium voltage-gated channel subfamily H member 8 | -2.95 | 0.003 | Plasma membrane |
| *PI4KAP1* | phosphatidylinositol 4-kinase alpha pseudogene 1 | -2.97 | 0.001 | Other |
| *ZNF112* | zinc finger protein 112 | -2.98 | 0.000 | Nucleus |
| *GABBR1* | gamma-aminobutyric acid type B receptor subunit 1 | -2.98 | 0.000 | Plasma membrane |
| *BTG1* | B-cell translocation gene 1, anti-proliferative | -2.99 | 0.000 | Nucleus |
| *MARCH3* | membrane associated ring-CH-type finger 3 | -3.00 | 0.000 | Cytoplasm |
| *WASIR1* | WASH and IL9R antisense RNA 1 | -3.01 | 0.024 | Other |
| *RASGRP2* | RAS guanyl releasing protein 2 | -3.01 | 0.002 | Cytoplasm |
| *LOC100652777* | group 10 secretory phospholipase A2-like | -3.01 | 0.002 | Other |
| *ZNF204P* | zinc finger protein 204, pseudogene | -3.02 | 0.002 | Other |
| *FAM129C* | family with sequence similarity 129 member C | -3.04 | 0.026 | Other |
| *SMA4* | glucuronidase, beta pseudogene | -3.08 | 0.000 | Other |
| *SLC6A16* | solute carrier family 6 member 16 | -3.08 | 0.001 | Plasma membrane |
| *BRF1* | BRF1, RNA polymerase III transcription initiation factor 90 kDa subunit | -3.09 | 0.000 | Nucleus |
| *APLP2* | amyloid beta precursor like protein 2 | -3.11 | 0.000 | Cytoplasm |
| *IGHV4-59* | immunoglobulin heavy variable 4-59 | -3.12 | 0.011 | Other |
| *IL4R* | interleukin 4 receptor | -3.13 | 0.000 | Plasma membrane |
| *SATB1-AS1* | SATB1 antisense RNA 1 | -3.13 | 0.001 | Other |
| *LOC145474* | uncharacterized LOC145474 | -3.14 | 0.029 | Other |
| *NLRP1* | NLR family, pyrin domain containing 1 | -3.19 | 0.000 | Cytoplasm |
| *CXCR4* | C-X-C motif chemokine receptor 4 | -3.19 | 0.000 | Plasma membrane |
| *ST3GAL1* | ST3 beta-galactoside alpha-2,3-sialyltransferase 1 | -3.23 | 0.000 | Cytoplasm |
| *CERK* | ceramide kinase | -3.23 | 0.000 | Plasma membrane |
| *H1FX* | H1 histone family member X | -3.24 | 0.003 | Nucleus |
| *JMJD7-PLA2G4B* | JMJD7-PLA2G4B readthrough | -3.26 | 0.015 | Cytoplasm |
| *PLPP5* | phospholipid phosphatase 5 | -3.27 | 0.000 | Other |
| *MOXD1* | monooxygenase DBH like 1 | -3.28 | 0.012 | Cytoplasm |
| *MIR600HG* | MIR600 host gene | -3.30 | 0.000 | Other |
| *SYT17* | synaptotagmin 17 | -3.30 | 0.048 | Plasma membrane |
| *C14orf132* | chromosome 14 open reading frame 132 | -3.32 | 0.000 | Other |
| *TCTN1* | tectonic family member 1 | -3.33 | 0.000 | Extracellular Space |
| *RNF24* | ring finger protein 24 | -3.37 | 0.000 | Other |
| *GCNT1* | glucosaminyl (N-acetyl) transferase 1, core 2 | -3.37 | 0.000 | Cytoplasm |
| *BCL6* | B-cell CLL/lymphoma 6 | -3.39 | 0.013 | Nucleus |
| *ICAM2* | intercellular adhesion molecule 2 | -3.41 | 0.000 | Plasma membrane |
| *HOMER2* | homer scaffolding protein 2 | -3.42 | 0.000 | Plasma membrane |
| *PCNA-AS1* | PCNA antisense RNA 1 | -3.44 | 0.000 | Other |
| *GYLTL1B* | glycosyltransferase-like 1B | -3.45 | 0.000 | Cytoplasm |
| *STMN3* | stathmin 3 | -3.48 | 0.004 | Nucleus |
| *FXYD7* | FXYD domain containing ion transport regulator 7 | -3.49 | 0.000 | Plasma membrane |
| *FCMR* | Fc fragment of IgM receptor | -3.53 | 0.008 | Plasma membrane |
| *NUAK2* | NUAK family kinase 2 | -3.68 | 0.004 | Other |
| *MID1IP1* | MID1 interacting protein 1 | -3.74 | 0.003 | Cytoplasm |
| *CLEC2B* | C-type lectin domain family 2 member B | -3.77 | 0.001 | Plasma membrane |
| *MEGF6* | multiple EGF like domains 6 | -3.86 | 0.004 | Cytoplasm |
| *FAM26F* | family with sequence similarity 26 member F | -3.89 | 0.027 | Other |
| *TAPT1* | transmembrane anterior posterior transformation 1 | -3.90 | 0.000 | Plasma membrane |
| *PDK4* | pyruvate dehydrogenase kinase 4 | -3.96 | 0.000 | Cytoplasm |
| *P2RY14* | purinergic receptor P2Y14 | -3.98 | 0.000 | Plasma membrane |
| *GLUL* | glutamate-ammonia ligase | -4.01 | 0.000 | Cytoplasm |
| *ARRDC2* | arrestin domain containing 2 | -4.02 | 0.000 | Other |
| *ZBTB16* | zinc finger and BTB domain containing 16 | -4.21 | 0.000 | Nucleus |
| *TRABD2A* | TraB domain containing 2A | -4.21 | 0.000 | Plasma membrane |
| *DENND3* | DENN domain containing 3 | -4.28 | 0.000 | Other |
| *GPR155* | G protein-coupled receptor 155 | -4.29 | 0.028 | Plasma membrane |
| *TLE2* | transducin like enhancer of split 2 | -4.32 | 0.000 | Nucleus |
| *ABCB1* | ATP binding cassette subfamily B member 1 | -4.33 | 0.000 | Plasma membrane |
| *IGHD* | immunoglobulin heavy constant delta | -4.39 | 0.000 | Extracellular Space |
| *BTLA* | B and T lymphocyte associated | -4.46 | 0.014 | Plasma membrane |
| *DPEP2* | dipeptidase 2 | -4.56 | 0.008 | Plasma membrane |
| *AGPAT5* | 1-acylglycerol-3-phosphate O-acyltransferase 5 | -4.56 | 0.000 | Cytoplasm |
| *CNTNAP2* | contactin associated protein-like 2 | -4.65 | 0.000 | Plasma membrane |
| *DGKD* | diacylglycerol kinase delta | -4.75 | 0.000 | Cytoplasm |
| *PDE3B* | phosphodiesterase 3B | -4.82 | 0.000 | Cytoplasm |
| *HVCN1* | hydrogen voltage gated channel 1 | -4.89 | 0.000 | Plasma membrane |
| *TCL6* | T-cell leukemia/lymphoma 6 (non-protein coding) | -4.92 | 0.000 | Other |
| *YBX3* | Y-box binding protein 3 | -5.20 | 0.003 | Nucleus |
| *FOXP1-IT1* | FOXP1 intronic transcript 1 | -6.06 | 0.000 | Other |
| *SKAP1* | src kinase associated phosphoprotein 1 | -6.65 | 0.001 | Cytoplasm |
| *FAM69B* | family with sequence similarity 69 member B | -6.91 | 0.025 | Other |
| *IL3RA* | interleukin 3 receptor subunit alpha | -7.31 | 0.000 | Plasma membrane |
| *LARGE* | like-glycosyltransferase | -7.75 | 0.000 | Cytoplasm |
| *GABRA3* | gamma-aminobutyric acid type A receptor alpha3 subunit | -8.51 | 0.000 | Plasma membrane |
| *DSP* | desmoplakin | -8.88 | 0.000 | Plasma membrane |
| *CD200* | CD200 molecule | -9.63 | 0.000 | Plasma membrane |
| *SORL1* | sortilin-related receptor, L(DLR class) A repeats containing | -13.23 | 0.001 | Cytoplasm |
| *KCNG1* | K+ voltage-gated channel modifier subfamily G member 1 | -17.52 | 0.000 | Plasma membrane |
| *NSUN7* | NOP2/Sun RNA methyltransferase family member 7 | -17.54 | 0.000 | Other |
| *TCL1B* | T-cell leukemia/lymphoma 1B | -21.96 | 0.000 | Other |
| *TCL1A* | T-cell leukemia/lymphoma 1A | -47.79 | 0.002 | Nucleus |
