# Supplementary material for: mTOR intersects antibody-inducing signals from TACI in marginal zone B cells
Source: Nat Commun. 2017 Nov 13;8:1462. doi: 10.1038/s41467-017-01602-4 (PMC5684130; doi:10.1038/s41467-017-01602-4)
Supplement: Supplementary file 2 — Peer review file [file 41467_2017_1602_MOESM2_ESM.pdf]

Reviewers' comments:

Reviewer #1 (Remarks to the Author):

Sintes et al. provide a very comprehensive analysis of the influence of mTOR on human marginal zone B cell responses. They found that mTOR binds to the transmembrane activator and CAML interactor (TACI) via MyD88. Activation of mTOR enables proliferation IgG class switching and plasmablast differentiation. This is a nice and exhausting study and I do not have much to add.

One important aspect however, it that the authors show that mTOR binds to MyD88 and TACI; however, they never investigate whether the complete complex of mTORC1 including Raptor binds.

The interaction studies were done mostly in HEK cells, so it is not clear how relevant this is for B cells.

A lot of functional studies is performed with rapamycin, however, this does not allow the conclusion that it is the mTOR that is bound to TACI mediates the effects and not some downstream mTOR that e.g. sits on the lysosome.

Reviewer #2 (Remarks to the Author):

In their study, Sintes et al. propose that mTOR signals downstream of TACI enhance marginal zone B cell responses.

In their study, the authors provide a very thorough characterization of human marginal zone B cells (MZB). They provide evidence that these cells are characterized by elevated mTOR activity and an "mTOR genes signature". They go on to show that TCAI signalling activates NfkB via mTOR signalling. Finally, they demonstrate that TACI and TLR cooperatively activate human MZB via mTORc1. Overall, this manuscript goes very far in characterising human MZ B cells in comparison to FO B cells, in particular focussing on the relationship between MZ B cells and mTOR.

MZ B cells are known to be 'primed' to respond very fast and efficiently in a T cell independent manner to antigenic challenge by differentiating rapidly into antibody-secreting plasma cells. However, the underlying reason for this heightened responsiveness is not known. The authors propose that increased mTOR activity in response to increased TACI expression is part of the equation.

This is a very dense manuscript, which at times suffers from too much detail. It would help to streamline the description of the data and narrative.

One of the problems that I had with this study was the question whether the dependency on mTOR was specific to MZ B cells, which is suggested but not really shown. In the absence of mTOR in mature B cells, are T cell dependent humoral responses impaired as well? What happens in mice lacking mTOR in mature B cells after immunization with T cell dependent antigens. Suppl. Fig. 6e would suggest that all B cells are dependent on mTOR for plasma cell differentiation. Is that the case? What happens in TACI-deficient MZ B cells? Do they lose their mTOR signature and their heightened state of responsiveness? How does the hyper responsiveness of TACI-KO B cells (Yan et al Nature Immunology 2, 638 – 64, 2001) fit into the model? TACI-KO B cells

Another general problem that I see with several of the experiments is the question how specific the experiments are that utilize rapamycin to inhibit mTOR. It is very difficult to judge whether mTOR inhibition specifically leads inhibition of class switch and plasma blast differentiation OR simply blocks proliferation (which it does in most cell types at sufficiently high levels). Both class

switch and plasma blast differentiation are proliferation dependent and thus will be blocked if proliferation is blocked. The authors should titrate rapamycin and test (the potentially different) sensitivity of these processes to mTOR blockade. It should also be remembered that rapamycin is not completely mTOR specific but blocks other kinases if applied at high amounts.

The authors state that disruption of TACI-mTOR interaction by rapamycin hampered IgG production to TI antigens. However, feeding mice rapamycin does not differentiate between B cell and T cell intrinsic mTOR inhibition. Some of the defects observed may have to do with overall immune suppression by rapamycin. What happens in response to TD antigens?

Throughout the manuscript, the authors have performed several Gene set enrichment analyses. They also rely heavily on relatively derived analyses of genes expression data such as NES correlation depicted in heatmaps (eg in Fig. 2b). Unfortunately, the descriptions of how these analyses were performed is missing from the method section. Furthermore, it is unclear where the data sets that underly the GSET come from (eg the plasma cell signature mentioned on page 10). These analyses need to be described in detail. Overall, it would be preferable to also provide data more directly in classical heatmaps showing expression data. Figure 2b is a good example why I have a problem with these analyses. The heatmaps suggests that there are at least 16 pathways regulated when MZ B cells are exposed to APRIL, and one of the major pathways is supposed to be mTOR. However, when you look at Suppl. Table 2, there are only 12 genes that show >1.5 fold change in response to APRIL and none of these genes is associated with mTOR! I do not doubt that APRIL can activate mTOR but I am not convinced that the microarray data show this.

Minor points:

On page 7, the authors state that MZB cells “show gene remodelling” liked to mTOR. It is unclear to me how mTOR may be involved in ‘gene remodelling’, which I would argue is an epigenetic process. Perhaps what is shown here is a gene signature that correlates with mTOR signalling or is consistent with increased mTOR activity.

I am not sure about the intracellular detection of BLIMP1 by FCM in Suppl. 1e. The authors should demonstrate that the method is specific by showing naïve FO B cells as negative control, and plasma cells as positive control. It is difficult to believe (but not impossible) that the minute shift detected in MZ B cells is specific.

On page three of the introduction the authors state that “BLIMP-1 transcriptionally suppresses paired-box containing-5 (PAX5)-orchestrated B cell identity programs involved in B cell proliferation, CSR and SHM. However, these processes remain partly active in PBs due to their lower expression of BLIMP-1 compared to PCs.” This is not correct. CSR and SHM are turned off in plasma blasts as is AID (compare ref 14).

The authors use mouse models that have been published before. There is no need to describe and figure the details of the targeting strategy again in Suppl. data. There does not seem to be a reference for CD21-Cre strain.

Figure 1C is not informative. It is unclear what the heatmap shows, presumably z-score expression data (not explained). However, with only two states compared (FO and MZB), the only color that one can get in such maps is red and blue. Thus, there is no real information here about expression levels.

Reviewer #1

Sintes et al. provide a very comprehensive analysis of the influence of mTOR on human marginal zone B cell responses. They found that mTOR binds to the transmembrane activator and CAML interactor (TACI) via MyD88. Activation of mTOR enables proliferation IgG class switching and plasmablast differentiation. This is a nice and exhausting study and I do not have much to add.

We thank the Reviewer for his/her words of appreciation for our work.

One important aspect however, it that the authors show that mTOR binds to MyD88 and TACI; however, they never investigate whether the complete complex of mTORC1 including Raptor binds.

Immunoprecipitation followed by immunoblotting suggested that indeed TACI recruits Raptor, a key element of the mTORC1 complex (**Figure A**). This physical interaction paralleled phenotypic (**Figure 1i-k)**, transcriptional (**Figures 1f,g** and **2c**) and functional properties (**Figure 2d-f**) indicating recruitment and activation of the mTORC1 complex by TACI. This observation is now described as data not shown in the revised manuscript (page 8).

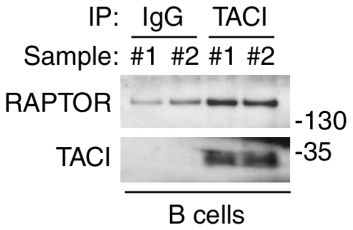

Figure A. TACI interacts with Raptor in human B cells. IB of Raptor and TACI following IP with anti-TACI or irrelevant IgG antibodies of protein lysates from human B cells. Data summarize one representative experiment with two biological replicates.

The interaction studies were done mostly in HEK cells, so it is not clear how relevant this is for B cells.

Interaction of TACI with mTOR and MyD88 was firstly assessed in freshly isolated human splenic B cells. 1) **Figure 2g** illustrates the formation of the TACI/MyD88/mTOR complex in human splenic B cells. 2) **Figure 2i** shows that there is no formation of the TACI/MyD88/mTOR complex in a common variable immunodeficient (CVID) patient carrying the TACI mutation S194X, which truncates the TACI cytoplasmic tail. 3) Additionally, **Figure 6d** confirms the interaction of TACI with mTOR, TLR9 and MyD88 in human MZ B cells stimulated with APRIL in the presence or absence of CpG-DNA, a TLR9 ligand. Subsequently, we used specific HEK293 transfectants to define which domains of TACI, mTOR and MyD88 are involved in protein-protein interactions. HEK293 cells were an ideal tool that allowed us to conclude that the MyD88-binding site of TACI may recruit mTOR via an NF-κB-inducing mechanism involving binding of the TIR domain of MyD88 to the FAT domain of mTOR (**Figure 3**).

A lot of functional studies is performed with rapamycin, however, this does not allow the conclusion that it is the mTOR that is bound to TACI mediates the effects and not some downstream mTOR that e.g. sits on the lysosome.

We agree with the Reviewer that this is an important point that is now discussed in the revised manuscript (page 16). The possibility that mTORC1 inhibition by rapamycin could affect both TACI-dependent and TACI-independent B cell effector functions prompted us to perform the rapamycin-free in vitro experiments depicted in **Figure 3**. These experiments strongly indicate that NF-κB activation by TACI requires TACI interaction with mTOR. Indeed, this activation does not occur when B cells or 293 cells express TACI mutants such as D1, D2, S321R or C233G, which cannot recruit mTOR through MyD88. Consequently, these mutants cannot induce NF-κB-dependent effects such as antibody class switching.

Reviewer #2

In their study, the authors provide a very thorough characterization of human marginal zone B cells (MZB). They provide evidence that these cells are characterized by elevated mTOR activity and an "mTOR genes signature". They go on to show that TACI signalling activates NfkB via mTOR signalling. Finally, they demonstrate that TACI and TLR cooperatively activate human MZB via mTORc1. Overall, this manuscript goes very far in characterising human MZ B cells in comparison to FO B cells, in particular focussing on the relationship between MZ B cells and mTOR.

We thank the Reviewer for his/her encouraging words.

MZ B cells are known to be 'primed' to respond very fast and efficiently in a T cell independent manner to antigenic challenge by differentiating rapidly into antibody-secreting plasma cells. However, the underlying reason for this heightened responsiveness is not known. The authors propose that increased mTOR activity in response to increased TACI expression is part of the equation.

Indeed, our data indicate that mTOR activation by TACI contributes to the pre-activated state of MZ B cells.

This is a very dense manuscript, which at times suffers from too much detail. It would help to streamline the description of the data and narrative.

We thank the Reviewer for pointing out this issue. In the revised manuscript we strived to reduce those sections of the text where the description of data was too dense.

One of the problems that I had with this study was the question whether the dependency on mTOR was specific to MZ B cells, which is suggested but not really shown. In the absence of mTOR in mature B cells, are T cell dependent humoral responses impaired as well? What happens in mice lacking mTOR in mature B cells after immunization with T cell dependent antigens?

As implied by the Reviewer, recent studies show that B cell responses to TD protein antigens require mTOR (revised by Iwata et al. *Cytokine Growth Factor Rev*. 2017;35:47-62). In the Revised manuscript, these studies are more extensively discussed. In particular, Zhang *et al*. demonstrated that mTOR deficiency in B cells (Cd19-Cre model) impairs germinal center formation and decreases NP-specific antibody responses to the TD antigen NP-CGG (*J. Immunol.* 2013, 191:1692-1703). Moreover, Jones *et al*. showed impaired germinal center responses, plasma cell differentiation and antibody secretion in mice immunized with a TD antigen and carrying an inducible B cell-specific deletion of the gene encoding Raptor, a key component of the mTORC1 complex (*J. Clin. Invest.* 2016, 126;4250–4261). Furthermore, Ersching *et al*. recently demonstrated that abnormally increased mTORC1 signaling reduces antigen-driven B cell selection and germinal center competitiveness (*Immunity* 2017; 46:1045-1058). However, the function of mTOR in B cell responses to TI antigens remains less understood. Our Revised manuscript is exclusively dedicated to this specific aspect of humoral immunity. Nonetheless, some data obtained from a TD immunization strategy are now shown in **Supplementary Figure 6.**

***Suppl. Fig. 6e** would suggest that all B cells are dependent on mTOR for plasma cell differentiation. Is that the case?*

Suppl. Fig. 6e indicates that plasma cell differentiation of mouse splenic CD43-negative B cells exposed to TI stimuli (APRIL +/- LPS) is rapamycin-sensitive. Also TD plasma cell differentiation could require mTOR signaling. Indeed, plasma cells are depleted in mice with a B cell-specific mTOR deficiency (Zhang et al. *J. Immunol.* 2013, 191:1692-1703 and Jones et al, *J. Clin. Invest.* 2016, 126:4250–4261). Conversely, plasma cells are increased in mice with enhanced mTOR signaling due to deletion of the mTOR repressor protein TSC1 in B cells (Benhamron et al. *Mol. Cell. Biol.* 2015, 35:153–166).
To corroborate these results in human B cells, we stimulated splenic FO B cells for 5 days with a TD cocktail of stimuli, including CD40L, IL-21 and IL-10, in the presence or absence of rapamycin (**Figure B**). Similar to human and mouse MZ B cells (**Figures 6b** and **7g**), FO B cells differentiated into plasmablasts through a rapamycin-sensitive mechanism (**Figure Ba**). In addition, also B cell proliferation required mTOR (**Figure Bb**). Altogether, our data indicate that mTOR is essential for the induction of plasma cell differentiation by TI or TD signals, including TACI and CD40, respectively.

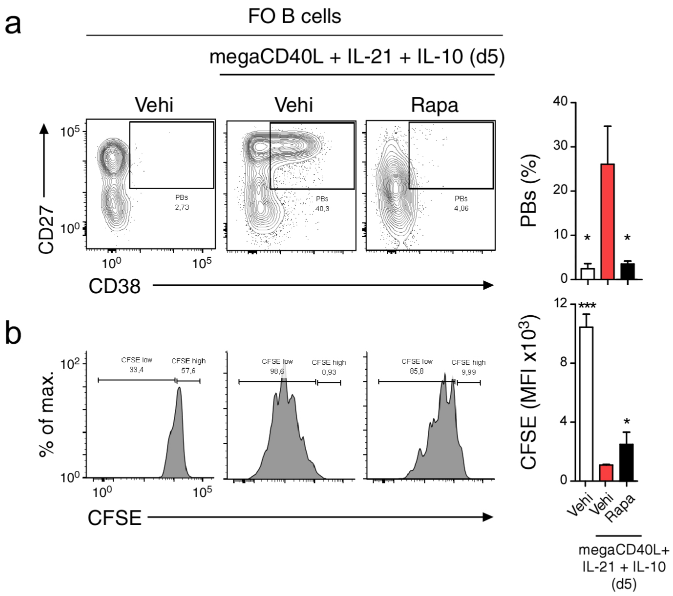

Figure B. Rapamycin inhibits TD plasmablast differentiation of human B cells. (**a**) Flow cytometry of viable $CD38^{+}CD27^{+}$ plasmablasts (PBs) generated by splenic FO B cells following stimulation with megaCD40L, IL-21 and IL-10 for 5 days. Barplot shows frequency of PBs (%) (**b**) Proliferation of FO B cells (identified by a low concentration of the cytosolic dye CFSE) stimulated as in (**a**). Barplot shows MFI of CFSE. Data summarize at least two experiments with at least two donors in each experimental group (**a**,**b**). Error bars, s.e.m.; $* \ p < 0.05$, $*** \ p < 0.001$ (two-tailed Student's t test).

What happens in TACI-deficient MZ B cells? Do they lose their mTOR signature and their heightened state of responsiveness? How does the hyper responsiveness of TACI-KO B cells (Yan et al Nature Immunology 2, 638 – 64, 2001) fit into the model?

This is another great question. In principle, we expected to find reduced mTOR signaling in TACI-deficient MZ B cells. Instead, flow cytometry demonstrated comparable p-S6 expression, a hallmark of mTORC1 activation, in MZ B cells from WT and *Tnfrsf13b*$^{-/-}$ mice (**Figure Ca**). Accordingly, B cells from WT and *Tnfrsf13b*$^{-/-}$ mice comparably expressed immune-activation (*Bach2, Zbtb32*) and mTORC1-related (*Tsc1, Slc7a5, Atf4*) transcripts (**Figure Cb**). Given that MZ B cells undergo BAFF-mediated hyper-activation in TACI-deficient mice (Mantchev et al. *J. Immunol.* 2007; 179:2282-2288, Tsuji et al. *Blood* 2011; 118:5832-5839, Ou et al. *Proc. Natl. Acad. Sci. U.S.A.* 2012; 109:15401-15406), we propose that BAFF hyper-production abnormally activates mTOR in TACI-deficient animals. Indeed, BAFF can activate mTOR by engaging a second B cell receptor known as BAFF-R (Woodland RT et al. *Blood*. 2008, 111:750-760; Otibopy KL et al. *Proc Natl Acad Sci USA*. 2008, 105:12435-12438; Zeng et al. *J Cell Physiol*. 2017, doi: 10.1002/jcp.25913). Consistent with this hypothesis, we found that BAFF serum levels were higher in *Tnfrsf13b*$^{-/-}$ mice compared to WT mice (**Figure Cc**). Hence, we concluded that TACI deficiency neither perturbs the mTOR gene signature of B cells nor diminishes their hyperexpansion due to increased BAFF-BAFF-R-mTOR-mediated signaling. These aspects are now discussed in the revised manuscript (page 16).

Figure C. Mouse B cells do not show reduced mTOR activity in the absence of TACI. (**a**) Flow cytometry of p(S235/S236)-S6 in mouse FO and MZ B cells from WT (white) and *Tnfrsf13b*$^{-/-}$ (blue) animals. (**b**) qRT-PCR of mRNAs for TSC1, BACH2, ZBTB32, SLC7A5 and ATF4 in MZ B cells from WT or *Tnfrsf13b*$^{-/-}$ mice. Results are normalized to mRNA for β-actin and presented as RE compared to a reference set of FO B cells. (**c**) Serum BAFF concentration in WT (white) and *Tnfrsf13b*$^{-/-}$ (blue) animals. Data depict two (a), four (b) or at least 12 biological replicates for each strain (c). Error bars, s.e.m.; *** $p < 0.001$ (two-tailed Student's t test).

Another general problem that I see with several of the experiments is the question how specific the experiments are that utilize rapamycin to inhibit mTOR. It is very difficult to judge whether mTOR inhibition specifically leads inhibition of class switch and plasma blast differentiation OR simply blocks proliferation (which it does in most cell types at sufficiently high levels). Both class switch and plasma blast differentiation are proliferation dependent and thus will be blocked if proliferation is blocked. The authors should titrate rapamycin and test (the potentially different) sensitivity of these processes to mTOR blockade. It should also be remembered that rapamycin is not completely mTOR specific but blocks other kinases if applied at high amounts.

This is another valuable question. To gain further insights into the specificity of the inhibitory effects induced by rapamycin, we measured MZ B cell proliferation, plasmablast differentiation and antibody class switching in the presence of decreasing concentrations of rapamycin. Flow cytometry showed that MZ B cells exposed to APRIL and CpG-DNA underwent less plasmablasts differentiation when exposed to a concentration of rapamycin as low as 1 nM (**Figure Da**). Accordingly, an identical amount of rapamycin inhibited the up-regulation of the plasmablast-inducing transcription factor BLIMP-1, but reversed the down-regulation of the plasmablast-suppressing transcription factor PAX5 (**Figure Db**). However, ELISA analysis of MZ B cell-secreted immunoglobulins indicated that CSR was partially blocked at even lower rapamycin doses (**Figure Dc**). Indeed, IgM-to-IgA CSR was reduced by 0.1 nM rapamycin, while IgM-to-IgG CSR was reduced by 0.01 nM rapamycin. Finally, similar to plasmablast differentiation, B cell proliferation was markedly reduced at a rapamycin concentration of 1 nM (**Figure Dd**).

These observations indicate that rapamycin reduces CSR through a mechanism partly independent of B cell proliferation. Our results are consistent with data published by others indicating that rapamycin inhibits CSR even at concentrations that preserve B cell proliferation (Keating et al. *Nat. Immunol.* 2013; 14(12):1266-76, Limon et al. *Proc. Natl. Acad. Sci. U.S.A.* 2014; 111(47): E5076–E5085.).

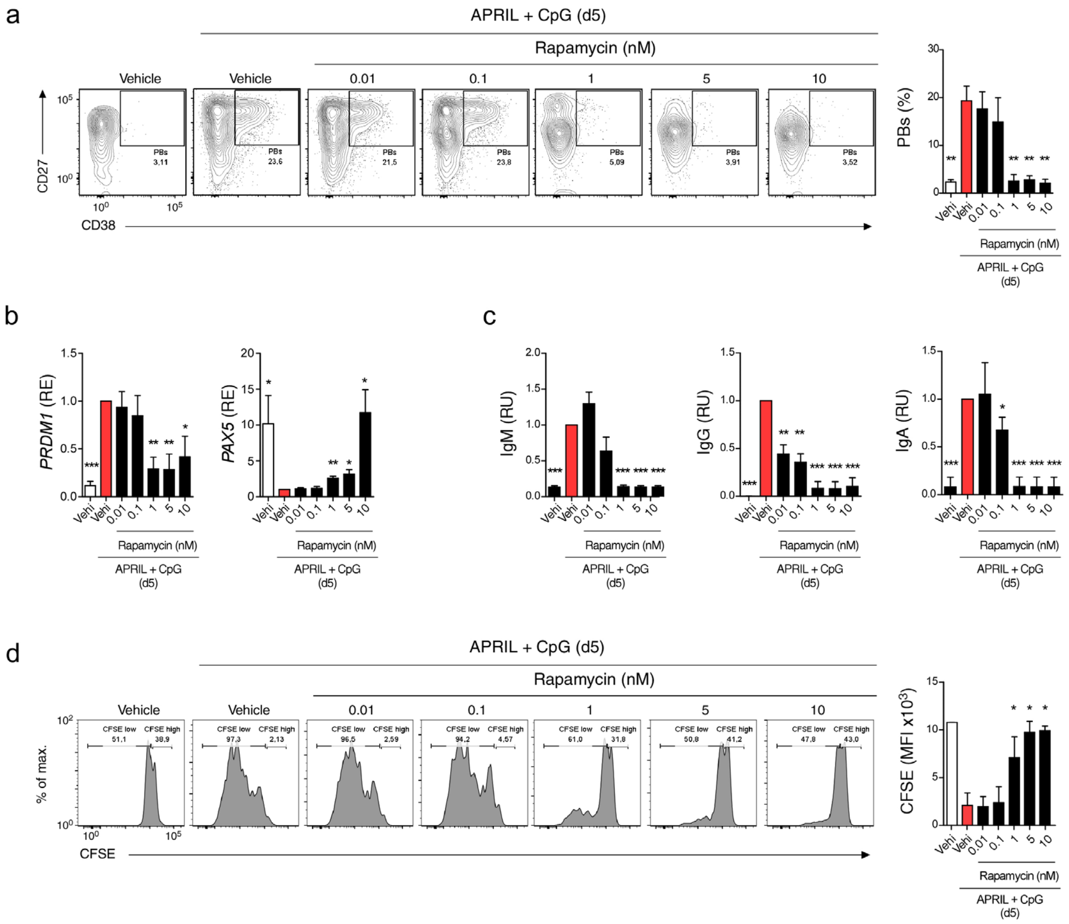

Figure D. Rapamycin can reduce CSR in human B cells independently of proliferation-related effects. (**a**) Flow cytometry of viable $CD38^+CD27^+$ plasmablasts (PBs) generated by human splenic MZ B cells following stimulation with APRIL plus CpG with or without different concentrations or rapamycin for 5 days. Barplot shows frequency of plasmablasts (%). (**b**) qRT-PCR of mRNAs for BLIMP-1 (*PRDM1*) and PAX-5 in MZ B cells stimulated as in (**a**). Results are normalized to mRNA for β-actin and presented as RE compared to stimulated cells without rapamycin (red bar). (**c**) ELISA of total IgM, IgG and IgA from cell supernatants obtained in (**a**). Results are normalized as relative units (RU) by comparing to stimulated cells without rapamycin (red bar). (**d**) Proliferation of MZ B cells (identified by a low concentration of the cytosolic dye CFSE) stimulated as in (**a**). Barplot shows MFI of CFSE. Data summarize at least two experiments with at least two replicates in each experimental group (**a-d**). Error bars, s.e.m.; $*$ $p < 0.05$, $**$ $p < 0.01$, $***$ $p < 0.001$ (two-tailed Student's t test).

The authors state that disruption of TACI-mTOR interaction by rapamycin hampered IgG production to TI antigens. However, feeding mice rapamycin does not differentiate between B cell and T cell intrinsic mTOR inhibition. Some of the defects observed may have to do with overall immune suppression by rapamycin. What happens in response to TD antigens?

We thank the reviewer for this insightful comment. Data on the effect of rapamycin in TD immune responses are now shown in **revised Supplementary Figure 6**. Rapamycin decreased TNP-specific IgM and IgG induction but not total antibody levels (**revised Supplementary Fig. 6a,b**). Rapamycin also impaired germinal center B cell differentiation, which is a hallmark of TD immune responses (**revised Supplementary Fig. 6c**). This last result mirrors recently published data showing that animals specifically lacking mTOR in B cells showed reduced germinal center formation following immunization with TD antigens (Zhang et al. *J. Immunol.* 2013, 191:1692-1703). More recently, mTORC1 has been shown to "pre-activate" germinal center B cells prior to dark zone proliferation (Ersching et al, *Immunity* 2017, 46:1045-1058). Additionally, blockade of mTOR by rapamycin reduced the number of TNP-specific plasmablasts following immunization with a TNP-haptenated TD antigen (**revised Supplementary Fig. 6d**). These observations have been discussed in the revised manuscript (page 18).

Throughout the manuscript, the authors have performed several Gene set enrichment analyses. They also rely heavily on relatively derived analyses of genes expression data such as NES correlation depicted in heatmaps (eg in Fig. 2b). Unfortunately, the descriptions of how these analyses were performed is missing from the method section. Furthermore, it is unclear where the data sets that underlie the GSEA come from (eg the plasma cell signature mentioned on page 10). These analyses need to be described in detail.

We thank the Reviewer for pointing out this issue. We expanded the description of how these analyses were performed in the **Revised Methods**. Figure 1g,h is now correctly cited in the revised manuscript. Gene expression Omnibus series (GSE) emerging from the GSEA analysis were obtained from the Broad Institute database (http://www.broadinstitute.org/gsea/index.jsp). GSE numbers have been included in the corresponding figure legend. For instance, PC signature comes from the comparison between Naïve B cells with Plasma cells (GSE22866).

Overall, it would be preferable to also provide data more directly in classical heatmaps showing expression data. ***Figure 2b*** *is a good example why I have a problem with these analyses.*

Numerous biologically relevant pathways emerged from the gene expression analysis of MZ B cells exposed to APRIL and/or rapamycin. Data was summarized as normalized enrichment score (NES) in Figure 2b. The magnitude of the NES increment depends on the correlation of the gene with the phenotype. Due to space limitations, we opted to include a summary of top differentially expressed genes as Suppl. Table 2. Of note, heatmaps including top differentially expressed genes related to main pathways described in our work like mTORC1

signaling (**Figure 2c**); NF-κB (**Figure 4a**) and Proliferation (**Figure 5d**) were already shown in the original manuscript.

The heatmaps suggests that there are at least 16 pathways regulated when MZ B cells are exposed to APRIL, and one of the major pathways is supposed to be mTOR. However, when you look at Suppl. Table 2, there are only 12 genes that show >1.5 fold change in response to APRIL and none of these genes is associated with mTOR! I do not doubt that APRIL can activate mTOR but I am not convinced that the microarray data show this.

Considering the short-term nature of the B cell-stimulation experiment employed to study the effect of rapamycin on APRIL-induced gene expression (3 hours), we were not expecting to observe dramatic gene expression changes. Instead, we aimed at focusing on the cumulative effect of small gene expression changes. Thus, we prioritized the Broad Institute GSEA's approach, which yielded biologically consistent results that were further validated by both in vitro and in vivo functional approaches.

Minor points:

On page 7, the authors state that MZB cells "show gene remodelling" liked to mTOR. It is unclear to me how mTOR may be involved in 'gene remodelling', which I would argue is an epigenetic process. Perhaps what is shown here is a gene signature that correlates with mTOR signalling or is consistent with increased mTOR activity.

We thank the Reviewer for this insightful comment. We agree that this statement could be misleading. In the revised manuscript we have replaced the subheading by "MZ B cells gene signature is consistent with mTORC1 activity" (page 7).

I am not sure about the intracellular detection of BLIMP1 by FCM in Suppl. 1e. The authors should demonstrate that the method is specific by showing naïve FO B cells as negative control, and plasma cells as positive control. It is difficult to believe (but not impossible) that the minute shift detected in MZ B cells is specific.

Revised Supplementary Figure 1e includes the intracellular staining of BLIMP-1 and ATF-4 in splenic plasmablasts (black line) so as to prove the specificity of the positive signal observed in MZ B cells (red line) compared to naïve FO B cells (blue line). Constitutive expression of BLIMP-1 by mouse MZ B cells has been previously reported by others (Martin et al. *Immunity* 2001,14:617-629).

On page three of the introduction the authors state that "BLIMP-1 transcriptionally suppresses paired-box containing-5 (PAX5)-orchestrated B cell identity programs involved in B cell proliferation, CSR and SHM. However, these processes remain partly active in PBs due to their lower expression of BLIMP-1 compared to PCs." This is not correct. CSR and SHM are turned off in plasma blasts as is AID (compare ref 14).

We thank the reviewer for this insightful comment. We have removed the second sentence from the revised manuscript.

The authors use mouse models that have been published before. There is no need to describe and figure the details of the targeting strategy again in Suppl. data. There does not seem to be a reference for CD21-Cre strain.

It is true that other mTOR gene-targeted deletion models in B cells have already been published (e.g. Zhang S. *et al, J. Immunol*. 2013), but this is the first time mTOR has been specifically deleted in mature B cells using the Cd21-Cre strain. That is the reason why the targeting strategy is illustrated in the **Supplementary Figure 6** and described in the Methods section.

***Figure 1C** is not informative. It is unclear what the heatmap shows, presumably z-score expression data (not explained). However, with only two states compared (FO and MZB), the only color that one can get in such maps is red and blue. Thus, there is no real information here about expression levels.*

We thank the Reviewer for pointing out this issue. **Revised Figure 1c** shows a blue/red color gradient corresponding to z-score expression data and it has been explained in the corresponding figure legend.

REVIEWERS' COMMENTS:

Reviewer #1 (Remarks to the Author):

The authors responded well to my queries. I do not have further comments.

Reviewer #2 (Remarks to the Author):

Sintes et al have substantially revised their manuscript and have addressed most questions and concerns raised. The majority of the additional data support the concept of the authors. However, reviewer figure C, apparently addressing the mTOR activation status of TACI-deficient B cells, was (inadvertently) omitted from the rebuttal. That made it difficult to assess the quality of these data.

However, several important pieces of data are only shown in reviewer figures. Some of them are crucial for the interpretation of the data and should be shown and discussed in the manuscript. In particular, the above-mentioned data concerning mTOR activity in TACI-deficient B cells is of interest. These data do not fit a simple model according to which TACI mediated mTOR activation is the major driver of the 're-activated' state of MZ B cells. The reviewer appreciates that BAFF and perhaps other molecules can activate mTOR in MZ B cells, and that it might be for this reason that TACI-deficient B cells do not show reduced mTOR activity. However, this is an important point that should be shown and discussed.

Similarly, the data shown in reviewer figure D are important. They dissect the functions of rapamycin with respect to class switching, PC differentiation and proliferation. It should be made clear that the impaired PC differentiation in response to rapamycin may indeed be a function of impaired proliferation and not necessarily a direct effect on Blimp1 expression or the actual differentiation process. These results are not adequately discussed.

I also would argue that the interaction of TACI and Raptor in Figure A should be shown if the authors are confident in their results. Alternatively, it should not be mentioned in the manuscript.

Additional point

The original studies that relate to the gene signatures used in this manuscript, should be cited, and care should be taken to provide the correct GSE numbers. For instance, the PC signature does not relate to GSE22866 as stated in the manuscript.

Reviewer #2

Sintes et al have substantially revised their manuscript and have addressed most questions and concerns raised. The majority of the additional data support the concept of the authors. However, reviewer figure C, apparently addressing the mTOR activation status of TACI-deficient B cells, was (inadvertently) omitted from the rebuttal. That made it difficult to assess the quality of these data. However, several important pieces of data are only shown in reviewer figures …

We thank the Reviewer for his/her positive comments. We agree with this Reviewer that showing the additional data from Figures A-D (see earlier point-by-point) may be important for the correct interpretation of the findings presented in the manuscript. The re-revised manuscript includes these additional data and discusses their relevance to the central conclusions of the work.

I also would argue that the interaction of TACI and Raptor in Figure A should be shown if the authors are confident in their results. Alternatively, it should not be mentioned in the manuscript.

We are confident in the results showing TACI-mTOR-RAPTOR interaction. However, given the overall wealth of the evidence documenting TACI signaling via mTORC1, we opted to omit TACI-mTOR-RAPTOR interaction data from the manuscript.

The original studies that relate to the gene signatures used in this manuscript, should be cited, and care should be taken to provide the correct GSE numbers. For instance, the PC signature does not relate to GSE22866 as stated in the manuscript.

Thank you for raising these points. The manuscript has been modified accordingly.
